# Predicting Prognosis and Distinguishing Cold and Hot Tumors in Bladder Urothelial Carcinoma Based on Necroptosis-Associated lncRNAs

**DOI:** 10.3389/fimmu.2022.916800

**Published:** 2022-07-04

**Authors:** Dongze Liu, Shengxian Xu, Taihao Chang, Shenfei Ma, Kaibin Wang, Guangyu Sun, Shuaiqi Chen, Yong Xu, Hongtuan Zhang

**Affiliations:** Department of Urology, National Key Specialty of Urology, Second Hospital of Tianjin Medical University, Tianjin Key Institute of Urology, Tianjin Medical University, Tianjin, China

**Keywords:** necroptosis, lncRNA, risk model, immunotherapy, clusters, hot and cold tumors

## Abstract

**Background:**

In reference to previous studies, necroptosis played an important role in cancer development. Our team decided to explore the potential prognostic values of long non-coding RNAs (lncRNAs) associated with necroptosis in bladder urothelial carcinoma (BLCA) and their relationship with the tumor microenvironment (TME) and the immunotherapeutic response for accurate dose.

**Methods:**

To obtain the required data, bladder urothelial carcinoma transcriptome data were searched from Cancer Genome Atlas (TCGA) (https://portal.gdc.cancer.gov/). We used co-expression analysis, differential expression analysis, and univariate Cox regression to screen out prognostic lncRNAs associated with necroptosis in BLCA. Then the least absolute shrinkage and selection operator (LASSO) was conducted to construct the necroptosis-associated lncRNAs model. Based on this model, we also performed the Kaplan–Meier analysis and time-dependent receiver operating characteristics (ROC) to estimate the prognostic power of risk score. Multivariate and univariate Cox regression analysis were performed to build up a nomogram. Calibration curves, and time-dependent ROC were also conducted to evaluate nomogram. Principal component analysis (PCA) revealed a difference between high- and low-risk groups. In addition, we explored immune analysis, gene set enrichment analyses (GSEA), and evaluation of the half-maximal inhibitory concentration (IC50) in constructed model. Finally, the entire samples were divided into three clusters based on model of necroptosis-associated lncRNAs to further compare immunotherapy in cold and hot tumors.

**Results:**

A model was built up based on necroptosis-associated lncRNAs. The model revealed good consistence between calibration plots and prognostic prediction. The area of 1-, 3-, and 5-year OS under the ROC curve (AUC) were 0.707, 0.679, and 0.675. Risk groups could be helpful for systemic therapy due to the markedly diverse IC50 between risk groups. To our delight, clusters could effectively identify cold and hot tumors, which would be beneficial to accurate mediation. Clusters 2 and 3 were considered the hot tumor, which was more sensitive to immunotherapeutic drugs.

**Conclusions:**

The outcomes of our study suggested that necroptosis-associated lncRNAs could effectively predict patients with BLCA prognosis, which may be helpful for distinguishing the cold and hot tumors and improving individual treatment of BLCA.

## Introduction

Bladder cancer (BLCA) is the fourth in the incidence of tumors in men and the most common of all urological malignant tumors worldwide. More importantly, morbidity and mortality of BLCA increase with time development (1–3). Currently, the median survival time for patients with advanced BLCA is only about 14 months, even after using available therapies for bladder cancer, such as chemotherapy, radiation, surgery, and bacillus Calmette-Guerin (BCG) (4). Therefore, new effective and available therapeutic strategies are urgently needed to be found. Immunotherapy has a bright future and achieves success in the treatment of malignancies. However, low response rates remain in the treatment of immune checkpoint therapy (ICP) (5). This result is mainly due to the lack or low infiltration of tumor T cells, leading to resistance to ICP inhibitors, which is characterized as “cold tumor” (6). Therefore, improvements in immunotherapy for BLCA are urgently needed.

Necroptosis, as a form of programmed cell death, exits downstream of PRK1 and RIPK3 and forms an oligomeric compound named necrosome (7). Necroptosis has been reported to play an inhibitory role in most tumors and has been found to improve antitumor immunogenicity by proliferation of CD8+ T cells *in vivo* and *in vitro* experiments (8–10). Similarly, a nanovaccine modeling necrotic tumor cells was recently reported to enhance antitumor immunity with immunostimulation and induced a significant proliferation of NKG2D+ natural killer (NK) cells and IFN-γ-expressing CD8+ T cells (9). These studies show that necroptosis has a bright future in tumor immunotherapy.

Important regulatory roles of long noncoding RNA (lncRNA) in different physiological processes and gene expression have been found in previous studies and have involved programmed cell death (11). LncCRLA was significantly upregulated in lung adeno- carcinoma and suppressed RIPK1-ralated necroptosis by impairing interaction between RIPK1 and RIPK3 (12). MiRNAs involved miR-9 and miR-185 were released by linc00176 to downregulate target mRNAs, which led to necroptosis of hepatocellular carcinoma cells (13). Previous research reported that lncRNA could alter the resistance of cancer cells to immune response, thus immune evasion occurred (14). In the present, relevant studies has not been identified whether necroptosis-related lncRNA can be regarded as a potential therapeutic target in BLCA. Hence, more knowledge about lncRNAs associated with necroptosis are needed to clearly understand the roles of them in immunotherapy.

Although checkpoint inhibitors have also made great advances in the immunotherapy, but low response rate remains a major problem in cold tumors that lack T-cell infiltration. Exploring the question of how to distinguish cold tumors and hot tumors and turn cold tumors into hot tumors will bring hope for antitumor immunotherapy (7). Currently, lncRNAs are also considered as potential biomarkers and therapeutic targets in several tumors (15, 16). According to necroptosis-associated lncRNAs, we tried to regroup patients and effectively distinguished hot tumors to improve prognosis and achieve precision medicine in clinical practice.

## Materials and Methods

### Inclusion and Exclusion Criteria

The transcriptome data and clinical data of BLCA were obtained from the TCGA database. In the analysis for different indicators of clinical data, we excluded the missing data of survival time, survival status, age, gender, grade, stage and all data of TNM, respectively. To reduce errors due to statistical bias, we also excluded data on survival times of less than 30 days.

### Information of Patients With BLCA Collection and Processing

Our team collected transcriptome profiling of 433 bladder samples (19 normal samples and 414 bladder tumors) with the format of HTSEQ-FPKM from the Cancer Genome Atlas (TCGA) database (https://portal.gdc.cancer.gov/). The clinical data for 413 bladder tumors were directly obtained from the TCGA clinical category. We then extracted, decompressed, and compiled the data using Perl and WinRAR software. The expression levels of mRNA and lncRNA from the TCGA database were also distinguished separately in the same way.

### Identification of Necroptosis-Associated Genes and lncRNAs in BLCA

Eight necroptosis genes were found in the necroptosis gene set M24779.gmt, which was downloaded from the Gene Set Enrichment Analysis (GSEA) (http://www.gsea-msigdb.org/gsea/index.jsp). Besides, we reviewed previous reports on necrotic cell death disease, and we finally got information on 67 genes associated with necroptosis disease (**Supplementary Appendix T1**). Additionally, Pearson’s correlation analysis was performed between each gene associated with necroptosis and the lncRNA expressed. Necroptosis-associated lncRNAs were finally identified when a correlation coefficient >0.5 and P<0.001 with limma R packages. Then we performed a differential analysis of the lncRNAs mentioned above, 439 differentially expressed lncRNAs were also identified (Log2 fold change (FC) > 1, p < 0.05) by limma packages. Finally, we merged necroptosis-associated genes and differentially expressed lncRNAs with our clinical data downloaded from TCGA.

### Construction and Evaluation of the Risk Signature for BLCA

The BLCA dataset from TCGA was randomly divided into a training risk set and a test risk set using the caret R package in a 1:1 ratio. The train set was used to construct necroptosis-associated lncRNA signatures, and the test set and the whole set were used to validate the signature. According to necroptosis-associated lncRNA expression and relevant clinical data, we conducted univariate Cox proportional hazard regression analysis to identify 34 survival-related lncRNAs for BLCA (*p* < 0.05). To prevent overfitting, we then subjected the Lasso regression to 10-fold cross-validation with a threshold of p-value <0.05 and 7 necroptosis-associated lncRNA were screened out to build risk model. Time-dependent receiver operating characteristic (ROC) curves for this model were plotted for 1, 3, and 5 years using a computational procedure. The detailed risk score using the following equation:


$$Risk\;score=\sum_{k=1}^{n}coef(lncRNA^{k})*expr\left(lncRNA^{k}\right)$$

The coef (*lncRNA^k^*) was the abbreviation for coefficient of lncRNAs associated with survival and expr (*lncRNA^k^*) represented the expression of lncRNAs. Subgroups including low- and high-risk groups were created based on the median risk score in the TCGA cohorts. The correlation between the model and clinical factors was then analyzed using the chi-square test to assess the prognostic value of the proposed model.

### Independence Risk Factors and ROC

Univariate Cox (uni-Cox) and multivariate Cox (multi-Cox) regression were performed to assess whether the risk score and clinical factors could be regarded as independent risk factors. Meanwhile, we plotted the ROC to compare the effects of different factors on prognosis.

### Nomogram and Calibration

We used tumor stage, age, and risk score to build a nomogram for 1-, 3-, and 5- year OS based on R (ver.4.1.2) *via* the rms, survival, and regplot R packages. We also set up the calibration curve to demonstrate whether the predicted results showed good consistency with the actual situation.

### Gene Set Enrichment Analyses

The gene set enrichment analyses (GSEA) software (https://www.gsea-msigdb.org/gsea/login.jsp) was utilized to find pathways significantly enriched in the risk groups with the gene set (kegg.v.7.5.1 symbols. gmt) and the threshold values were defined as *p* < 0.05 and FDR < 0.25.

### Evaluation of the Tumor Microenvironment (TME) and Immunological Analysis

Based on the outcome of GSEA, computational methods were applied to estimate the immune-cell effects in risk groups. The immune infiltration status of patients with BLCA was analyzed from multiple immune data platforms, including XCELL, TIMER, QUANTISEQ, CIBERSORT, MCPCOUNTER, EPIC, and CIBERSORT in TIMER2.0 (http://timer.cistrome.org/). On the other hand, the limma, ggpubr, and reshape2 R packages were used to analyze immune cells and immune function scores. limma, ggtext scales, tidyverse, ggplot2, and ggpubr R packages were utilized to analyze the correlation in various immune infiltrating cells with the wilcoxon test and the results were displayed in a bubble chart. With the ggpubr R package, TME scores and immune checkpoint activation status between the low- and high-risk groups were also compared.

### Exploration of Models in Clinical Treatment

We then assessed their treatment response using the R package pRRophetic based on our risk models gene expression levels and *in vitro* drug sensitivity in cell lines (17, 18).

### Clustering Based on Prognostic lncRNAs

To explore the response of BLCA to immunotherapy, we determined to evaluate potential molecular clusters based on the model of prognostic lncRNAs through the ConsensusClusterPlus (CC) R package. We also utilized the Rtsne R package to perform principal component analysis (PCA), Kaplan–Meier survival analysis and T-distributed stochastic neighbor embedding (t-SNE). Finally, immune-related analysis and drug sensitivity were estimated using ggpubr, ggplot2, and pRRophetic R packages.

## Results

### Identification of lncRNAs Associated With Necroptosis in BLCA Patients

**Figure 1:**
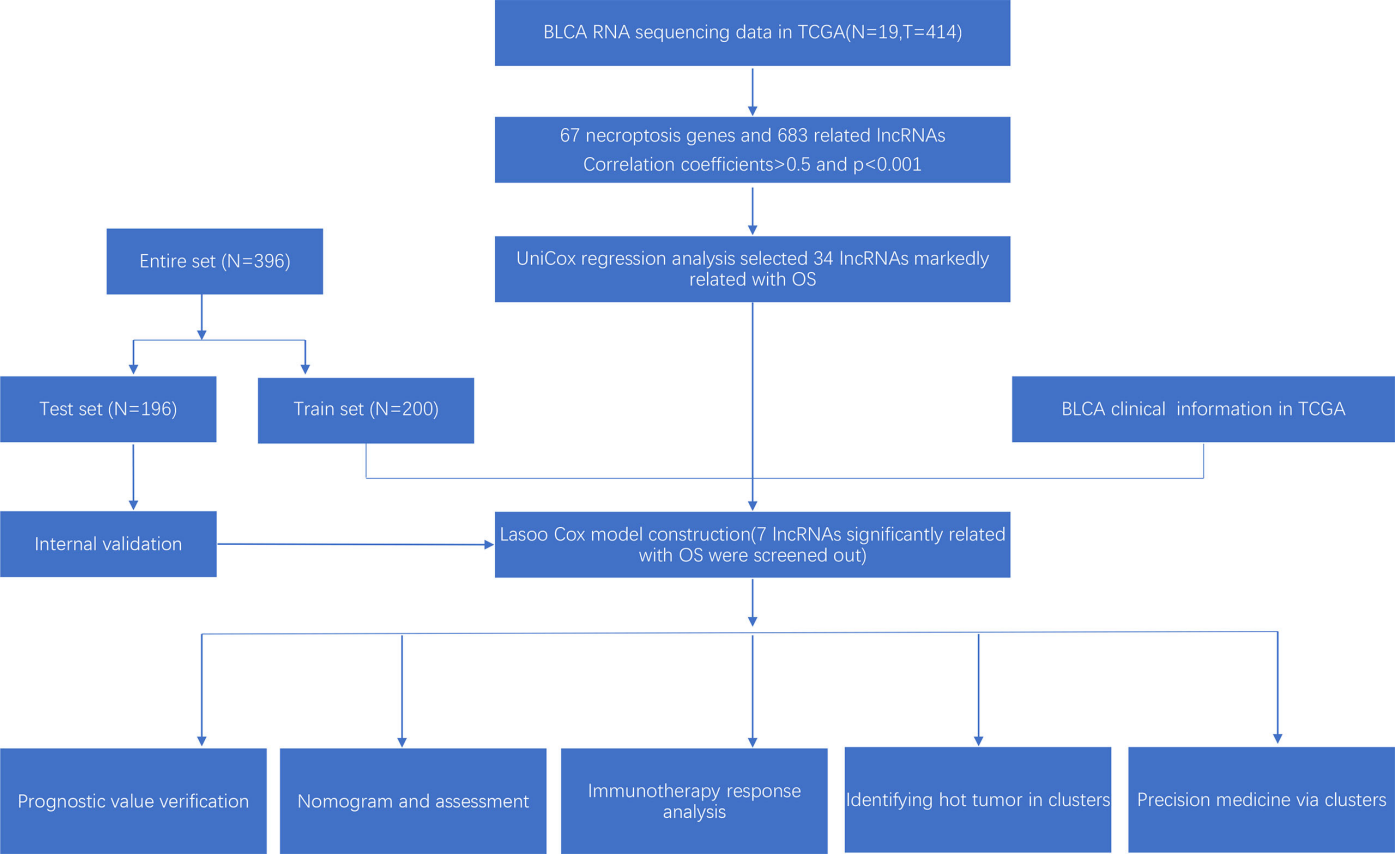
Research process.

**Figure 2:**
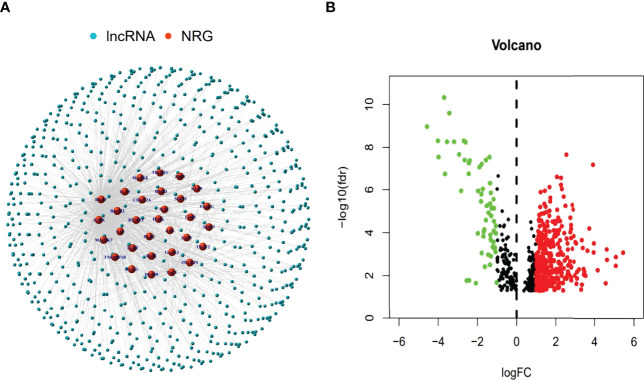
Identification of necroptosis-associated lncRNAs in BLCA patients. The co-expressed relationship between necroptosis genes (NRG) and lncRNAs (correlation coefficients > 0.5 and *p* < 0.001) **(A)**. The volcano plot of 439 necroptosis-associated lncRNAs with differential expression (|Log2FC| > 1, p < 0.05) **(B)**.

The research process was shown below (**Figure 1**). We extracted 19 normal samples and 414 tumor samples of BLCA from the Cancer Genome Atlas (TCGA) database. Then, we divided the gene expression data of BLCA into mRNA and lncRNA. Next, we cycled one lncRNA and one necroptosis-associated gene at a time from all lncRNA and 67 necroptosis-associated genes and performed a Pearson’s correlation test based on both expression levels in our tumor samples. Only the correlation coefficient >0.5 and the p-value <0.001, the corresponding lncRNA was saved for subsequent analysis. We finally acquired 682 necroptosis-associated lncRNAs (**Figure 2A** and **Supplementary Appendix T2**). Meanwhile, we performed differential expression analysis and 439 variously expressed lncRNAs (|Log2FC| > 1, *p* < 0.05 and FDR<0.05) were screened out between bladder cancer and normal tissues (**Figure 2B**).

### Construction of the Risk Assessment Model and Validation

**Figure 3:**
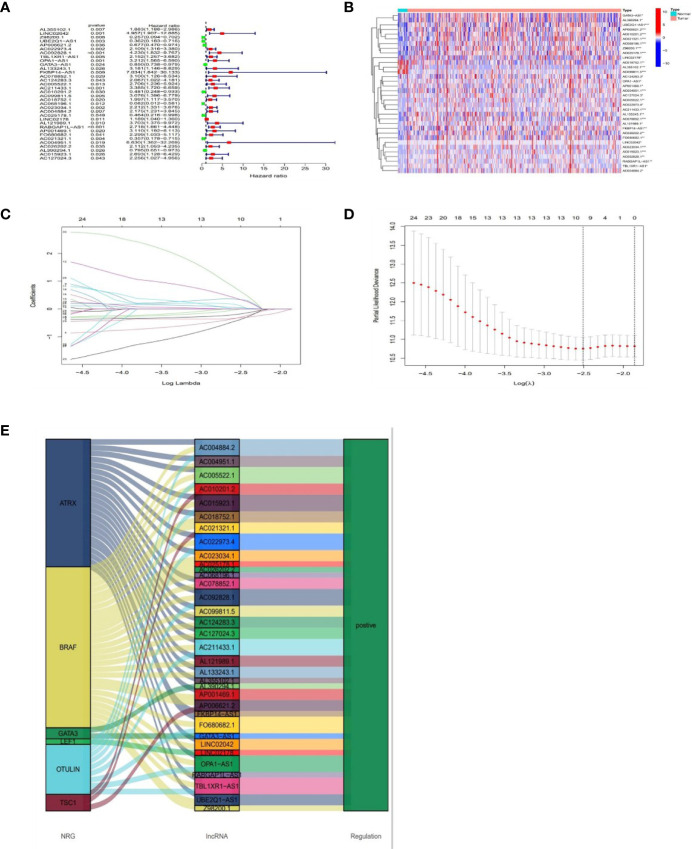
Prognosis of lncRNAs associated with necroptosis in BLCA. The forest plot shows prognostic lncRNAs using univariate Cox regression analysis (red:HR>1 green:HR<1) **(A)**. The heat map of 34 prognostic lncRNAs **(B)**. The 10-fold cross-validation for varying selection in the LASSO regression **(C)**. The profile of the LASSO coefficient of 7 necroptosis-associated lncRNAs **(D)**. The necroptosis genes and 34 related lncRNAs Sankey graph **(E)**. (*: p < 0.05, **: p < 0.01, and ***: p < 0.001).

Based on univariate Cox (uni-Cox) regression analysis, we discovered 34 lncRNAs associated with necroptosis were significantly related with overall survival (OS) (*p* < 0.05) and produced a forest map and a heat map (**Figures 3A, B**). 34 necroptosis-related lncRNAs and their 6 co-expression necroptosis genes were shown in the Sankey plot and there was a positive regulatory relationship between 34 lncRNAs and their co-expression necroptosis genes (**Figure 3E**). To prevent overfitting of our model, we executed the Lasso regression on these lncRNAs, and 7 lncRNAs associated with necroptosis in BLCA were extracted when the first-rank value of Log(*λ*) was the least deviation possibility (**Figures 3C, D**).

Multivariate Cox regression was applied to calculate the risk score with the fo-rmula: risk score = UBE2Q1-AS1 × (-0.8519) + FKBP14-AS1 × (1.6238) + AC068196.1 × (−1.8468) + AC025178.1 × (-0.6533) + LINC02178 × (0.1783) + AC021321.1 × (−0.7456) + AC004951.1 × (3.3758).

**Figure 4:**
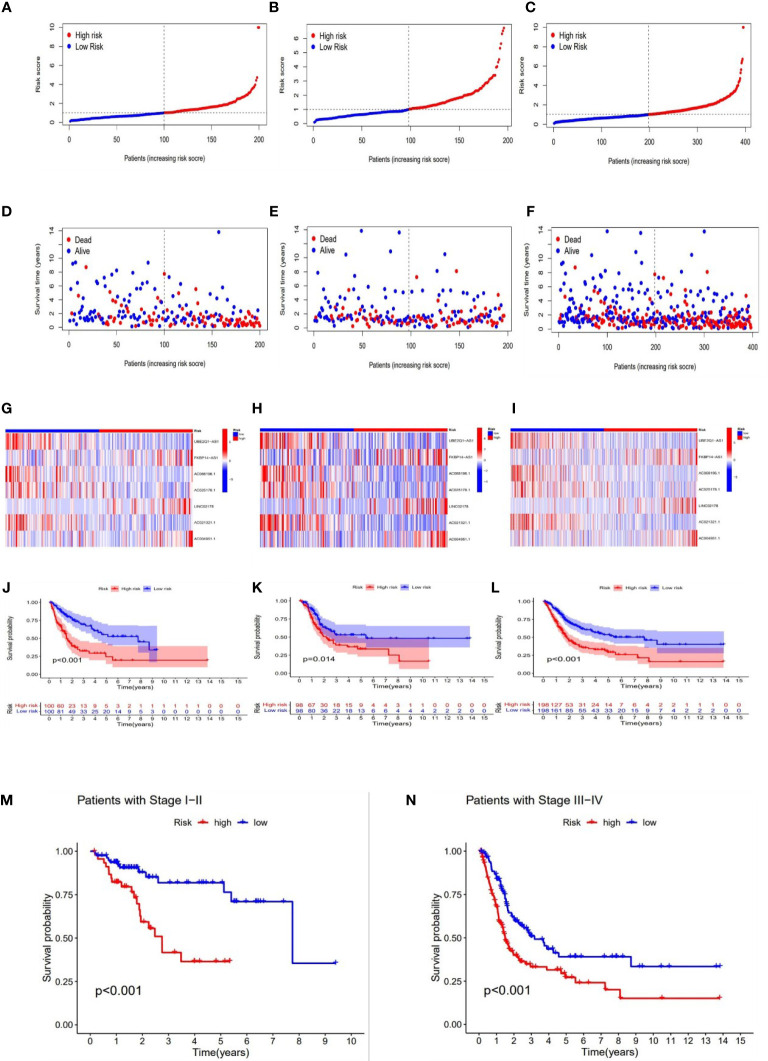
Prognosis value of the 7 necroptosis-associated lncRNAs model in the train, test, and full groups, respectively. The distribution of risk scores **(A–C)**, survival time and survival status in the train, test, and full groups **(D–F)**. Heat maps of 7 lncRNA expression in the train, test, and full groups **(G–I)**. Kaplan–Meier survival curves of survival probability (OS) of patients in the train, test, and full groups **(J–L)**. Kaplan–Meier survival curves of survival probability (OS) prognostic value for the constructed model in the full groups with tumor stage **(M–N)**.

With the results of the risk score formula, we divided the patients into low- and high- risk groups with the median risk score. We compared the distribution of risk score, patient survival status, survival time, and OS in low-risk and high-risk groups in the train, test, and full group, respectively. We found that high-risk groups had higher risk scores, shorter survival time, and lower survival probability. These results suggested a poorer prognosis in the high-risk group (**Figures 4A–L**). In addition, to validate our grouping model, the same results were also obtained for conventional tumor stage (**Figures 4M, N**).

### Construction of Nomogram

**Figure 5:**
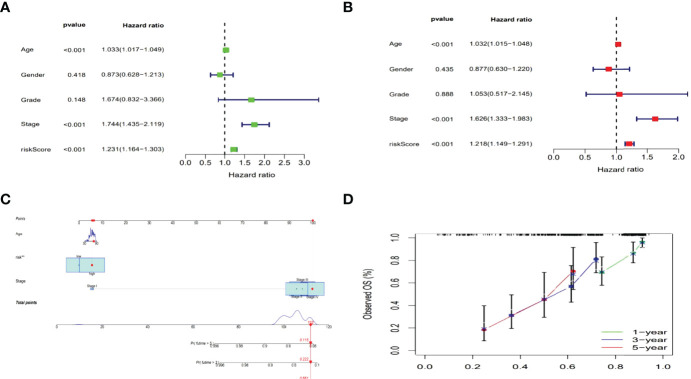
Nomogram of the risk model. Cox analyzes of clinical characteristics and risk score with OS (Uni- and multi-Cox in **A, B**). The nomogram that combined age, the risk score, and tumor stage forecasted the 1-, 3-, and 5-year OS probabilities. **(C)** The 1-, 3-, and 5-year OS calibration curves **(D)**.

The hazard ratio (HR) and 95% confidence interval (CI) for risk scores were1.231 and 1.164–1.303 (*p* < 0.001) in the univariate Cox regression and 1.218 and 1.149–1.291 (*p* <0.001) in the multivariate Cox regression, respectively. Besides, the other two independent prognostic indicators involved age (1.032 and 1.015-1.048; *p* <0.001) and stage (1.626 and 1.333–1.983; *p*<0.001) were also observed (**Figures 5A, B**). Based on three independent prognostic parameters including risk score, age, and tumor stage (*p* < 0.01 in multi-Cox), a nomogram was constructed to forecast the incidences of OS at 1, 3 and 5 years in BLCA patients (**Figure 5C**). The 1-, 3-, and 5-year calibration plots were used to demonstrate that the nomogram was in good agreement with the prediction of OS at 1, 3 and 5 years (**Figure 5D**).

### Evaluation of Risk Models

**Figure 6:**
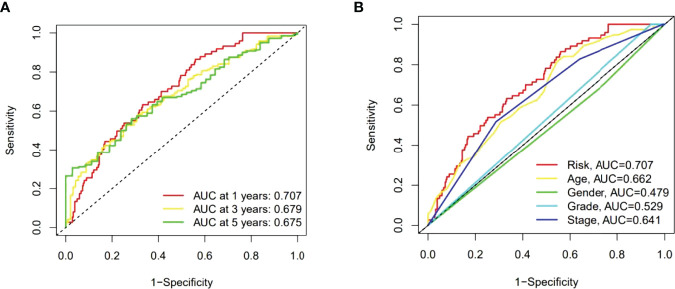
The 1-, 3-, and 5-year ROC curves of the whole group **(A)**. The 5-year ROC curves of the risk score and clinical factors **(B)**.

Then the sensitivity and specificity of the constructed model was assessed for prognosis *via* time-dependent receiver operating characteristics (ROC). The acreages under the ROC curve (AUC) were used to exemplify ROC results. The 1-, 3-, and 5-year AUC of the whole group were 0.707, 0.679, and 0.675 (**Figures 6A**). In the above ROC of risk model, risk score (0.707) showed primary predictive power compared other clinical indicators including age (0.662), gender (0.479), grade (0.529) and stage (0.641) (**Figure 6B**).

### GSEA

**Figure 7:**
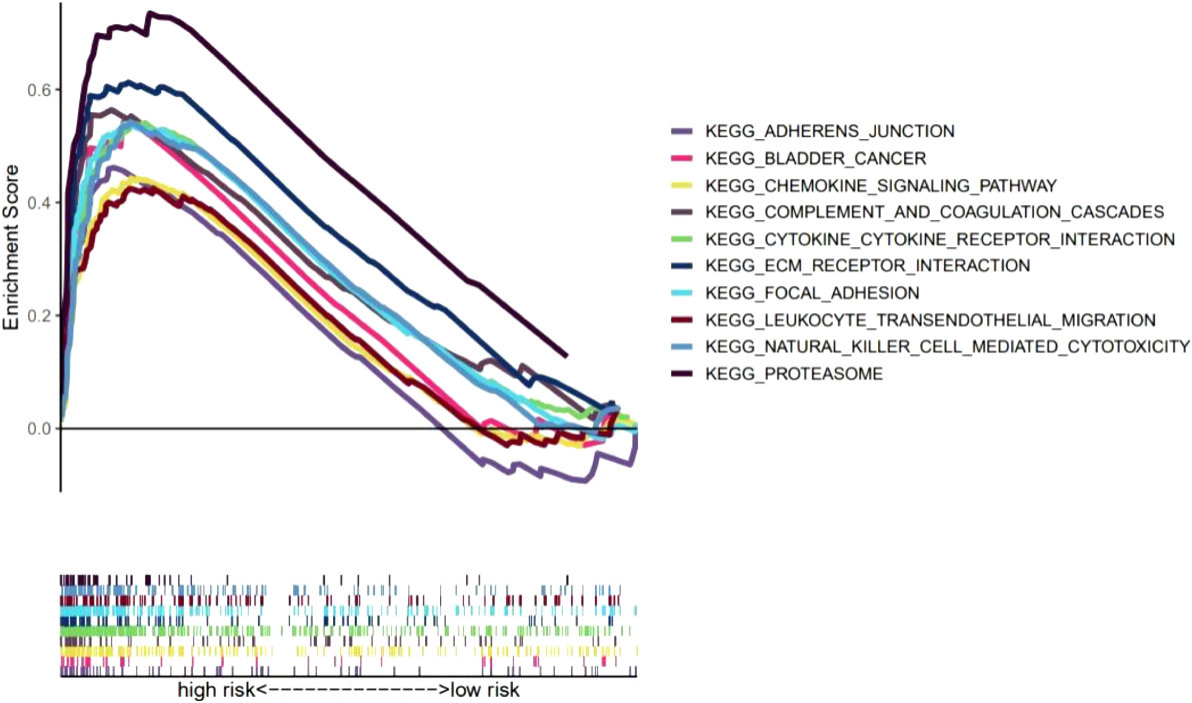
GSEA of the 10 pathways significantly enriched in the high-risk group.

Our team also explored the KEGG pathways in the high-risk group of TCGA-BLCA cohort using GSEA software to explore discrepancies in biological functions between risk groups (**Supplementary Figure S4**). The pathways enriched in the high-risk group revealed biological functions highly correlated with tumor invasion and immune function, such as complement and coagulation cascades and leukocyte transendothelial migration (p<0.05) (**Figure 7**). Therefore, the subsequent immune correlation analysis should be explored in our model.

### The Exploration of Immunological Factors and Clinical Treatment in Risk Groups

**Figure 8:**
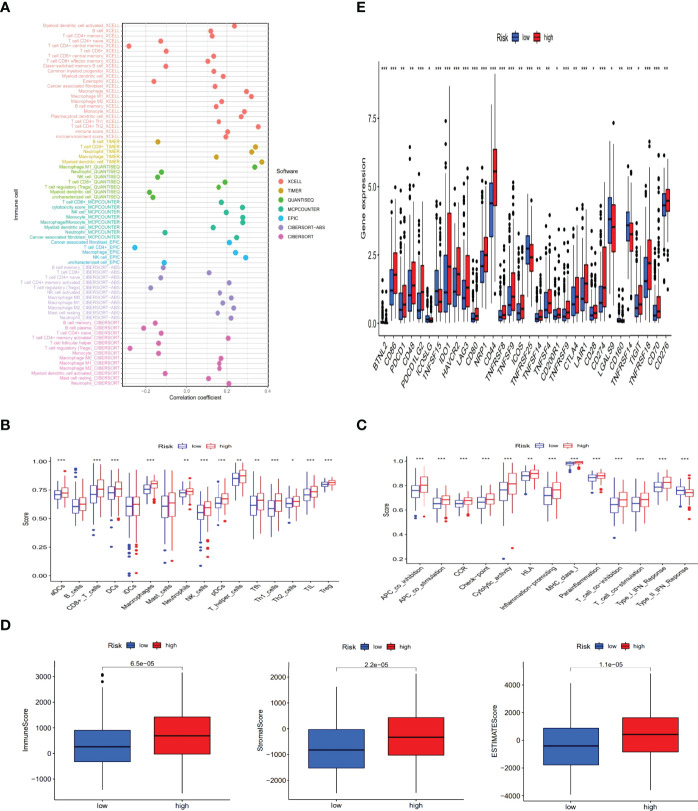
The exploration of tumor immune characteristics and immunotherapy. The immune cell bubble of risk groups **(A)**. Differences of immune cells and immune function between low- and high-risk groups **(B, C)**. Comparison of StromalScore, ImmuneScore, and ESTIMATEScore between low and high risk groups **(D)**. Distinctions of 32 immune checkpoints gene expression in risk groups **(E)**. (*: p < 0.05, **: p < 0.01, and ***: p < 0.001).

To further research the correlation between the prognosis of risk groups and immune infiltration status, we performed the analysis on different immune filtration platforms. Although the algorithms were different for each platform and the immune data are not completely identical for the high and low risk groups, we can conclude that a large number of immune cells were concentrated in the high risk group, such as B cells, macrophage in TIMER, T cell CD4+ naive, T cell CD8 + in XCELL, T cell CD8+, myeloid dendritic cell in MCPCOUNTER, T cell CD8+, Neutrophil in QUANTISEQ and T cell CD4 + in EPIC (all p < 0.05) (**Figure 8A** and **Supplementary Appendix T3**). In addition, ssGSEA scores of major immune cells and immune function were also compared between the high- and low-risk groups (**Figures 8B, C**). All of above indicated a higher immune infiltration status and more immune functions in the high-risk group. The high-risk group also showed a higher ESTIMAT (microenvironment) score and an immune score, revealing that the high-risk group had a higher overall immune level and immunogenicity of TME than the low-risk group (**Figure 8D**). Additionally, most immune checkpoints were markedly up-regulated in the high-risk group (**Figure 8E**). This suggested that we could select proper checkpoint blockers for BLCA patients in high-risk group. Based on the gene expression levels in our risk model, we also screened 47 chemical or targeted drugs, which had lower IC50 values in the high-risk group with a higher immune score than low-risk group (**Supplementary Figure S1**).

### Precision Medicine for Tumor Staging and Clustering

**Figure 9:**
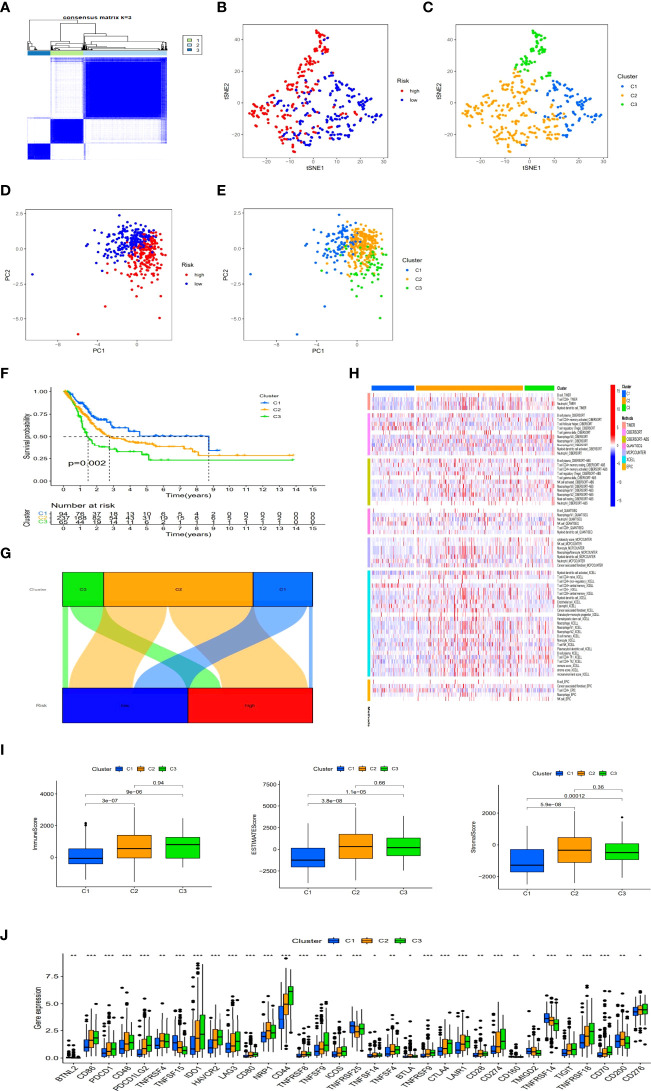
Immunotherapy prediction and differences between cold and hot tumors. Patients regrouped three clusters *via* ConsensusClusterPlus **(A)**. The t-SNE of three clusters and risk groups **(B, C)**. The PCA of risk groups and clusters **(D, E)**. Clustered Kaplan–Meier survival curves for OS **(F)**. The connection between risk groups and clusters **(G)**. Clusters of immunological cells on a heat map **(H)**. Comparison of immune-related scores among three clusters **(I)**. The distinction of 32 immune checkpoints expression in clusters **(J)**. (*: p < 0.05, **: p < 0.01, and ***: p < 0.001).

Previous studies have shown that there were different subtypes of tumors with a diverse immune microenvironment, which caused different immunotherapeutic reactions (19). According to necroptosis-related lncRNAs, patients could be divided into three clusters utilizing the Consensus ClusterPlus R package (**Figure 9A** and **Supplementary Figure S2**). T-distributed stochastic neighbor embedding (t-SNE) suggested three clusters and risk groups could be identified clearly (**Figures 9B, C**). Additionally, we used principal component analyses (PCA) to confirm that different PCA exited in both risk groups and clusters (**Figures 9D, E**). Furthermore, Kaplan–Meier analysis shown cluster 1 had better OS and cluster3 had worse OS (p=0.002) (**Figure 9F**). A graph was also made in order to understand its correlation with risk. Cluster 1 was significantly related with the low-risk group, and cluster 2 and cluster3 were related with the high-risk group (**Figure 9G**). According to the analysis of the different platforms, cluster 2 and 3 were more infiltrated by immune cells (**Figure 9H**) (**Supplementary Appendix T4**). Cluster 2 and 3 also had a higher immune score and a higher ESTIMAT (microenvironment) score, indicating a different TME than cluster 1 (**Figure 9I**). Most of the immune checkpoints in clusters 2 and 3 expressed higher activity, such as CD274 (PD-L1), LAG3 and HAVCR2(TIM-3) (**Figure 9J**). The above results suggested that immunotherapy could be helpful for patients in cluster 2 and cluster3 of the risk group. Previous studies have shown that infiltration of multiple immune cells, activation of CD274 (PD-L1), LAG3 and HAVCR2(TIM-3) and high immune scores played a crucial role in hot tumor (20). Consequently, we could regard cluster 2 and 3 as the hot tumor and cluster1 as the cold tumor. This classification might lead to different immunotherapeutic responses. Under the concept of cold and hot tumors, immunotherapy has a more active role in cluster 2 and 3. By comparing drug sensitivities, we found that multiple drugs including immunotherapeutic drugs as well as chemotherapeutic or targeted drugs exhibited different IC50 in BLCA clusters (**Supplementary Figure S3**). The above results may further help us to improve the research of immunotherapies and the appropriate application of patients with BLCA.

## Discussion

Immunotherapy related with immune checkpoint inhibitors (ICI) has led to a significant improvement in the clinical treatment of malignancy. However, the efficacy is poor for tumors with immunosuppressive TME. We found that infiltration of T cells is a prerequisite for ICI-mediated antitumor responses. CD8+ T cells can kill cancer cells by releasing GNLY or GZM and PRF1 and enhance immunotherapy through the PD-1/PD-L1 immunosuppressive axis, helping to break immune tolerance. Consequently, ICI is inadequate against “cold tumors” that lack T-cell infiltration. Hot tumors are generally characterized by high T-cell infiltration and a high immune score. Therefore, relevant immunotherapy has achieved good results in the clinical treatment of hot tumors. To ensure clinical treatment of immunotherapy, it is vital to differentiate between cold and hot tumors and turn cold tumors into hot tumors (20, 21).

In our work, we regrouped patients into low- and high-risk groups through the model of 7 lncRNAs associated with necroptosis *via* Lasso regression when the first-rank value of Log(*λ*) was the least deviation possibility and performed several assessments such as Kaplan-Meier analysis, prognostic analysis, GSEA and IC50 prediction. In 1-, 3-, and 5-year ROC curves, the area under the ROC curve was all higher than 0.65. In ROC curves of risk scores and traditional clinical factors, the area of risk scores under the ROC curve was higher than our conventional clinical indicators. Compared with current models, such as model of N6-methylandenosine-related lncRNAs, our model predicts 1, 3, and 5-year survival rates with higher accuracy than N6-methylandenosine-related lncRNAs model (22). All of these results showed the rationality and superiority of our 7 lncRNAs model. Although risk groups were expected to be of great value for tumor prognosis and systemic therapy, we were unable to identify hot and cold tumors. Different molecular subtypes, also known as clusters, have been reported to be associated with diverse immune status and TME, which caused different prognosis and immunotherapeutic response (19). And lncRNAs have been identified as a tumor biomarker in previous studies (15, 16). Therefore, we classified patients into three clusters based on the expression of these lncRNAs. We found that the three clusters had different immune microenvironments. Clusters 2 and 3 had more highly infiltrated CD8 T cells, higher immune scores and higher activity of CD274, LAG3 and HAVCR2 compared to cluster 1 with immunosuppressive TME, which could be regarded as hot tumors (20, 21). More importantly, clusters 2 and 3 were more sensitive to immunotherapeutic drugs. In conclusion, necroptosis-associated lncRNAs not only can predict the prognosis and contribute to individual therapy, but also can swiftly and readily distinguish between cold and hot tumors (23, 24).

We also found some of these lncRNAs associated with known genes including ATRX, BRAF, GATA3, OUTLIN, and TSCL in the Sankey diagram. ATRX can promote immunogenicity and anti-cancer immunity by affecting DNA damage repair pathways (25). The BRAF signaling pathway can induce immunosuppressive factors to evade the immune system. The immunosuppressive factors can be reduced by blocking BRAF signaling to further improve the tumor microenvironment and make it more prone to T cell infiltration (26). GATA3 has been reported to be a major regulator of TH2 cells, contributing to the efficient transcription of encoded cytokines by TH2 cells such as IL-4, IL-5, and IL-13 (27). The GATA3-AS1 lncRNA promotes tumor progression and immune evasion by stabilizing PD-L1 protein and degrading GATA3 protein (14). This result may provide new targets for the treatment of BLCA. OUTLIN is thought to influence the level of ubiquitination. The lack of OUTLIN will lead to NF-κB activation and the production of excessive pro-inflammatory cytokines (28). TSCL plays an important role in the regulation of peripheral naïve CD8+ T cell survival and homeostasis *in vivo* (29). The necrosis-associated lncRNAs we got can regulate the tumor microenvironment associated with immunity and inflammation, which helps us to have a better mechanistic understanding of BLCA and bring breakthroughs into subsequent clinical practice.

Our study also has some deficiencies. First, as a retrospective study, it is susceptible to its own inherent bias (30). Second, we obtained our lncRNA risk signature from the public dataset TCGA. On the other hand, we searched many databases, such as GSE13507 in the GEO database. But we could not get appropriate information of lncRNAs. We suspected that commercial microarray data had biases and restrictions when compared to TCGA data. However, the data for our immune cell bubble map come from multiple platforms, which could be used as a validation of the external data. In our study, the ratio of tumor samples to normal samples was unbalanced (414 vs 19), which may have influenced the precision of the functional enrichment analysis. Besides, the molecular mechanisms of necroptosis-associated lncRNAs included in our signature are still unknown, and subsequent basic experiments are needed to explore specific molecular mechanisms. Encouragingly, only tumor samples with available clinical data were used in univariate and multivariate analyses for constructing necroptosis-associated lncRNA risk signatures, so the limited number of normal samples had no effect on the accuracy and reliability of our constructed model, and our model is amenable to future clinical testing.

In conclusion, we constructed a novel necroptosis-related lncRNA signature with good specificity and sensitivity for predicting survival prognosis and response to immunotherapy in patients with BLCA. Importantly, hot and cold tumors could be discriminated on the basis of these lncRNAs to further achieve precision medicine.

## Conclusions

Necrosis-associated lncRNAs could predict survival prognosis and help identify cold and hot tumors to further suggest effective therapeutic strategies, resulting in great advances in individual therapy and patient prognosis. Therefore, we could overcome systemic therapeutic failures and improve the efficacy of immunotherapy by targeting necroptosis-associated lncRNAs. As a result, the regulatory mechanism network among immunity, necroptosis, lncRNAs in BLCA deserve to be fully clarified and validated.

## Data Availability Statement

The datasets presented in this study can be found in online repositories. The names of the repository/repositories and accession number(s) can be found in the article/**Supplementary Material**.

## Author Contributions

HZ, DL, SX, TC, and SM studied and designed all bioinformatics analysis. DL, KW, GS, and SC critically reviewed our manuscript. Administrative, technical, and material support were provided by DL and SX. DL and SX contributed equally to our research. All authors contributed to the article and approved the submitted version.

## Funding

Our study was supported by the grants of National Natural Science Foundation of China (81972412 and 81772758).

## Conflict of Interest

The authors declare that the research was conducted in the absence of any commercial or financial relationships that could be construed as a potential conflict of interest.

## Publisher’s Note

All claims expressed in this article are solely those of the authors and do not necessarily represent those of their affiliated organizations, or those of the publisher, the editors and the reviewers. Any product that may be evaluated in this article, or claim that may be made by its manufacturer, is not guaranteed or endorsed by the publisher.

## Supplementary Material

Appendix T1 67 genes associated with necroptosis disease

Appendix T2 The network information of necroptosis-associated genes and lncRNAs.

Appendix T3 The results of the immune cell correlation analysis.

Appendix T4 the data of significantly different infiltrated immune cells among clusters on various platforms.

Supplementary Figure 1 The potential drug forecast for risk groups.

Supplementary Figure 2 Estimation of consensus clustering of necroptosis-associated lncRNAs.

Supplementary Figure 3 Drug sensitivity analysis among clusters.

Supplementary Figure 4 The GSEA of the high-risk group.

## References

[1] Rose TL Milowsky MI. Improving Systemic Chemotherapy for Bladder Cancer. Curr Oncol Rep (2016) 18:27. doi: 10.1007/s11912-016-0512-2 26984414

[2] Siegel RL Miller KD Jemal A. Cancer Statistics, 2019. CA Cancer J Clin (2019) 69(1):7–34. doi: 10.3322/caac.21551 30620402

[3] Bray F Ferlay J Soerjomataram I Siegel RL Torre LA Jemal A. Global Cancer Statistics 2018: GLOBOCAN Estimates of Incidence and Mortality Worldwide for 36 Cancers in 185 Countries. CA Cancer J Clin (2018) 68(6):394–424. doi: 10.3322/caac.21492 30207593

[4] Antoni S Ferlay J Soerjomataram I Znaor A Jemal A Bray F. Bladder Cancer Incidence and Mortality: A Global Overview and Recent Trends. Eur Urol 71(1):96–108. doi: 10.1016/j.eururo.2016.06.010 27370177

[5] Darvin P Toor SM Sasidharan Nair V Elkord E. Immune Checkpoint Inhibitors: Recent Progress and Potential Biomarkers. Exp Mol Med (2018) 50(12):1–11. doi: 10.1038/s12276-018-0191-1 PMC6292890 30546008

[6] Bonaventura P Shekarian T Alcazer V Valladeau-Guilemond J Valsesia-Wittmann S Amigorena S. Cold Tumors: A Therapeutic Challenge for Immunotherapy. Front Immunol (2019) 10:168. doi: 10.3389/fimmu.2019.00168 30800125 PMC6376112

[7] Galluzzi L Vitale I Abrams JM Alnemri ES Baehrecke EH Blagosklonny MV. Molecular Definitions of Cell Death Subroutines: Recommendations of the Nomenclature Committee on Cell Death 2012. Cell Death Differ (2012) 19(1):107–20. doi: 10.1038/cdd.2011.96 PMC3252826 21760595

[8] Xin S Mao J Duan C Wang J Lu Y Yang J. Identification and Quantification of Necroptosis Landscape on Therapy and Prognosis in Kidney Renal Clear Cell Carcinoma. Front Genet (2022) 13:832046. doi: 10.3389/fgene.2022.832046 35237304 PMC8882778

[9] Tang R Xu J Zhang B Liu J Liang C Hua J. Ferroptosis, Necroptosis, and Pyroptosis in Anticancer Immunity. J Hematol Oncol (2020) 13(1):110. doi: 10.1186/s13045-020-00946-7 32778143 PMC7418434

[10] Aaes TL Kaczmarek A Delvaeye T De Craene B De Koker S Heyndrickx L. Vaccination With Necroptotic Cancer Cells Induces Efficient Anti-Tumor Immunity. Cell Rep (2016) 15(2):274–87. doi: 10.1016/j.celrep.2016.03.037 27050509

[11] Su Y Wu H Pavlosky A Zou L-L Deng X Zhang Z-X. Regulatory non-Coding RNA: New Instruments in the Orchestration of Cell Death. Cell Death Dis (2016) 7(8):e2333. doi: 10.1038/cddis.2016.210 27512954 PMC5108314

[12] Min W Sun L Li B Gao X Zhang S Zhao Y. LncCRLA Enhanced Chemoresistance in Lung Adenocarcinoma That Underwent EpithelialMesenchymal Transition. Oncol Res (2022) 28(9):857–72. doi: 10.3727/096504021X16203818567367 PMC8790133 33985619

[13] Tran DDH Kessler C Niehus SE Mahnkopf M Koch A Tamura T. Myc Target Gene, Long Intergenic Noncoding RNA, Linc00176 in Hepatocellular Carcinoma Regulates Cell Cycle and Cell Survival by Titrating Tumor Suppressor microRNAs. Oncogene (2018) 37(1):75–85. doi: 10.1038/onc.2017.312 28869604

[14] Zhang M Wang N Song P Fu Y Ren Y Li Z. LncRNA GATA3-AS1 Facilitates Tumour Progression and Immune Escape in Triple-Negative Breast Cancer Through Destabilization of GATA3 But Stabilization of PD-L1. Cell Prolif (2020) 53(9):e12855. doi: 10.1111/cpr.12855 32687248 PMC7507373

[15] Wang L Cho KB Li Y Tao G Xie Z Guo B. Long Noncoding RNA (lncRNA)-Mediated Competing Endogenous RNA Networks Provide Novel Potential Biomarkers and Therapeutic Targets for Colorectal Cancer. Int J Mol Sci (2019) 20(22):5758. doi: 10.3390/ijms20225758 PMC6888455 31744051

[16] Liang X Zha L Yu G Guo X Qin C Cheng A. Construction and Comprehensive Prognostic Analysis of a Novel Immune-Related lncRNA Signature and Immune Landscape in Gastric Cancer. Int J Genomics (2022) 2022:4105280. doi: 10.1155/2022/4105280 35083327 PMC8786486

[17] Geeleher P Cox NJ Huang RS. Clinical Drug Response can be Predicted Using Baseline Gene Expression Levels and *In Vitro* Drug Sensitivity in Cell Lines. Genome Biol (2014) 15(3):R47. doi: 10.1186/gb-2014-15-3-r47 24580837 PMC4054092

[18] Geeleher P Cox N Huang RS. Prrophetic: An R Package for Prediction of Clinical Chemotherapeutic Response From Tumor Gene Expression Levels. PLos One (2014) 9(9):e107468. doi: 10.1371/journal.pone.0107468 25229481 PMC4167990

[19] Becht E de Reyniès Aurélien Giraldo NA Pilati C Buttard Bénédicte Lacroix L. Immune and Stromal Classification of Colorectal Cancer Is Associated With Molecular Subtypes and Relevant for Precision Immunotherapy. Clin Cancer Res (2016) 22(16):4057–66. doi: 10.1158/1078-0432.CCR-15-2879 26994146

[20] Zhao Z Liu H Zhou X Fang D Ou X Ye J. Necroptosis-Related lncRNAs: Predicting Prognosis and the Distinction Between the Cold and Hot Tumors in Gastric Cancer. J Oncol (2021) 2021:6718443. doi: 10.1155/2021/6718443 34790235 PMC8592775

[21] Liu YT Sun ZJ. Turning Cold Tumors Into Hot Tumors by Improving T-Cell Infiltration. Theranostics (2021) 11(11):5365–86. doi: 10.7150/thno.58390 PMC8039952 33859752

[22] Zhang Y Zhu B He M Cai Y Ying X Jiang C. N6-Methylandenosine-Related lncRNAs Predict Prognosis and Immunotherapy Response in Bladder Cancer. Front Oncol (2021) 11:710767. doi: 10.3389/fonc.2021.710767 34458149 PMC8387102

[23] Sun J Zhang Z Bao S Yan C Hou P Wu N. Identification of Tumor Immune Infiltration-Associated lncRNAs for Improving Prognosis and Immunotherapy Response Ofpatients With Non-Small Cell Lung Cancer. J Immunother Canc (2020) 8(1):e000110. doi: 10.1136/jitc-2019-000110 PMC7057423 32041817

[24] Duan Q Zhang H Zheng J Zhang L. Turning Cold Into Hot: Firing Up the Tumor Microenvironment. Trends Canc (2020) 6(7):605–18. doi: 10.1016/j.trecan.2020.02.022 32610070

[25] Ge Y Wei F Du G Fei G Li W Li X. The Association of Sex-Biased ATRX Mutation in Female Gastric Cancer Patients With Enhanced Immunotherapy-Related Anticancer Immunity. BMC Canc (2021) 21(1):240. doi: 10.1186/s12885-021-07978-3 PMC7938533 33678158

[26] Hu-Lieskovan S Robert L Homet Moreno B Ribas A. Combining Targeted Therapy With Immunotherapy in BRAF-Mutant Melanoma: Promise and Challenges. J Clin Oncol (2014) 32(21):2248–54. doi: 10.1200/JCO.2013.52.1377 PMC4164812 24958825

[27] Gibbons HR Shaginurova G Kim LC Chapman N Spurlock CF 3rd Aune TM. Divergent lncRNA GATA3-AS1 Regulates GATA3 Transcription in T-Helper 2 Cells. Front Immunol (2018) 9:2512. doi: 10.3389/fimmu.2018.02512 30420860 PMC6215836

[28] Kone-Paut I Georgin-Laviallec S Galeotti C Rossi-Semerano L Hentgen V Savey L. New Data in Causes of Autoinflammatory Diseases. Joint Bone Spine (2019) 86(5):554–61. doi: 10.1016/j.jbspin.2018.11.003 30471422

[29] Zhang L Zhang H Li L Xiao Y Rao E Miao Z. TSC1/2 Signaling Complex is Essential for Peripheral Naïve CD8+ T Cell Survival and Homeostasis in Mice. PLos One (2012) 7(2):e30592. doi: 10.1371/journal.pone.0030592 22363451 PMC3283604

[30] Jiang Y-Z Liu Y-R Xu X-E Jin Xi Hu X Yu K-D. Transcriptome Analysis of Triple-Negative Breast Cancer Reveals an Integrated mRNA-lncRNA Signature With Predictive and Prognostic Value. Cancer Res (2016) 76(8):2105–14. doi: 10.1158/0008-5472.CAN-15-3284 26921339

