# Supplementary material for: Predicting Prognosis and Distinguishing Cold and Hot Tumors in Bladder Urothelial Carcinoma Based on Necroptosis-Associated lncRNAs
Source: Front Immunol. 2022 Jul 4;13:916800. doi: 10.3389/fimmu.2022.916800 (PMC9289196; doi:10.3389/fimmu.2022.916800)
Supplement: Supplementary Figure 1 — The potential drug forecast for risk groups. [file DataSheet_1.pdf]

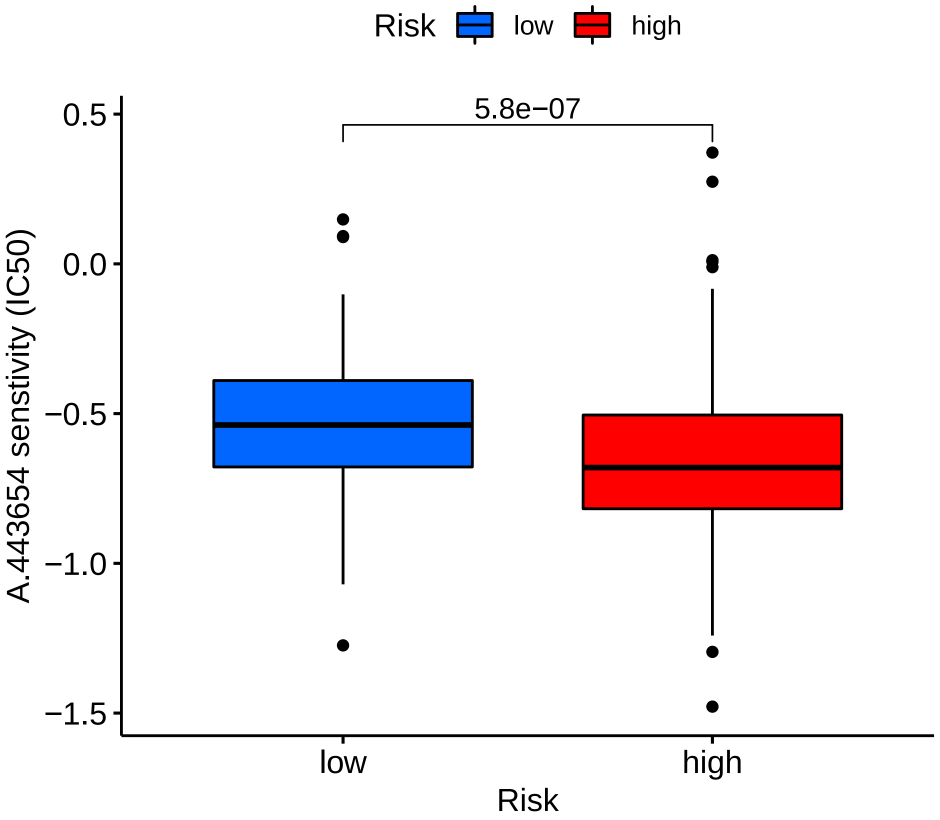

Risk
low
high
5.8e−07
0.5
0.0
−0.5
−1.0
−1.5
A.443654 senstivity (IC50)
low
high
Risk

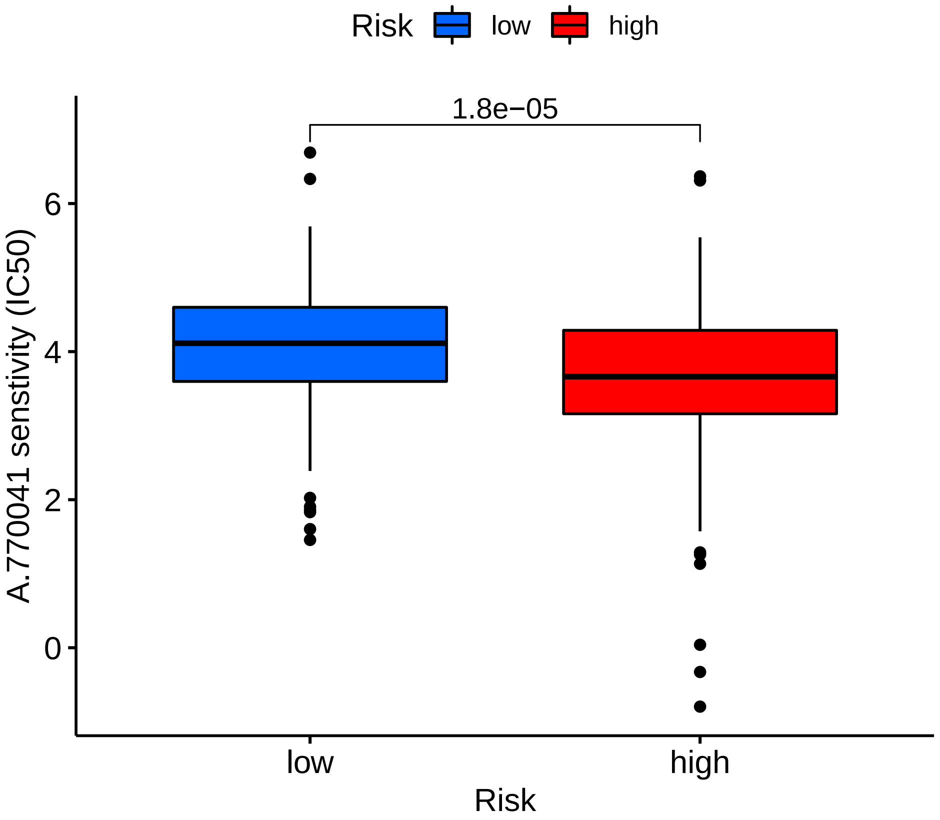
Risk
low
high
1.8e−05
A.770041 senstivity (IC50)
6
4
2
0
low
high
Risk

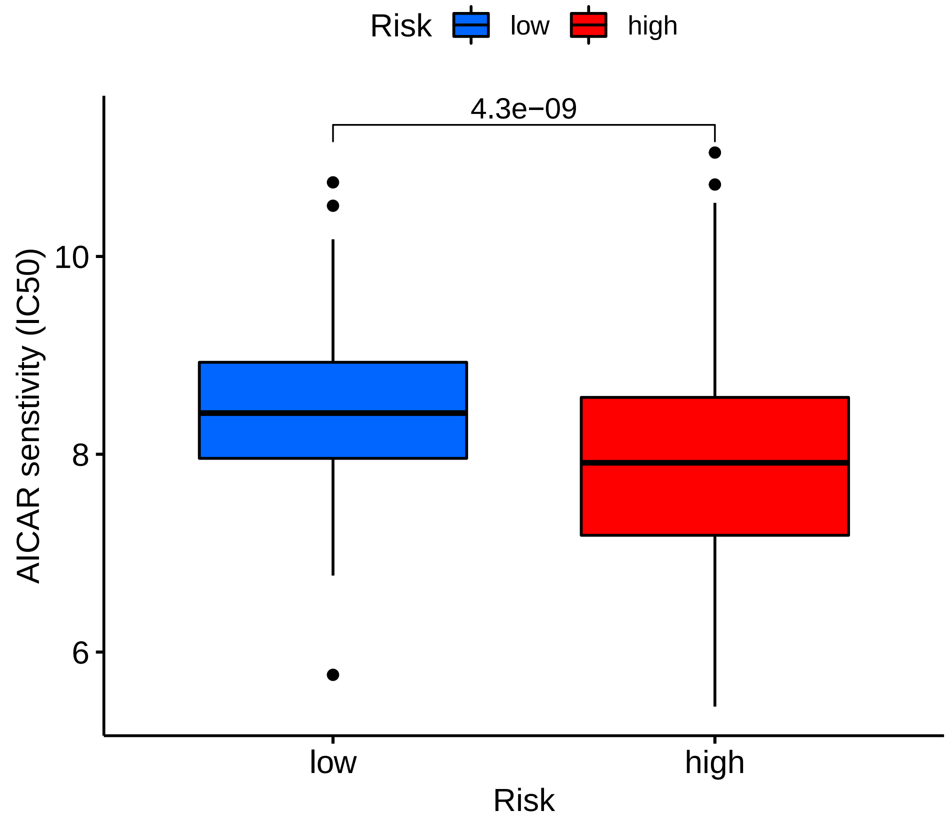
Risk
low
high
4.3e−09
AICAR senstivity (IC50)
10
8
6
low
high
Risk

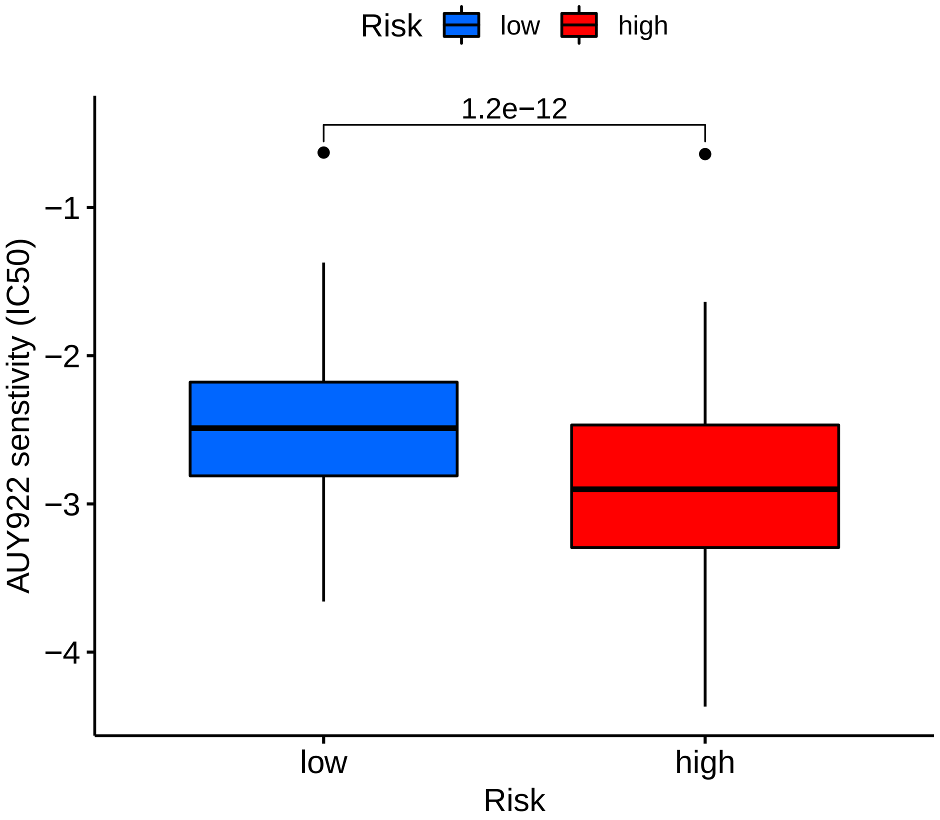
Risk
low
high
1.2e−12
−1
−2
−3
−4
AUY922 senstivity (IC50)
low
high
Risk

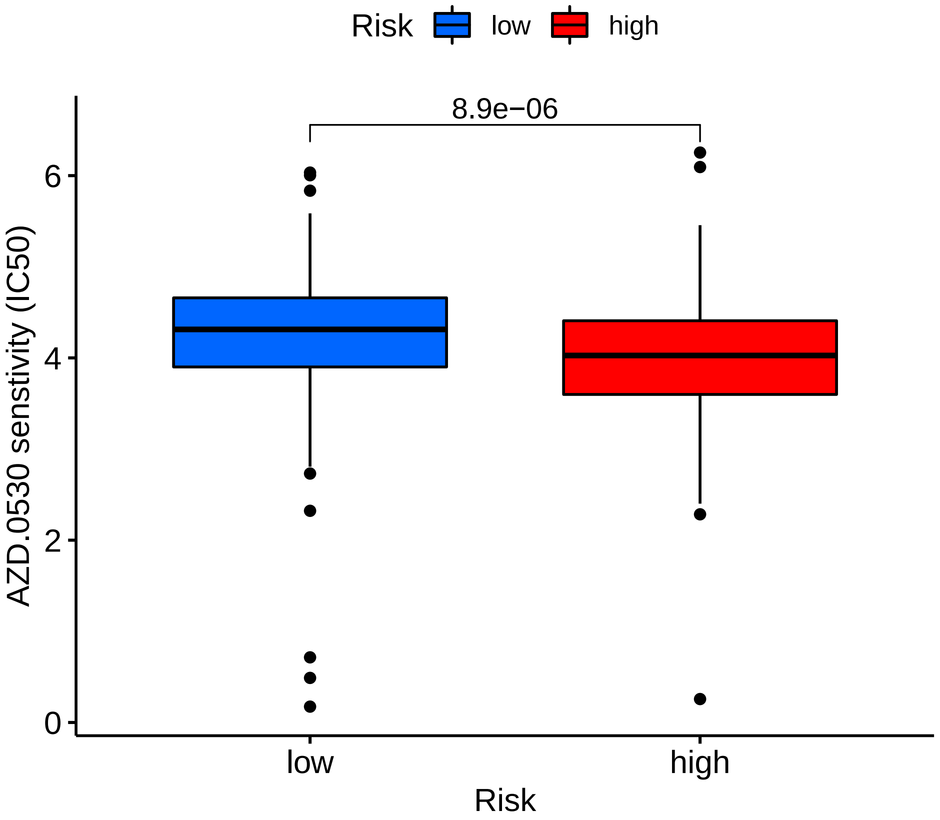
Risk
low
high
8.9e−06
AZD.0530 senstivity (IC50)
6
4
2
0
low
high
Risk

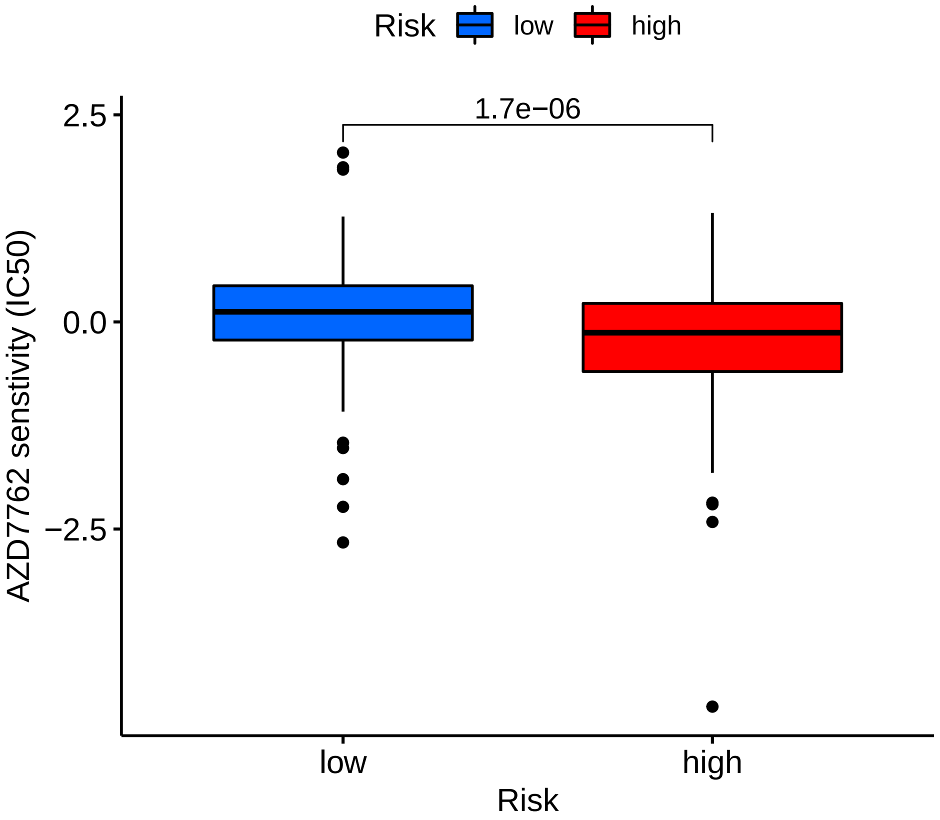
Risk
low
high
1.7e−06
2.5
0.0
−2.5
AZD7762 senstivity (IC50)
low
high
Risk

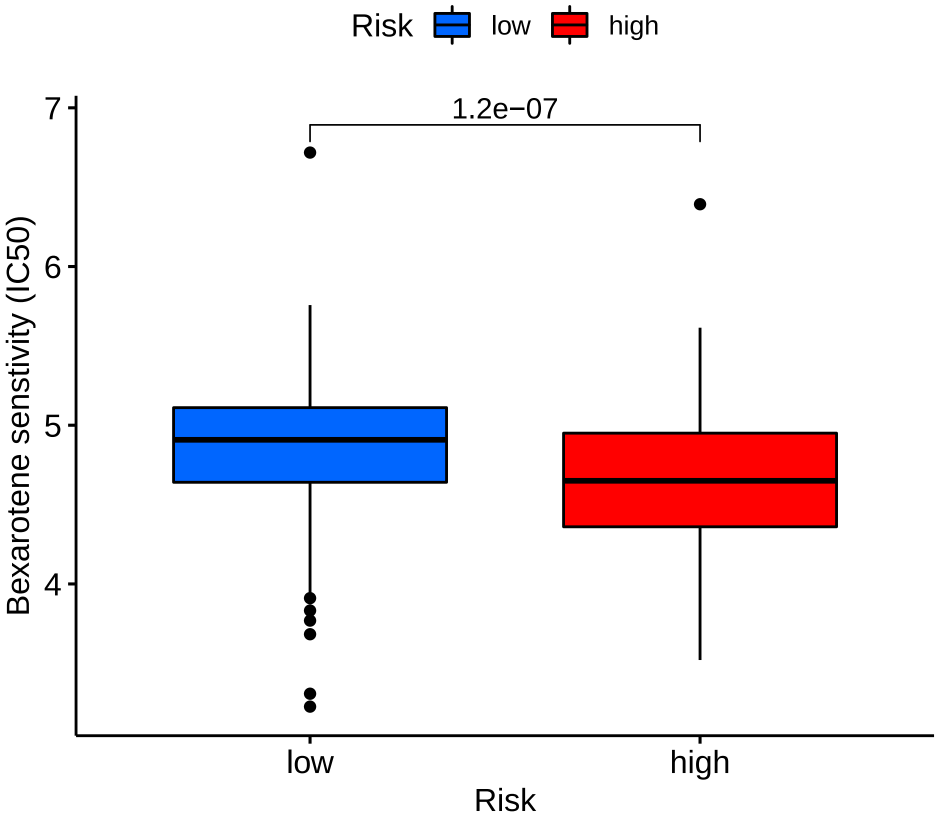
Risk
low
high
1.2e−07
Bexarotene senstivity (IC50)
7
6
5
4
low
high
Risk

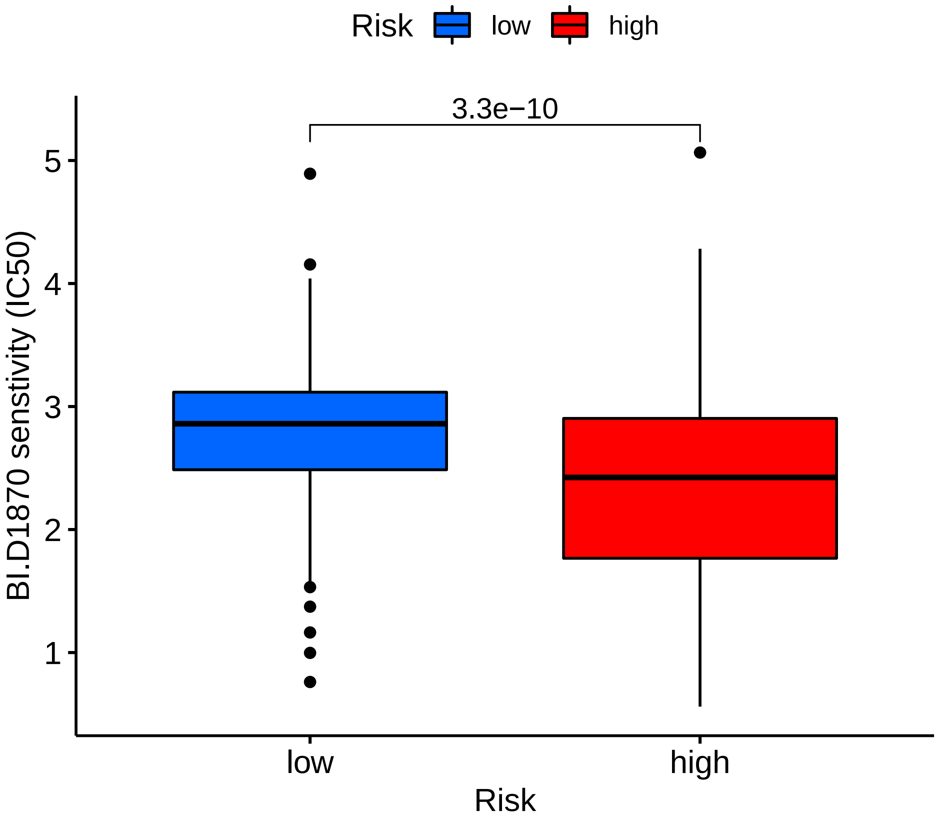

Risk
low
high
3.3e−10
5
4
3
2
1
BI.D1870 senstivity (IC50)
low
high
Risk

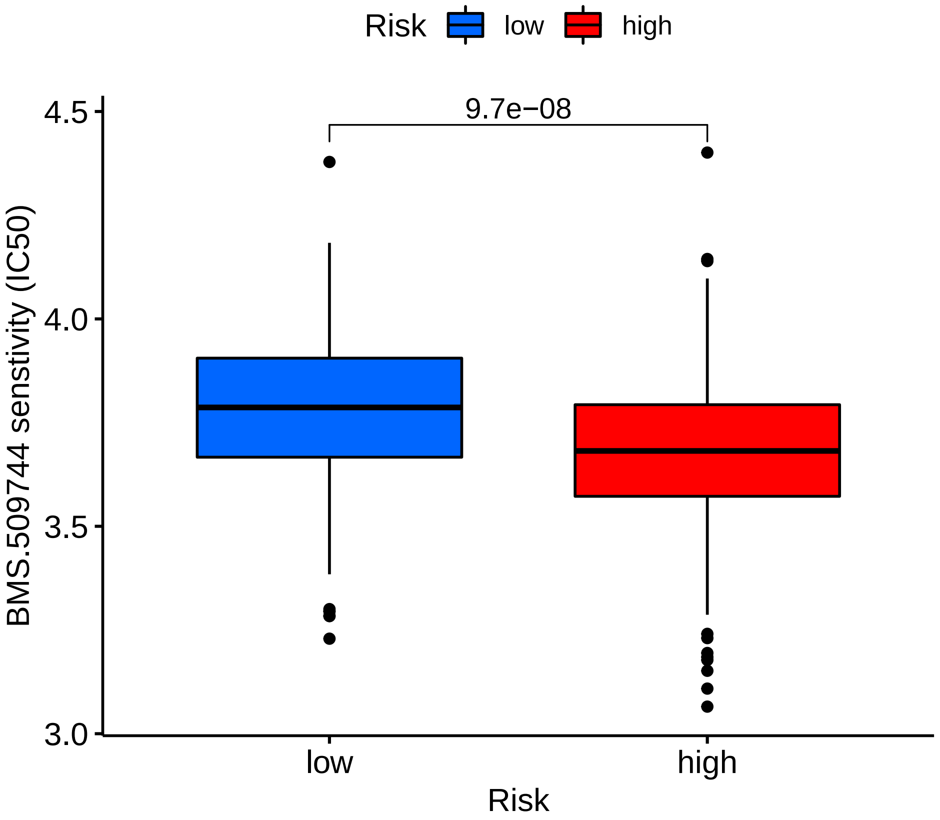
Risk
low
high
9.7e−08
4.5
4.0
3.5
3.0
BMS.509744 senstivity (IC50)
low
high
Risk

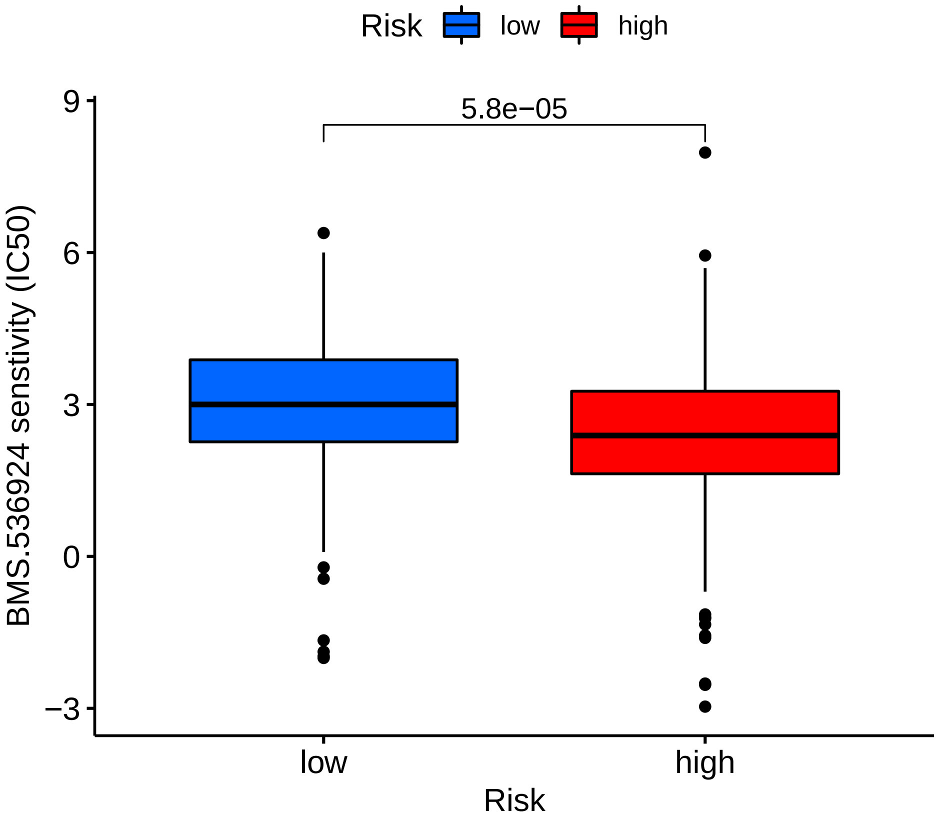

Risk
low
high
5.8e−05
BMS.536924 senstivity (IC50)
9
6
3
0
−3
low
high
Risk

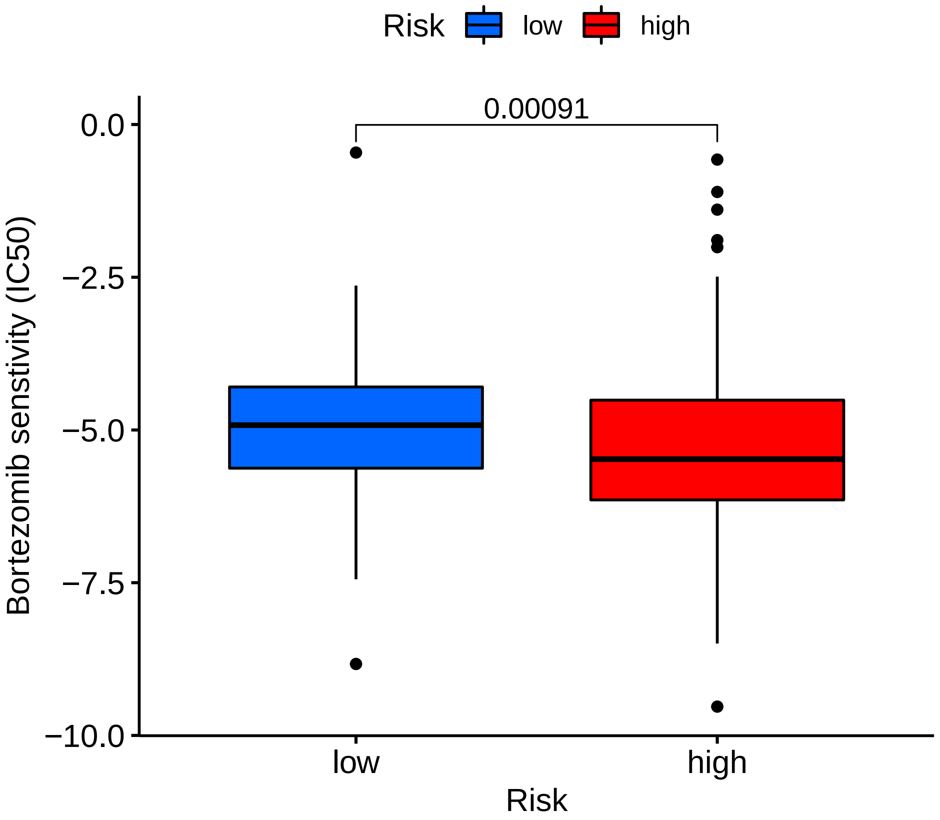

Risk
low
high
0.00091
0.0
−2.5
−5.0
−7.5
−10.0
Bortezomib senstivity (IC50)
low
high
Risk

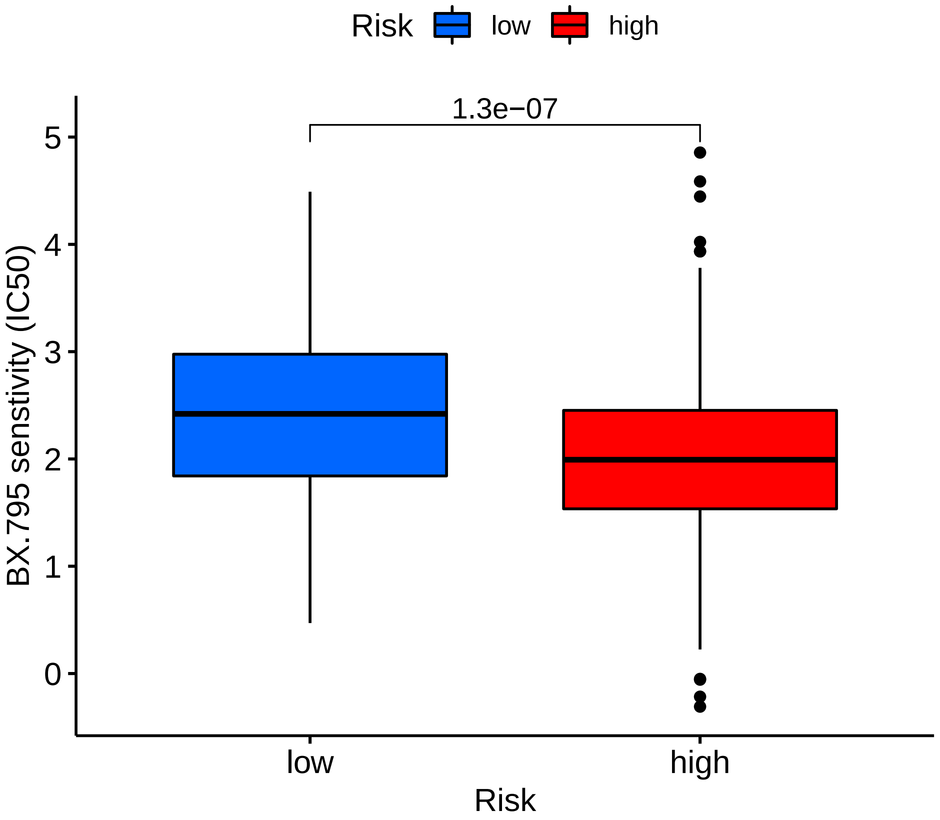

Risk
low
high
1.3e−07
BX.795 senstivity (IC50)
5
4
3
2
1
0
low
high
Risk

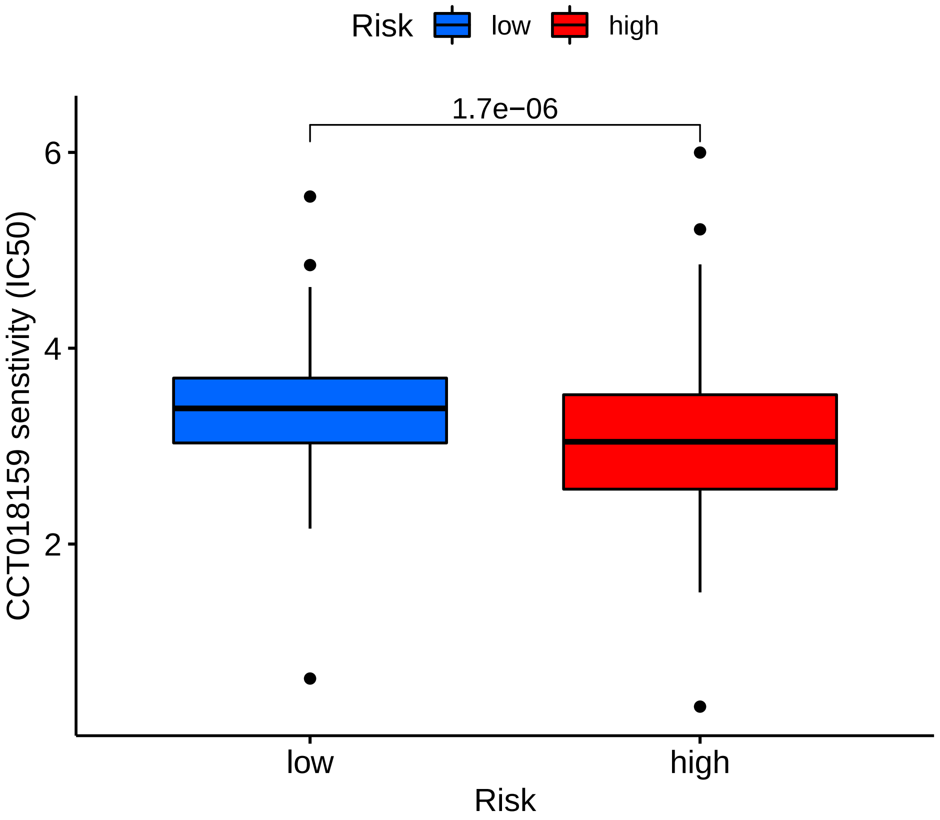
Risk
low
high
1.7e−06
CCT018159 senstivity (IC50)
6
4
2
low
high
Risk

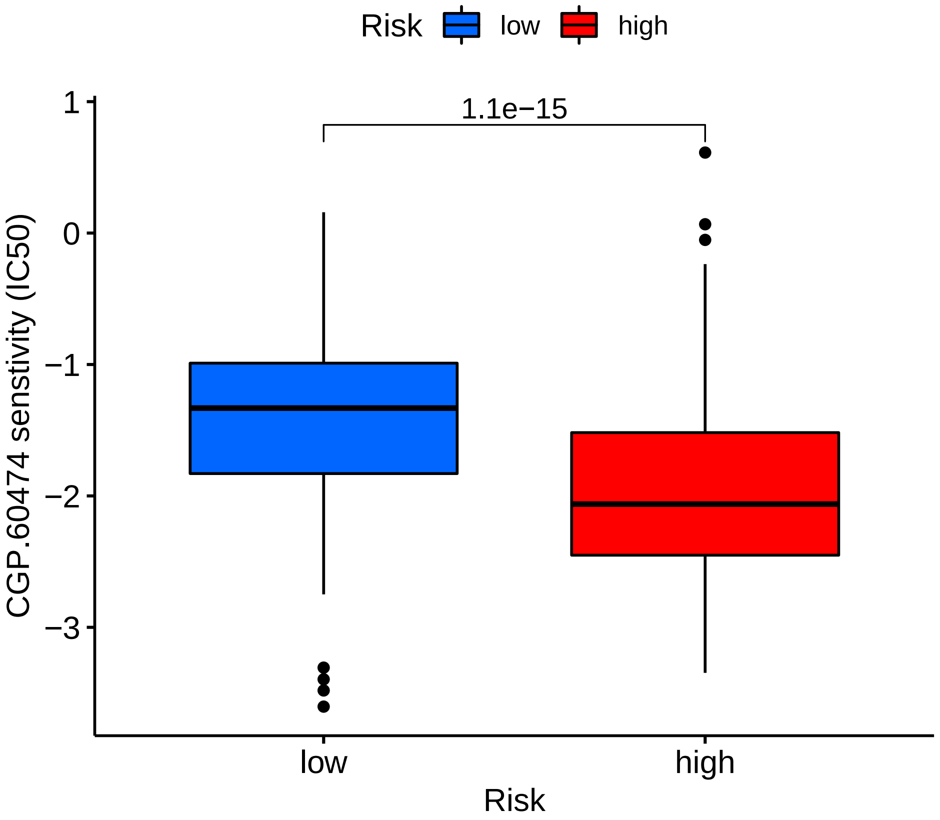
Risk
low
high
1.1e−15
1
0
−1
−2
−3
CGP.60474 senstivity (IC50)
low
high
Risk

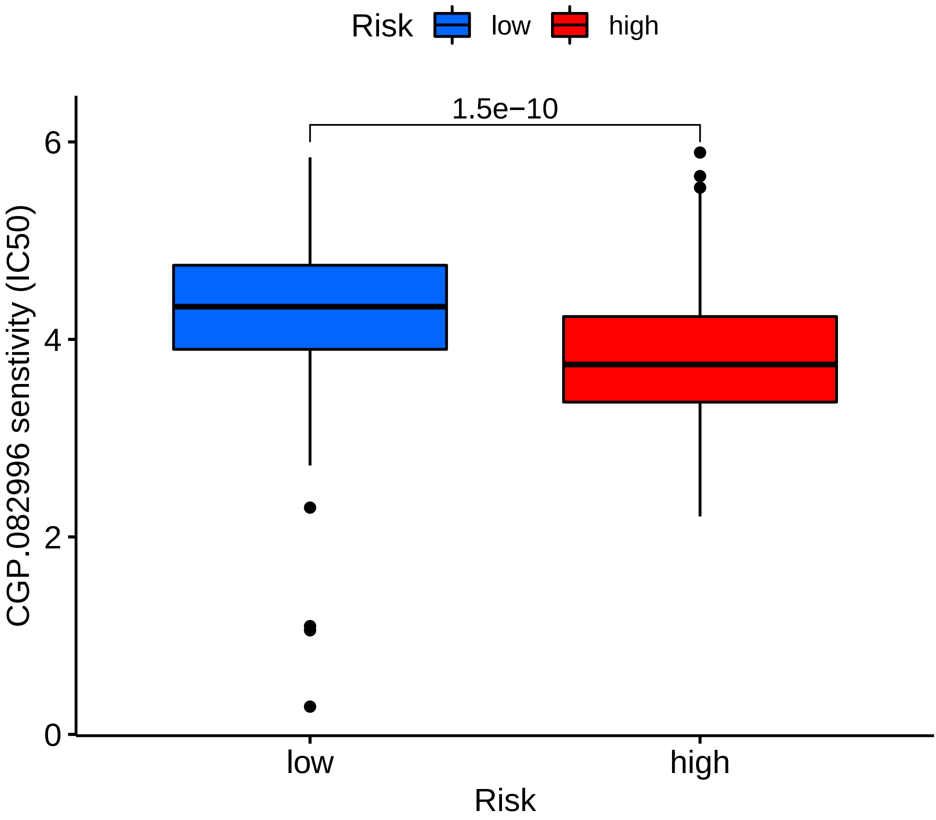
Risk
low
high
1.5e−10
CGP.082996 senstivity (IC50)
6
4
2
0
low
high
Risk

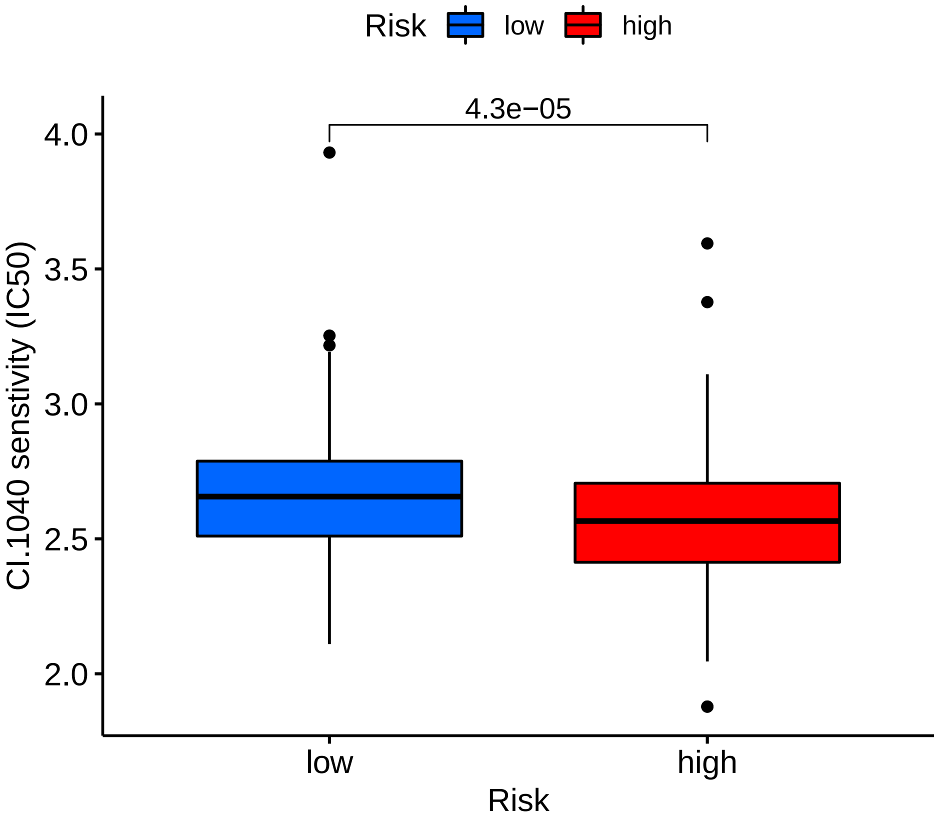
Risk
low
high
4.3e−05
4.0
3.5
3.0
2.5
2.0
CI.1040 senstivity (IC50)
low
high
Risk

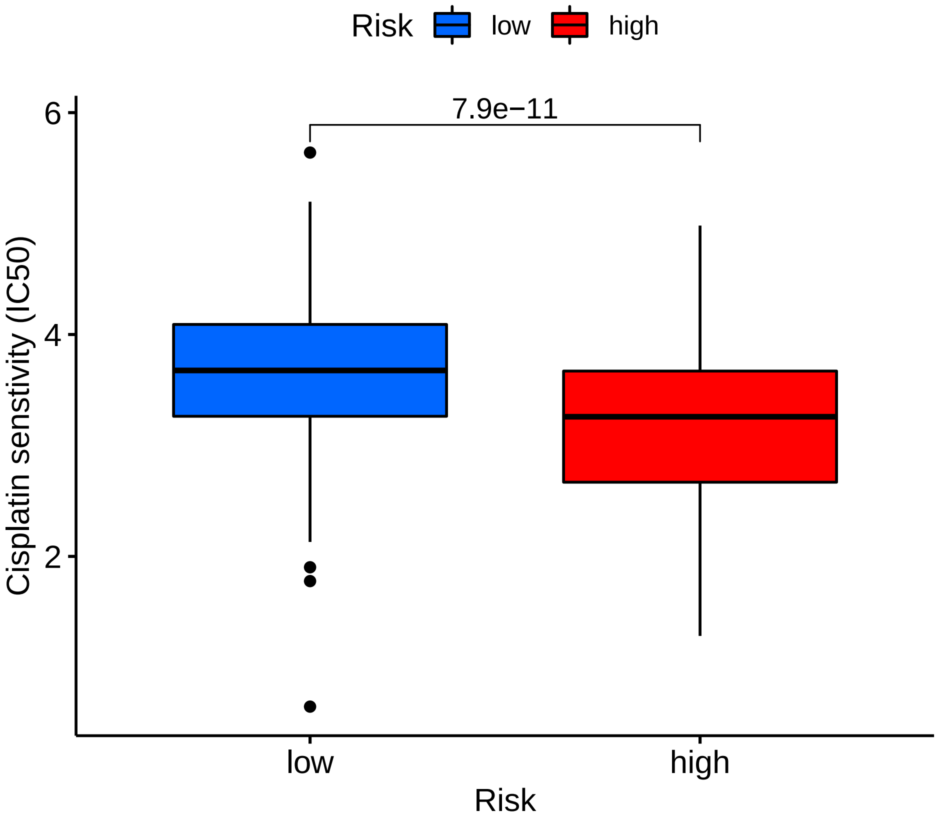

Risk
low
high
7.9e−11
Cisplatin senstivity (IC50)
6
4
2
low
high
Risk

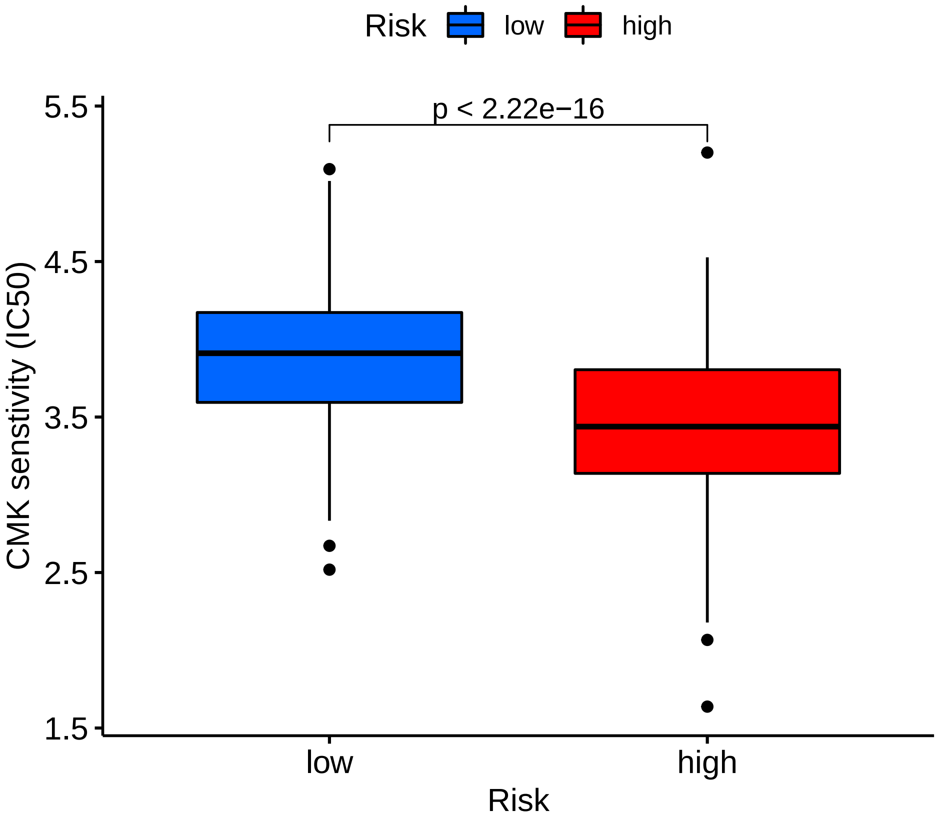

Risk
low
high
p < 2.22e−16
5.5
4.5
3.5
2.5
1.5
CMK senstivity (IC50)
low
high
Risk

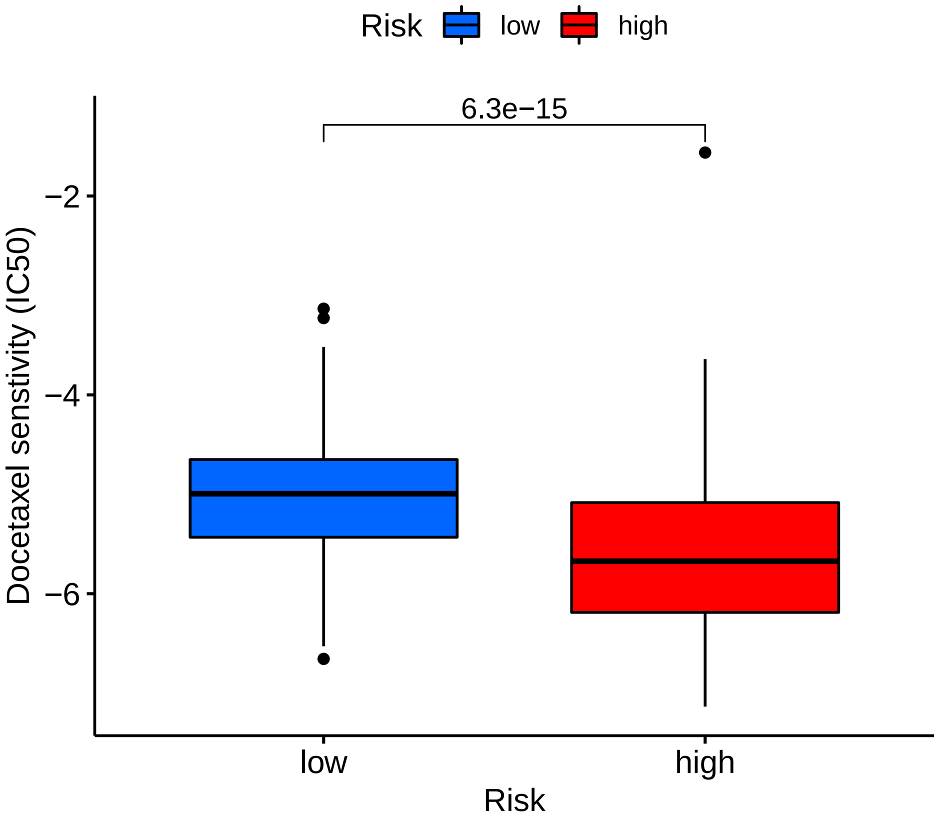

Risk
low
high
6.3e−15
−2
−4
−6
Docetaxel senstivity (IC50)
low
high
Risk

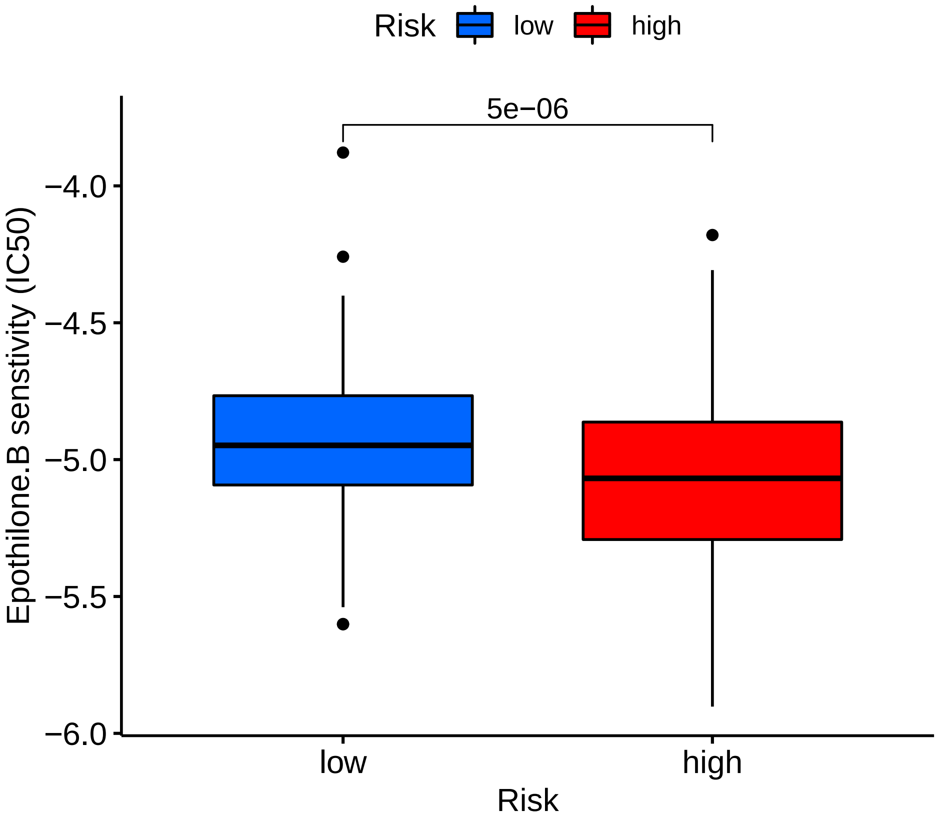
Risk
low
high
5e−06
−4.0
−4.5
−5.0
−5.5
−6.0
Epothilone.B senstivity (IC50)
low
high
Risk

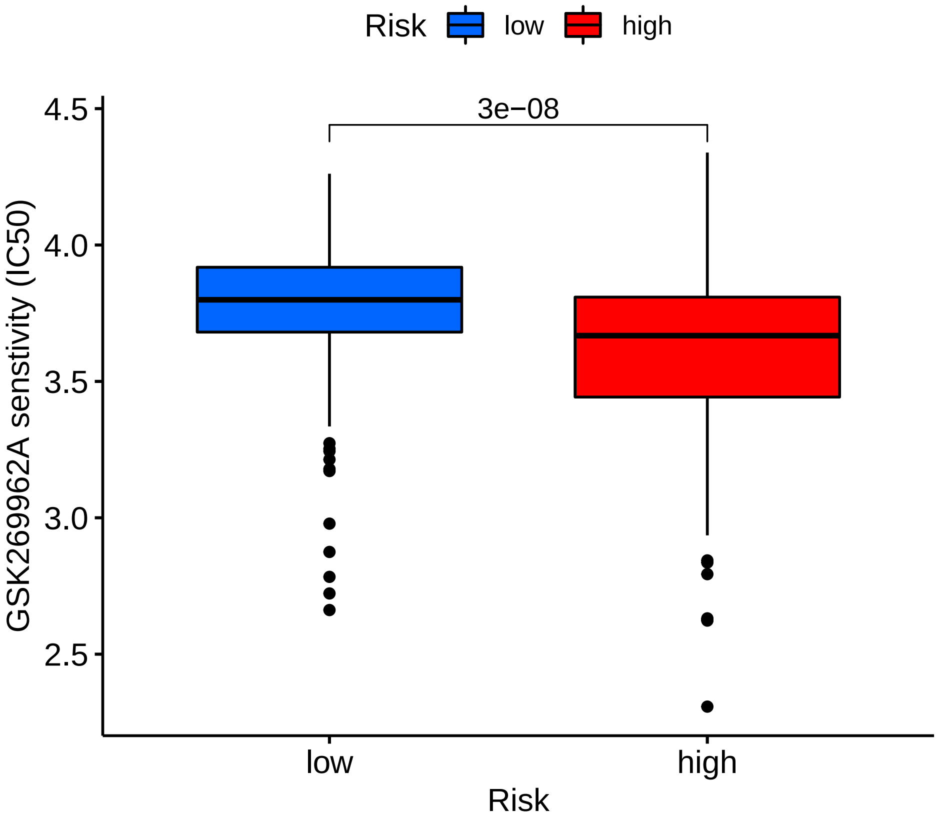

Risk
low
high
3e−08
4.5
4.0
3.5
3.0
2.5
GSK269962A senstivity (IC50)
low
high
Risk

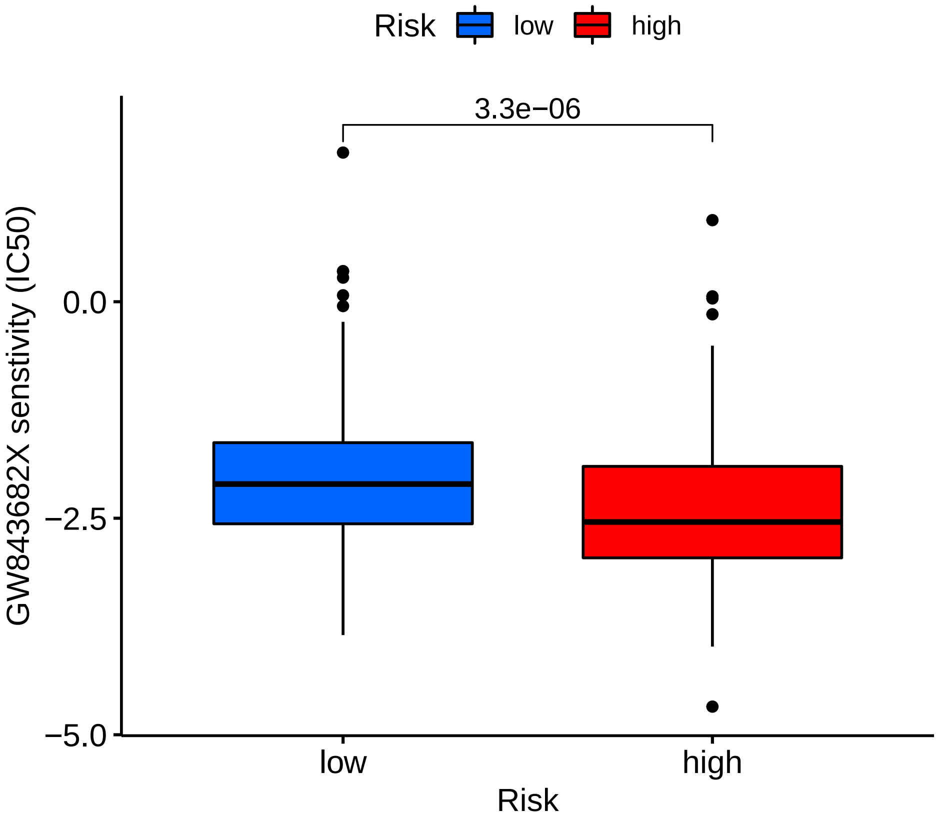
Risk
low
high
3.3e−06
GW843682X senstivity (IC50)
0.0
−2.5
−5.0
low
high
Risk

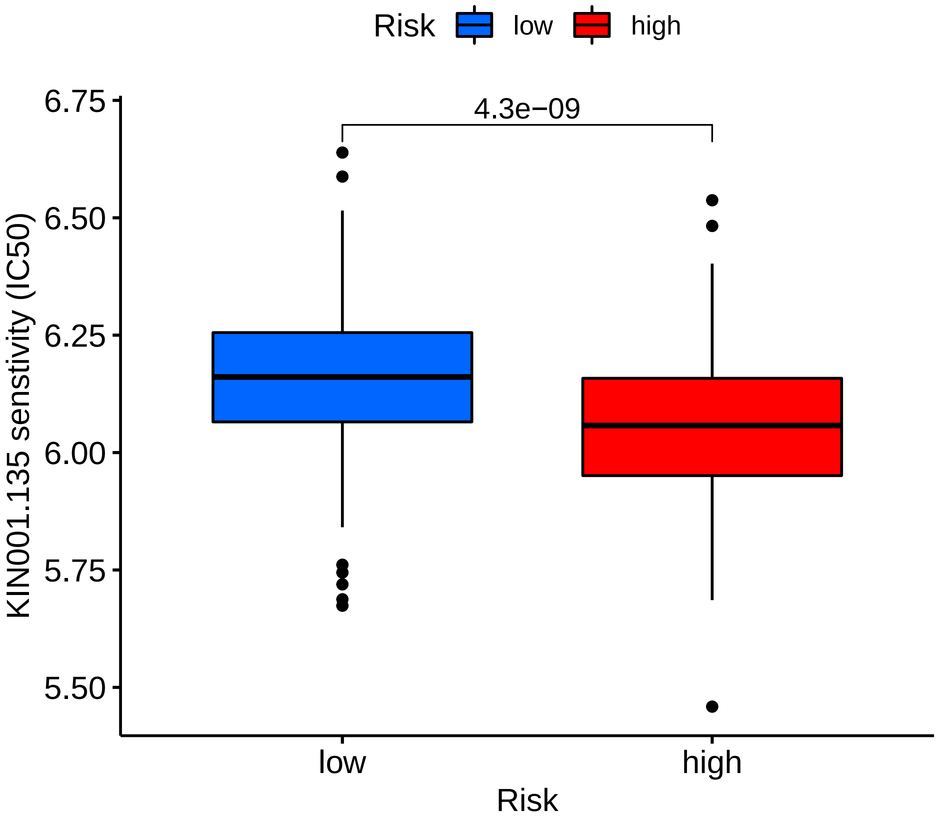

Risk
low
high
4.3e−09
6.75
6.50
6.25
6.00
5.75
5.50
KIN001.135 senstivity (IC50)
low
high
Risk

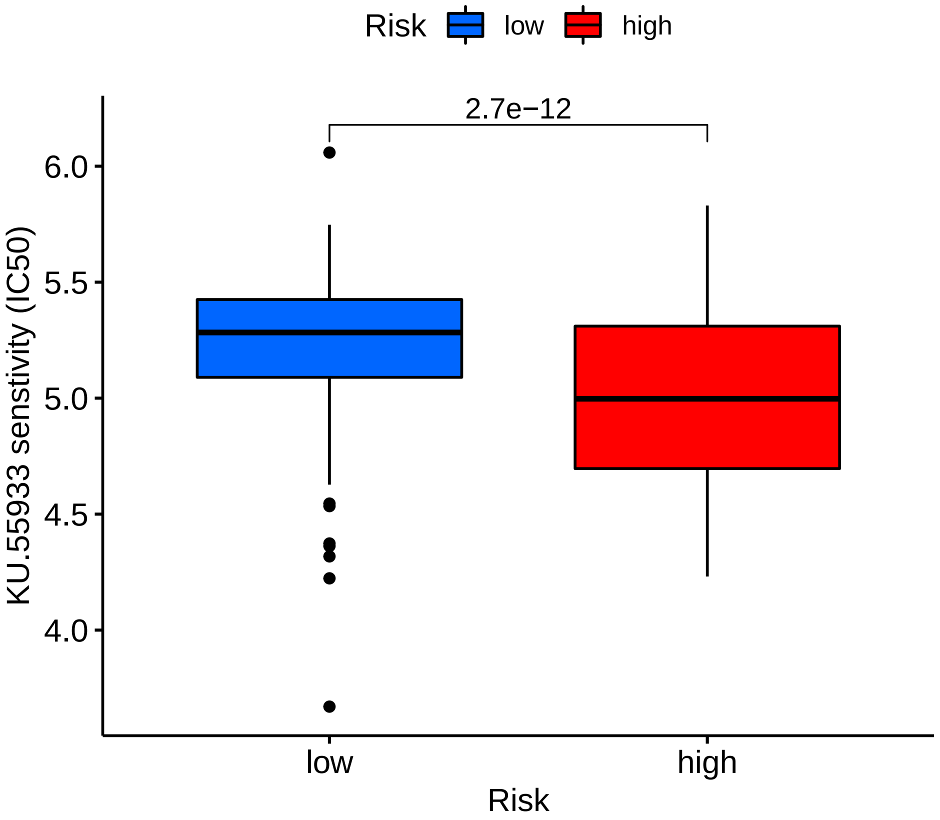
Risk
low
high
2.7e−12
6.0
5.5
5.0
4.5
4.0
KU.55933 senstivity (IC50)
low
high
Risk

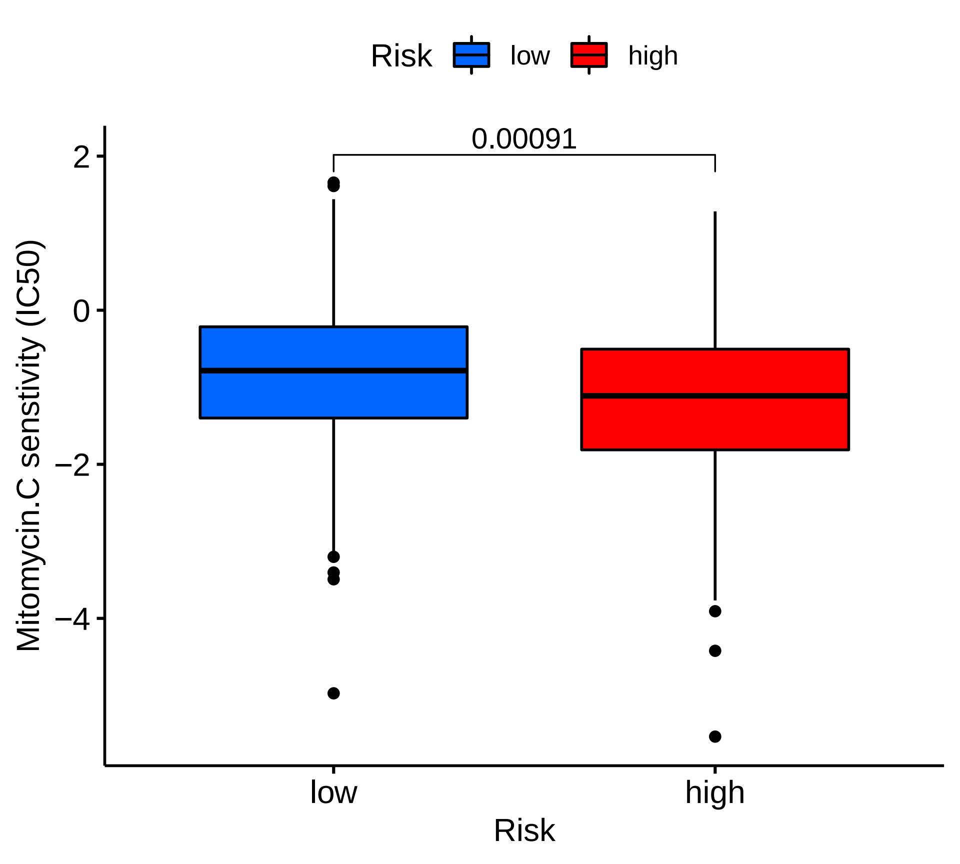
Risk
low
high
0.00091
2
0
−2
−4
Mitomycin.C senstivity (IC50)
low
high
Risk

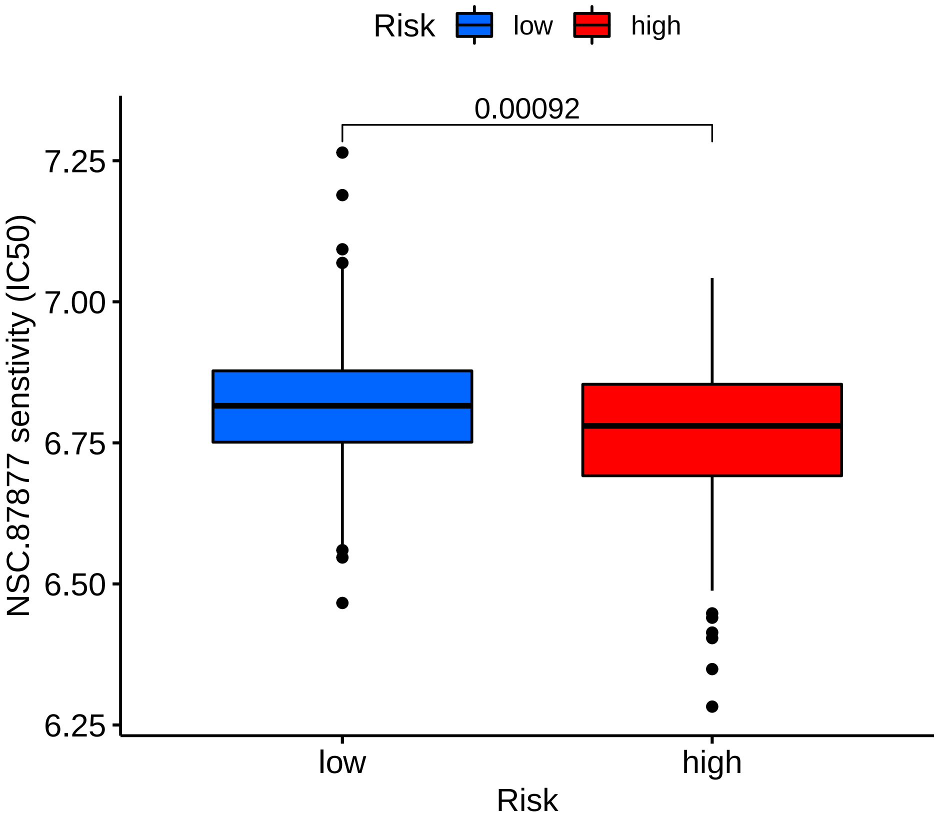

Risk
low
high
0.00092
7.25
7.00
6.75
6.50
6.25
NSC.87877 senstivity (IC50)
low
high
Risk

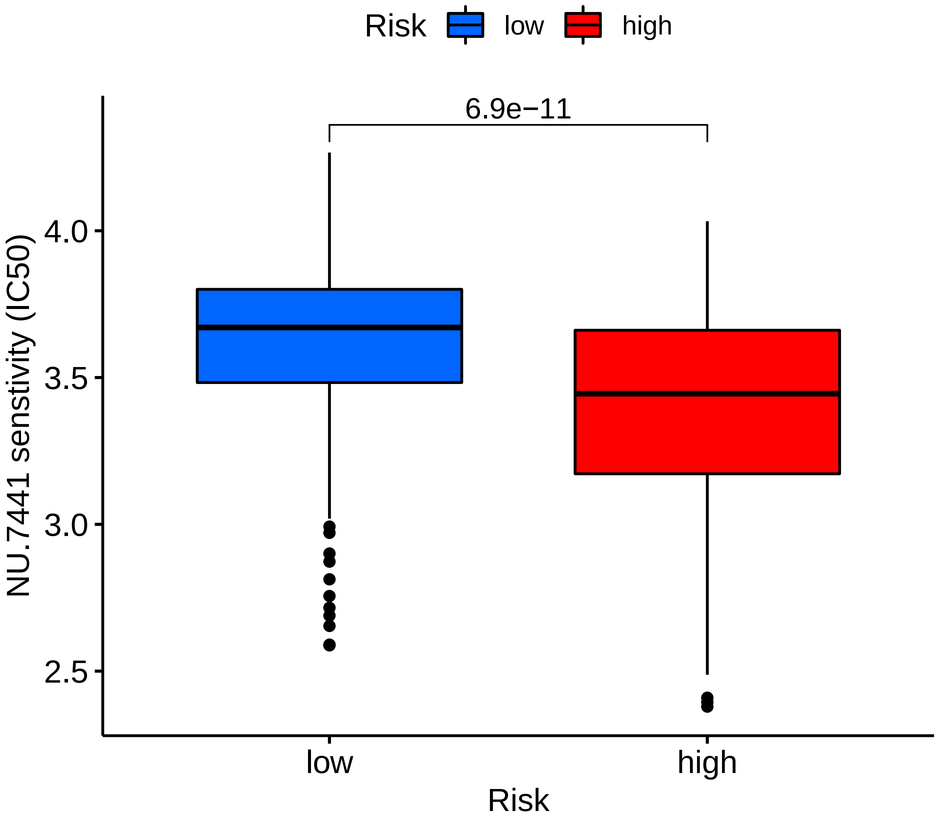

Risk
low
high
6.9e−11
NU.7441 senstivity (IC50)
4.0
3.5
3.0
2.5
low
high
Risk

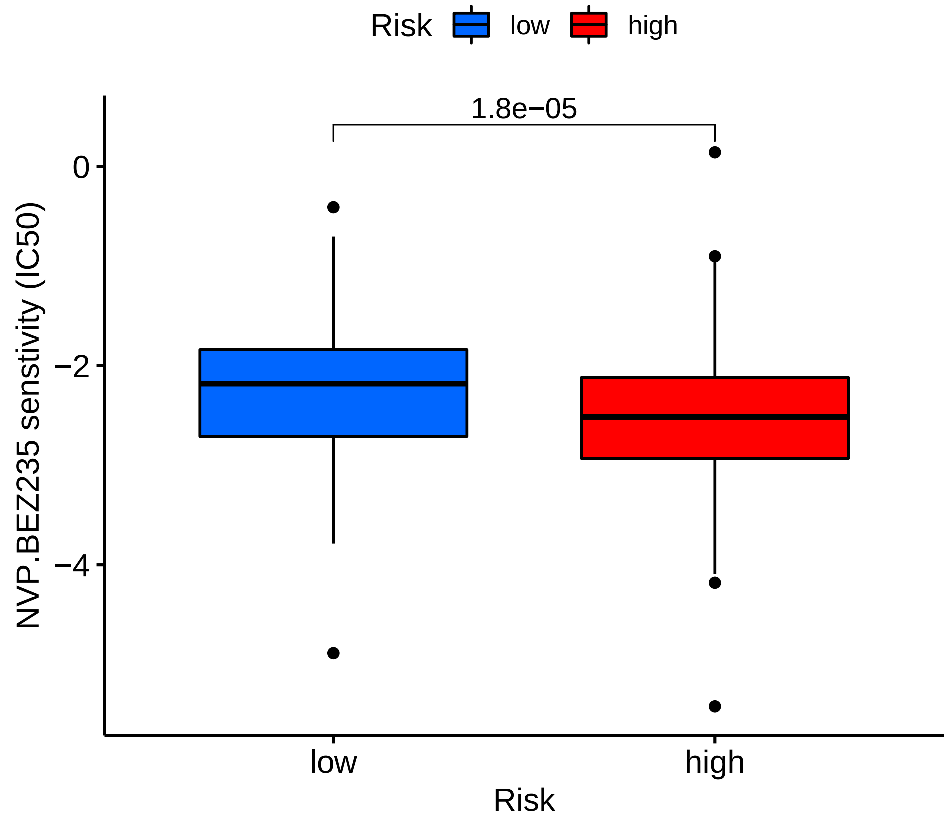

Risk
low
high
1.8e−05
0
−2
−4
NVP.BEZ235 senstivity (IC50)
low
high
Risk

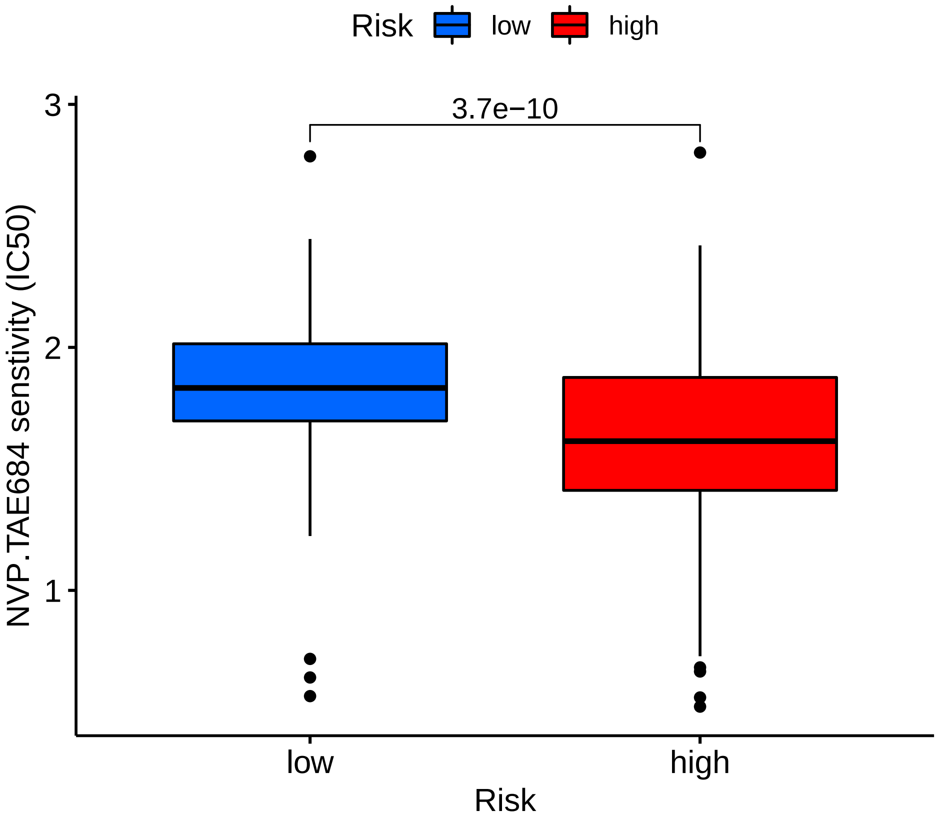
Risk
low
high
3.7e−10
NVP.TAE684 senstivity (IC50)
3
2
1
low
high
Risk

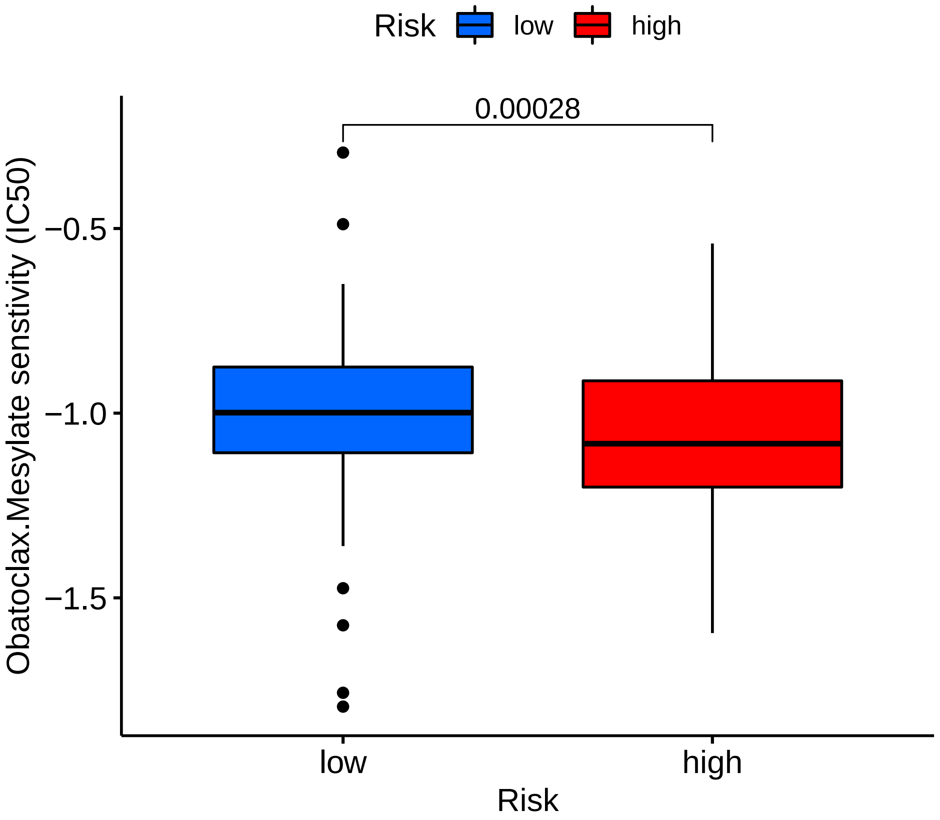

Risk
low
high
0.00028
−0.5
−1.0
−1.5
Obatoclax.Mesylate senstivity (IC50)
low
high
Risk

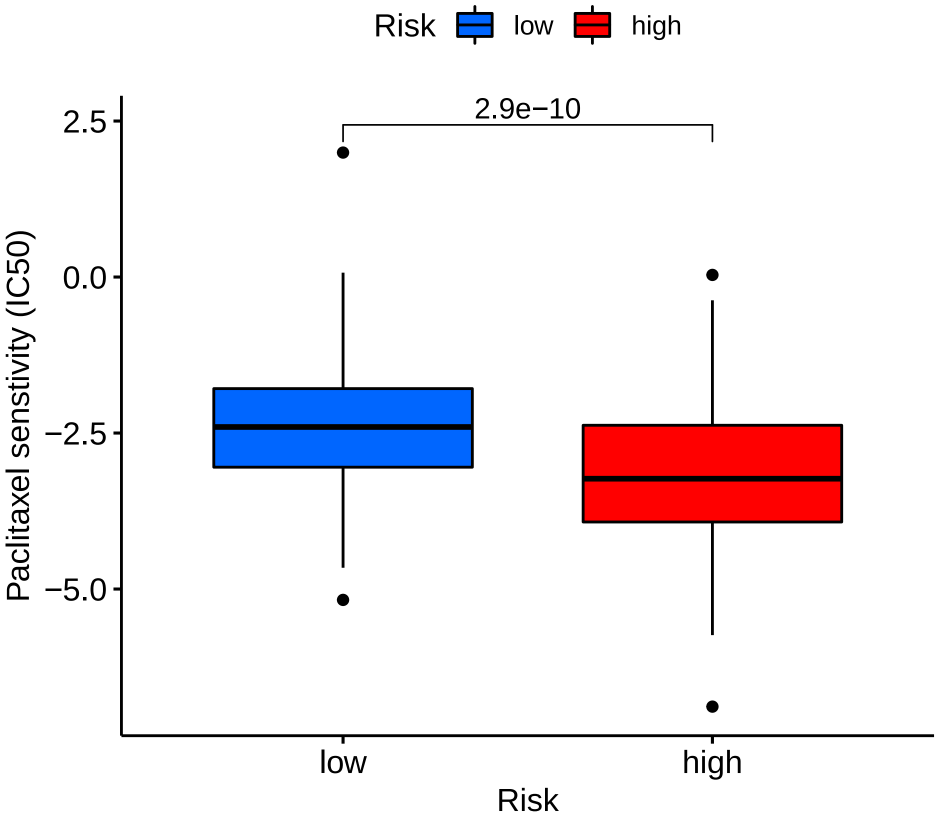

Risk
low
high
2.9e−10
2.5
0.0
−2.5
−5.0
Paclitaxel senstivity (IC50)
low
high
Risk

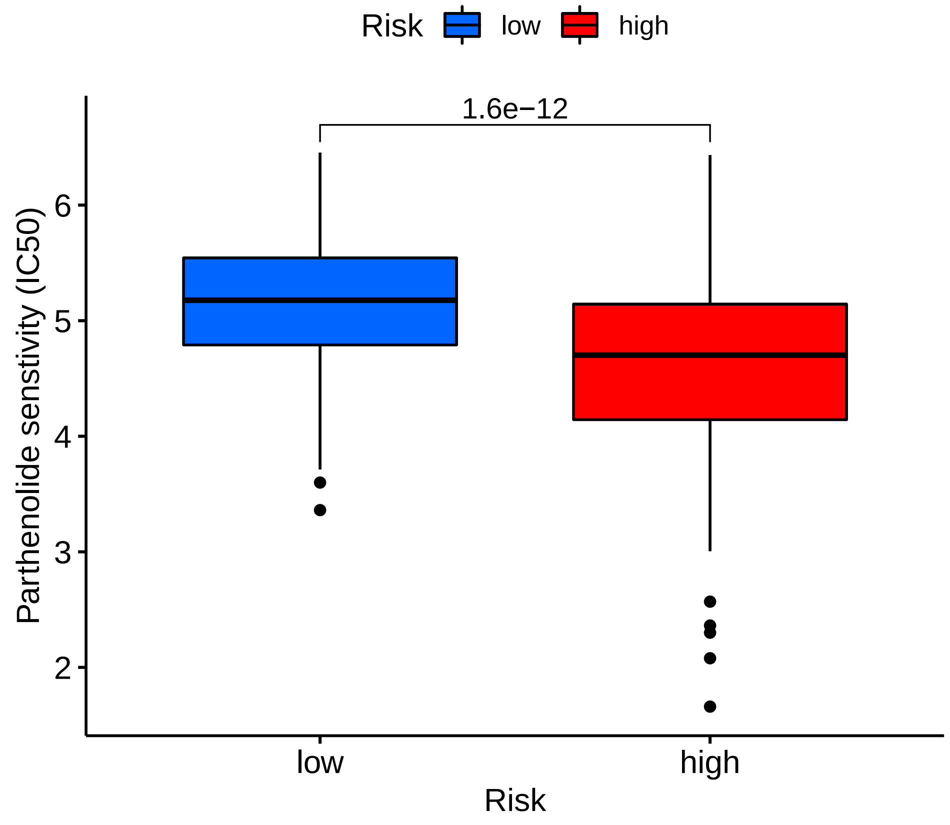

Risk
low
high
1.6e−12
Parthenolide senstivity (IC50)
6
5
4
3
2
low
high
Risk

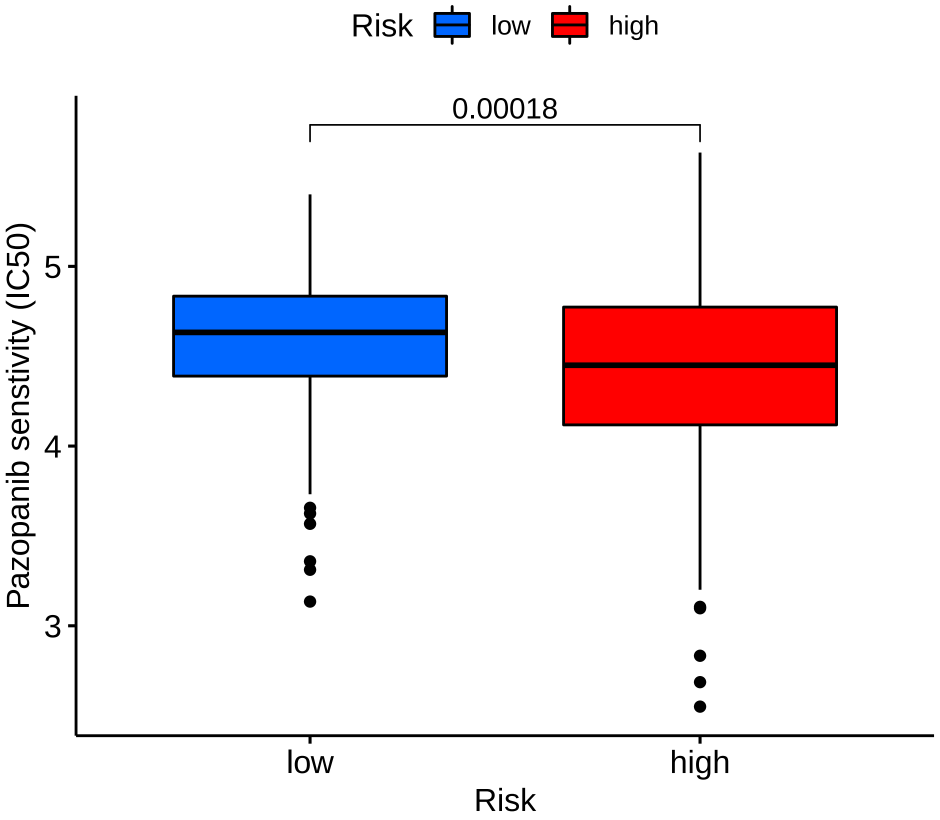

Risk
low
high
0.00018
Pazopanib senstivity (IC50)
5
4
3
low
high
Risk

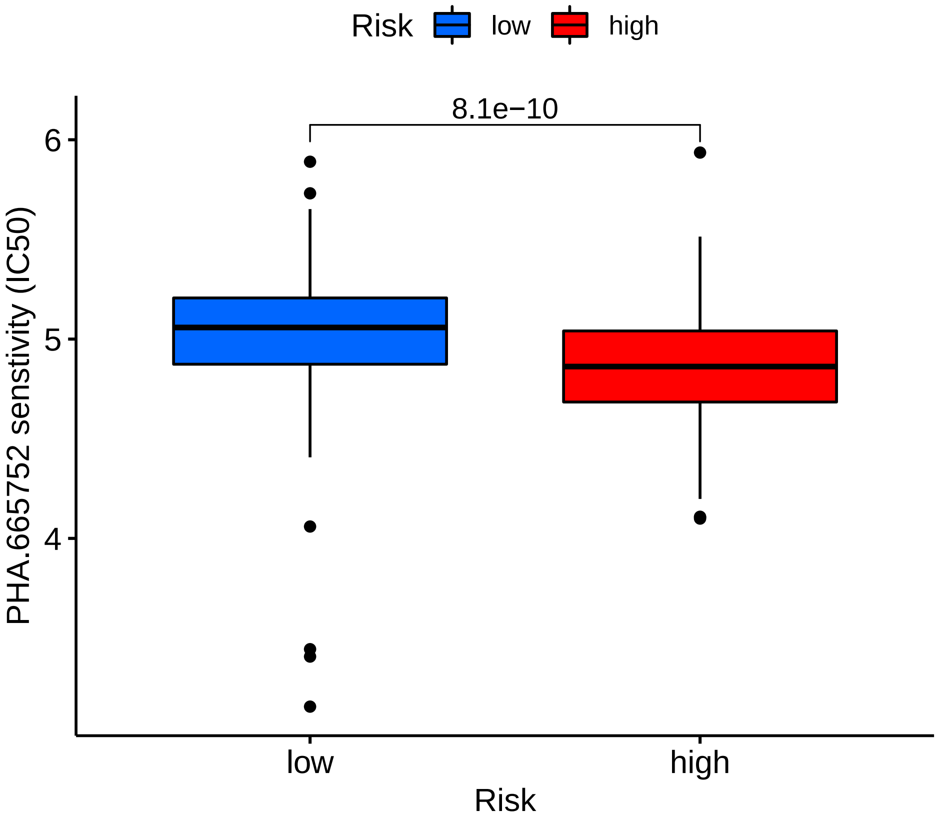

Risk
low
high
8.1e−10
6
5
4
PHA.665752 senstivity (IC50)
low
high
Risk

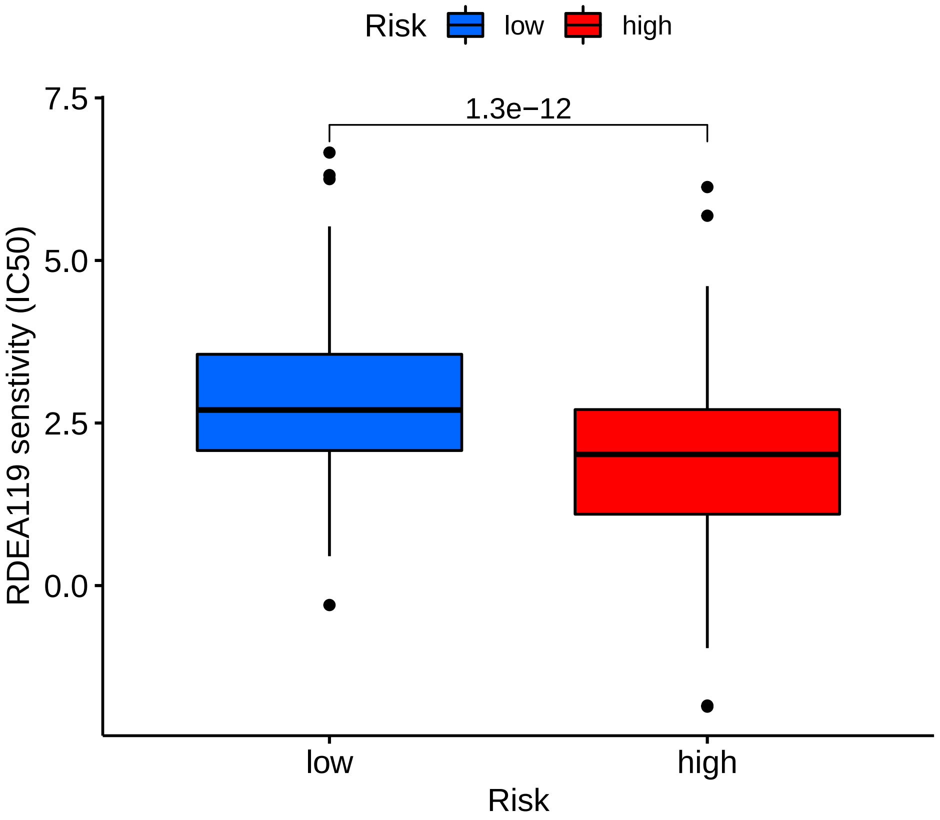

Risk
low
high
7.5
5.0
2.5
0.0
1.3e−12
RDEA119 senstivity (IC50)
low
high
Risk

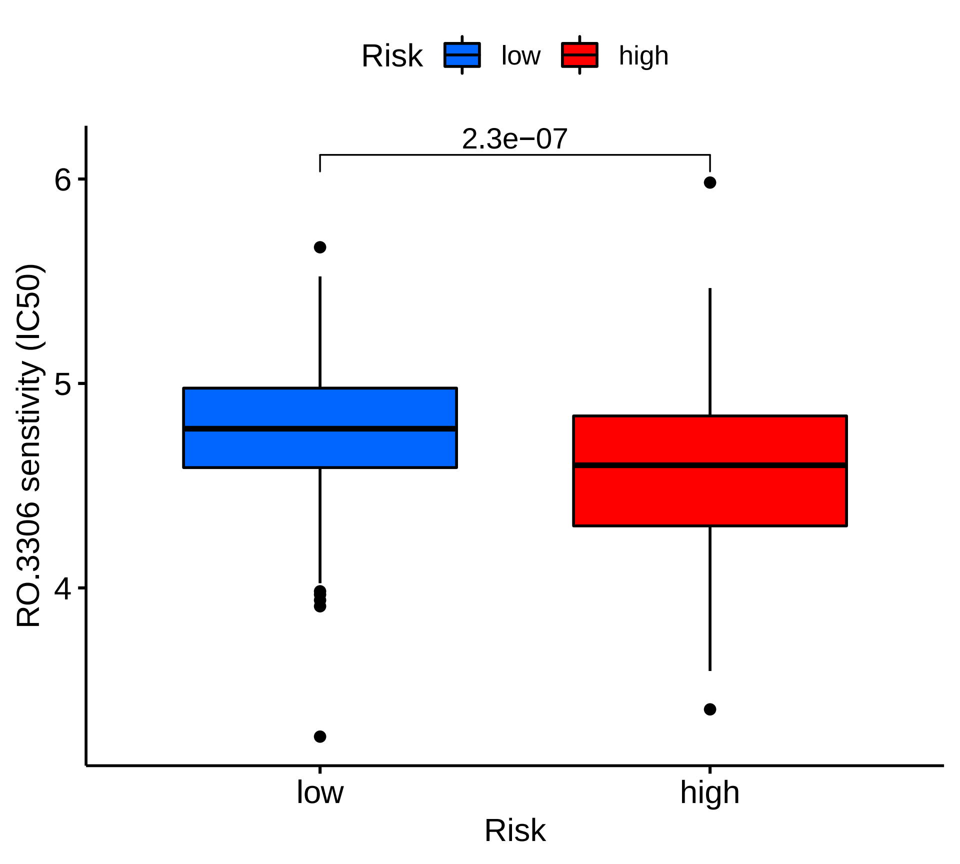

Risk
low
high
2.3e−07
6
5
4
RO.3306 senstivity (IC50)
low
high
Risk

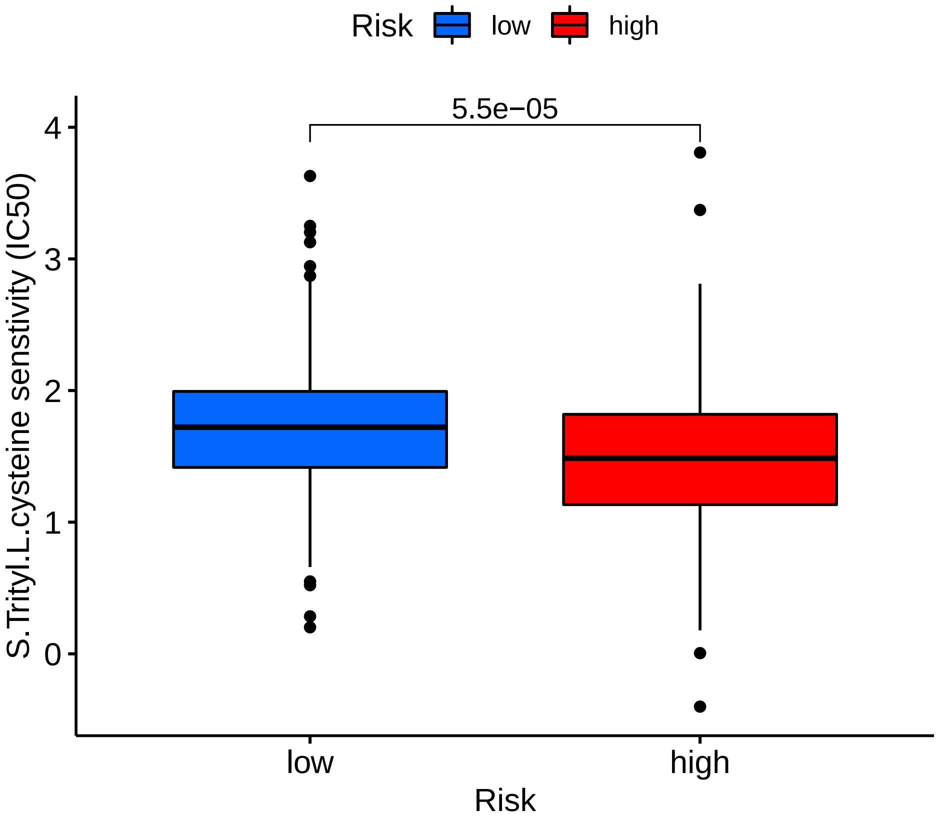

Risk
low
high
5.5e−05
S.Trityl.L.cysteine senstivity (IC50)
4
3
2
1
0
low
high
Risk

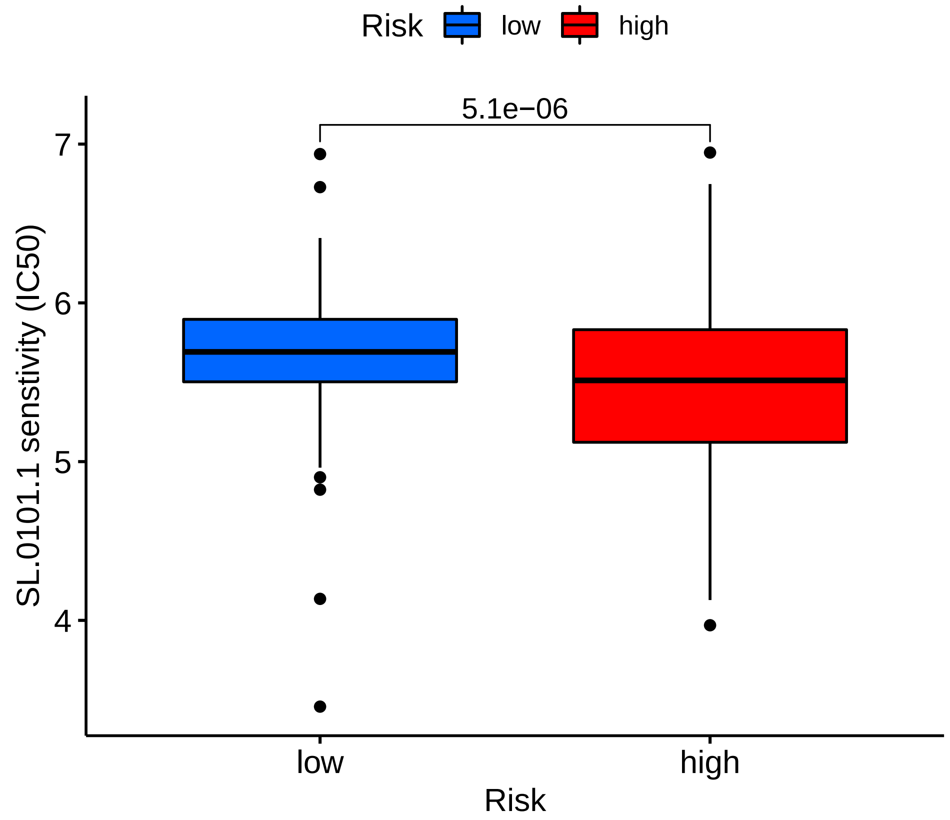
Risk
low
high
5.1e−06
7
6
5
4
SL.0101.1 senstivity (IC50)
low
high
Risk

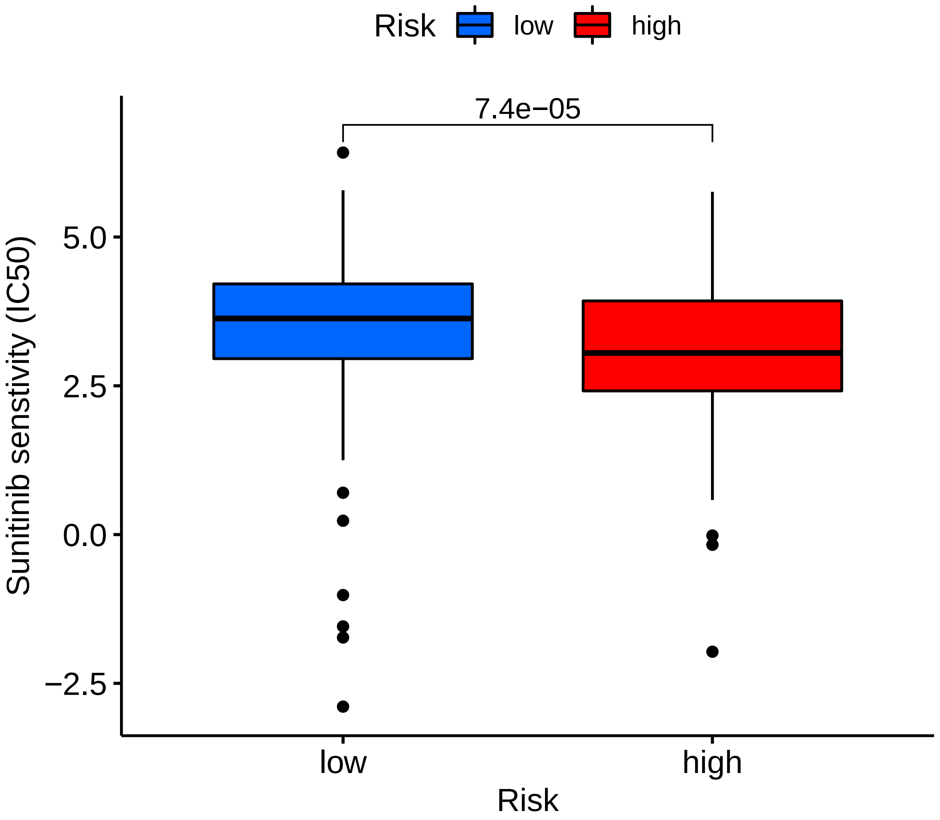

Risk
low
high
7.4e−05
Sunitinib senstivity (IC50)
5.0
2.5
0.0
−2.5
low
high
Risk

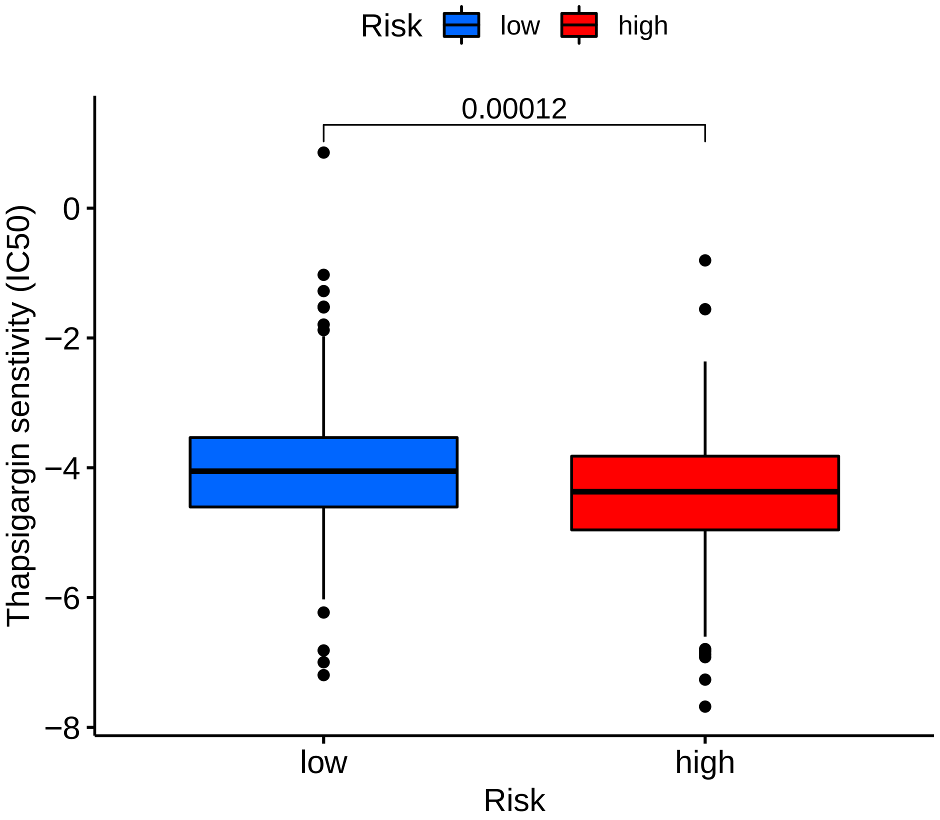

Risk
low
high
0.00012
Thapsigargin senstivity (IC50)
0
−2
−4
−6
−8
low
high
Risk

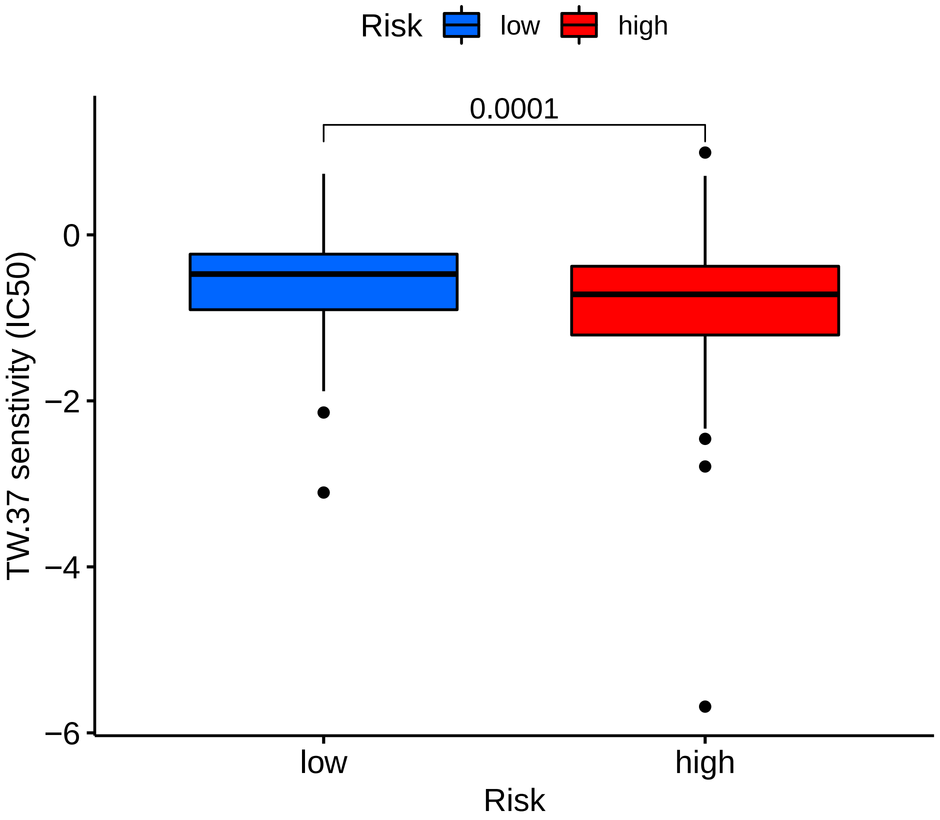

Risk
low
high
0.0001
TW.37 senstivity (IC50)
0
−2
−4
−6
low
high
Risk

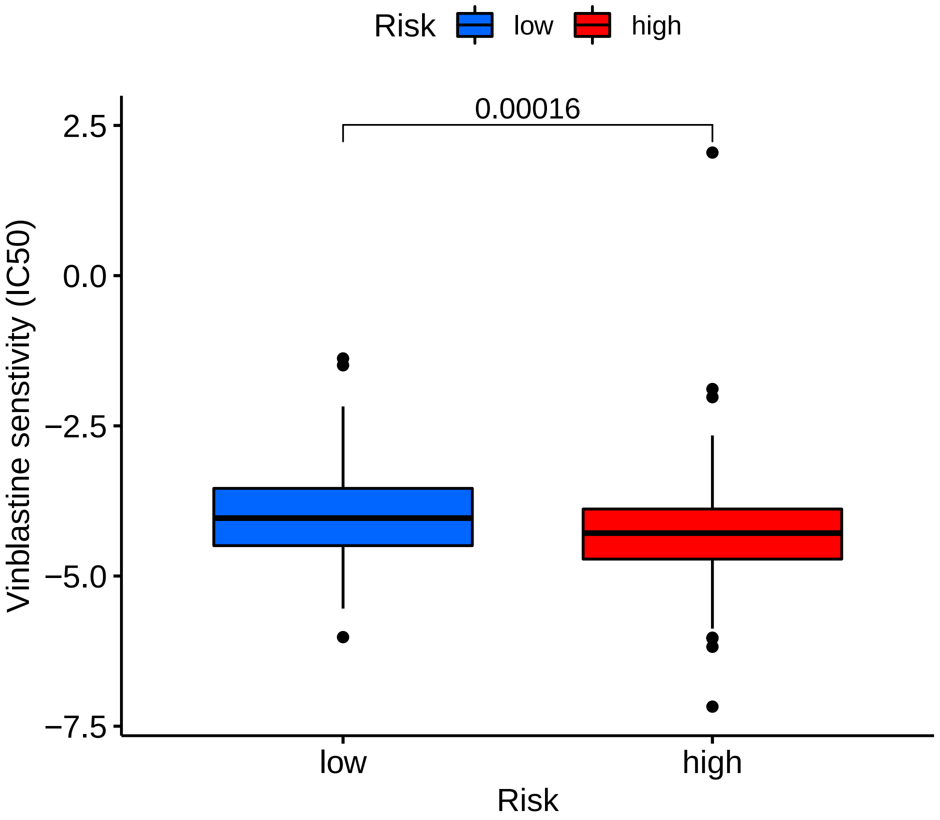

Risk
low
high
0.00016
2.5
0.0
−2.5
−5.0
−7.5
Vinblastine senstivity (IC50)
low
high
Risk

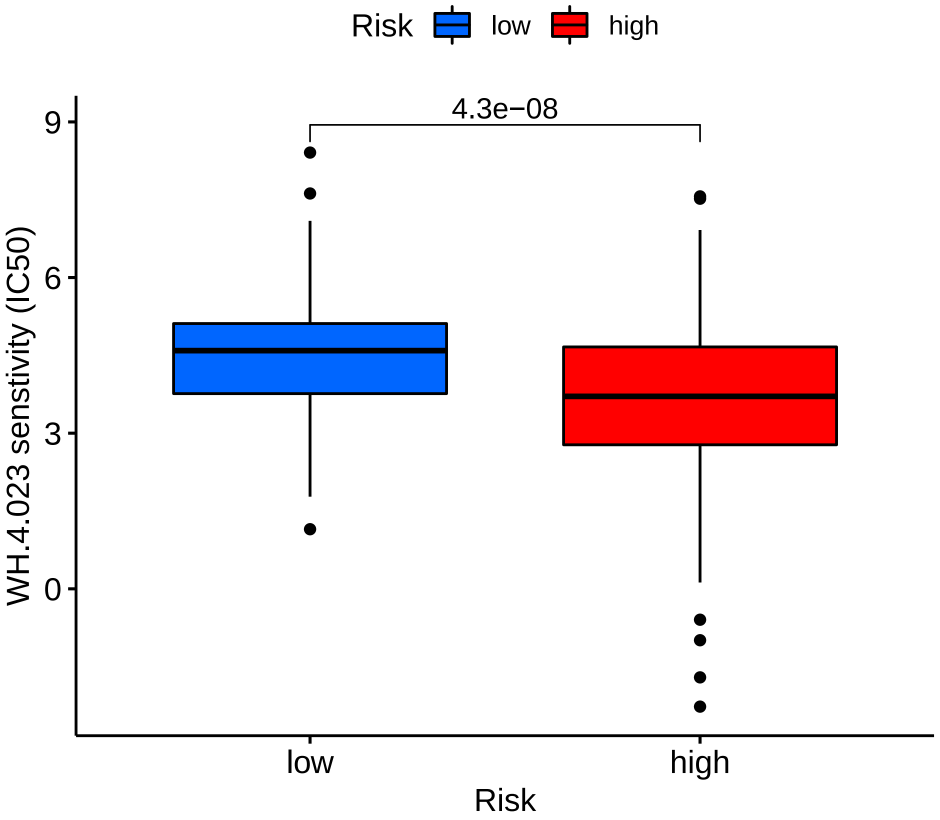

Risk
low
high
4.3e−08
WH.4.023 senstivity (IC50)
9
6
3
0
low
high
Risk

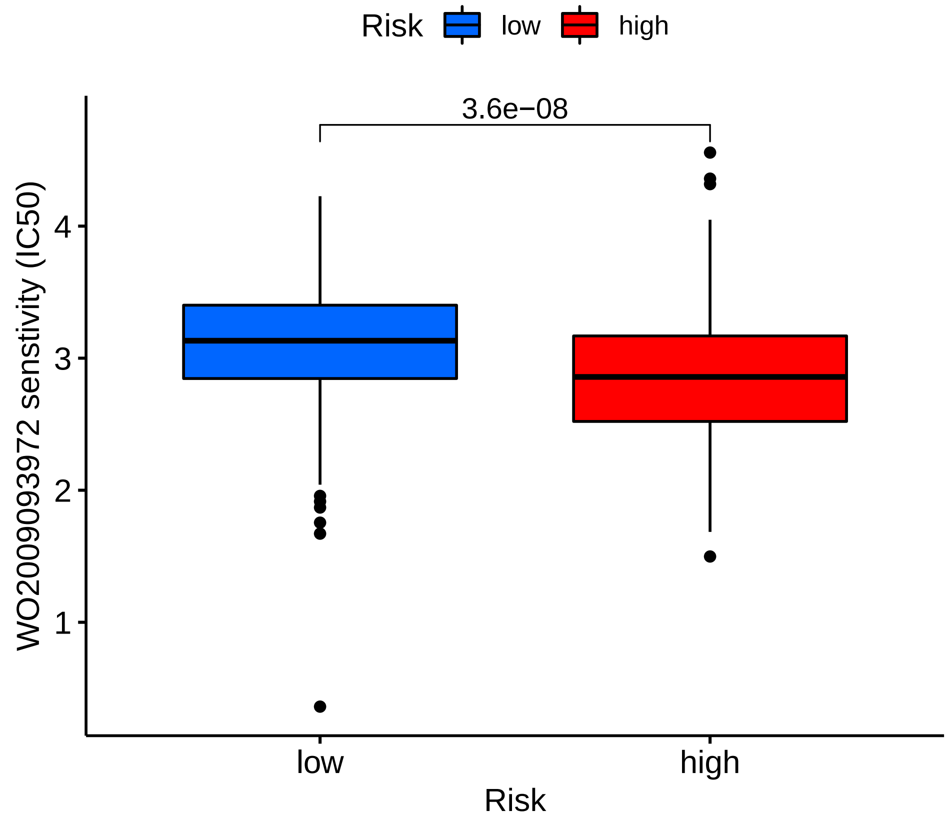

Risk
low
high
3.6e−08
WO2009093972 senstivity (IC50)
4
3
2
1
low
high
Risk

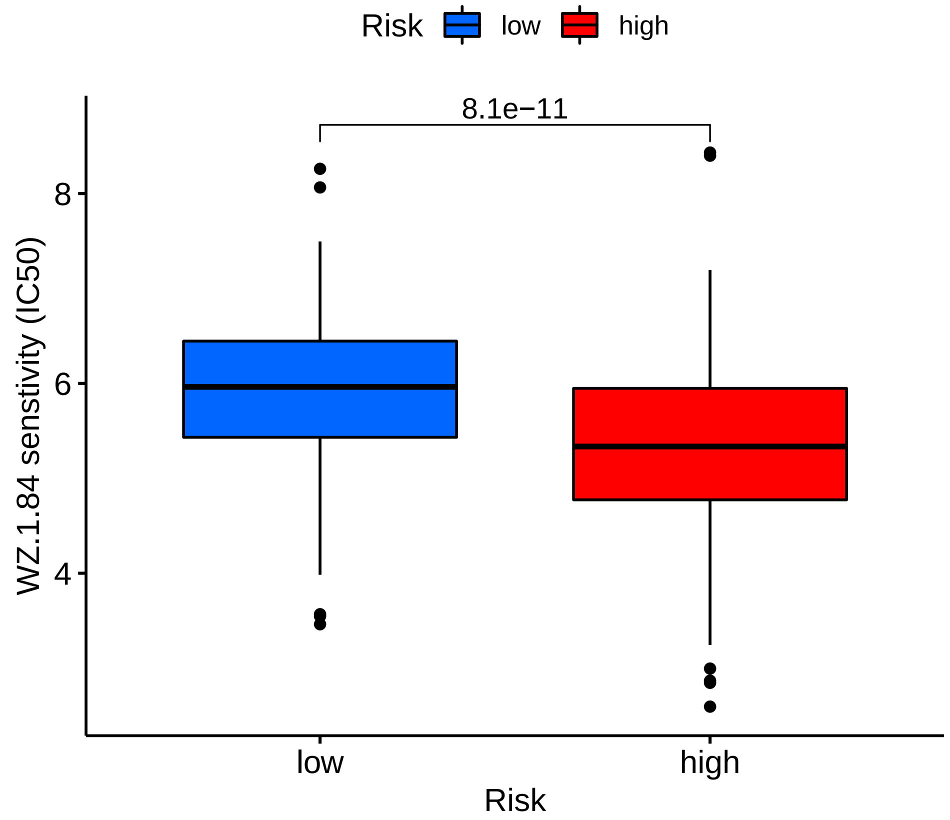
Risk
low
high
8.1e−11
WZ.1.84 senstivity (IC50)
8
6
4
low
high
Risk

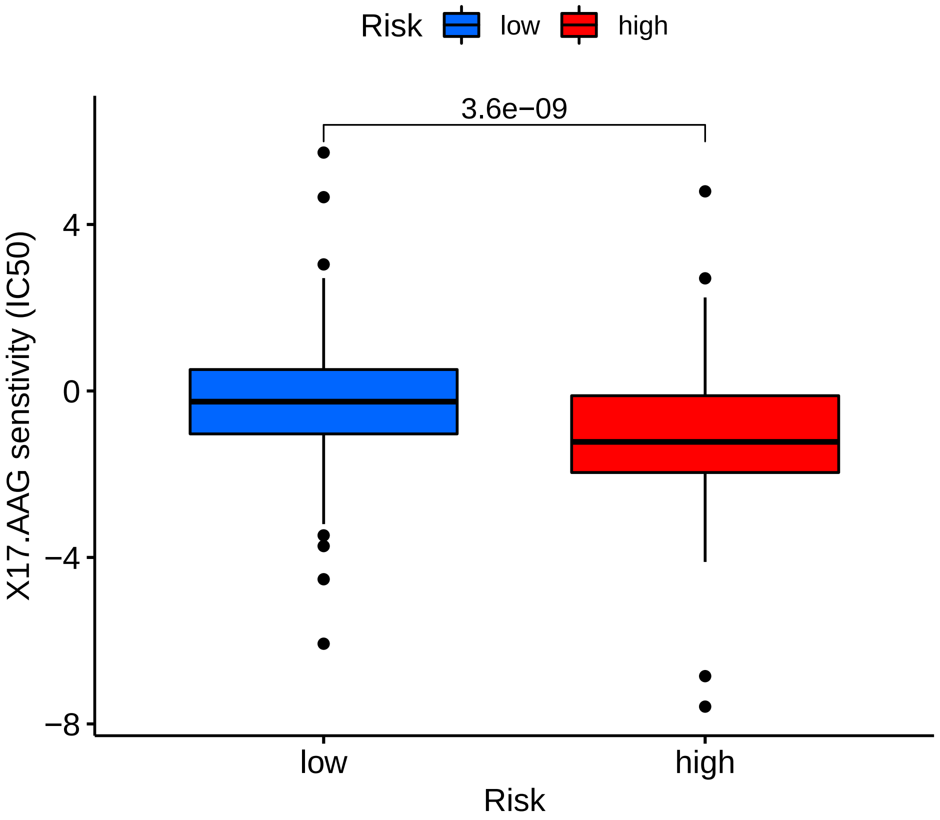

Risk
low
high
3.6e−09
X17.AAG senstivity (IC50)
4
0
−4
−8
low
high
Risk

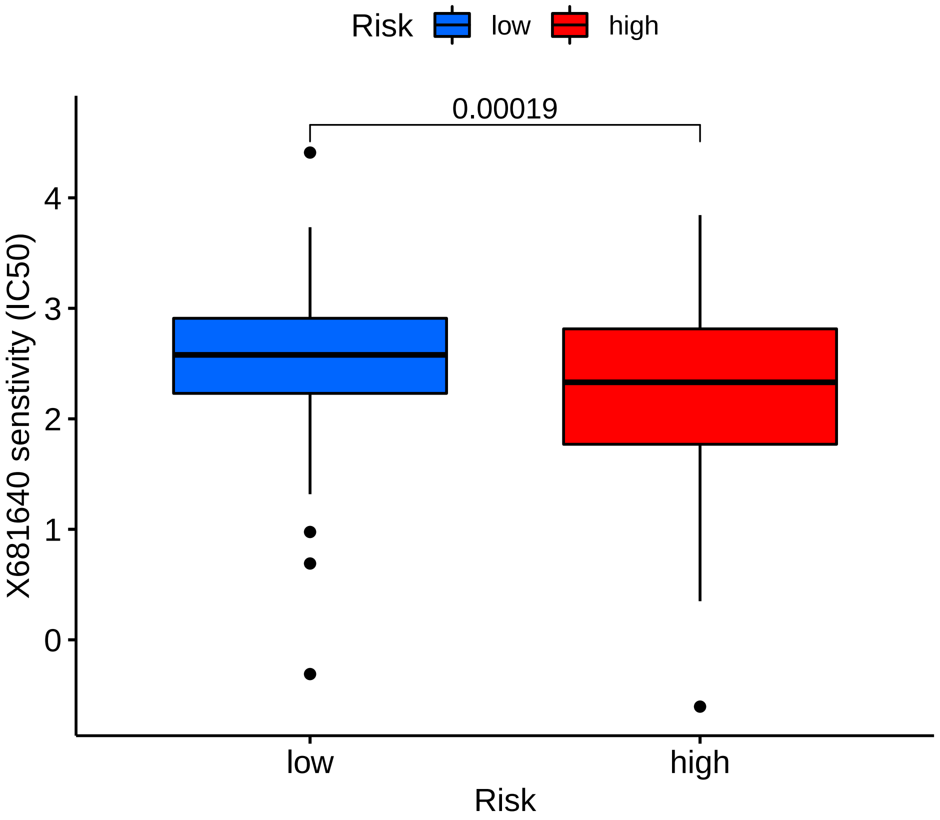

Risk
low
high
0.00019
X681640 senstivity (IC50)
4
3
2
1
0
low
high
Risk
