# Supplementary material for: Predicting Prognosis and Distinguishing Cold and Hot Tumors in Bladder Urothelial Carcinoma Based on Necroptosis-Associated lncRNAs
Source: Front Immunol. 2022 Jul 4;13:916800. doi: 10.3389/fimmu.2022.916800 (PMC9289196; doi:10.3389/fimmu.2022.916800)
Supplement: Supplementary Figure 2 — Estimation of consensus clustering of necroptosis-associated lncRNAs. [file DataSheet_2.pdf]

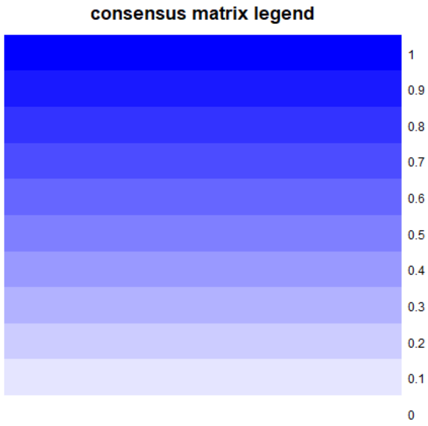
consensus matrix legend
1
0.9
0.8
0.7
0.6
0.5
0.4
0.3
0.2
0.1
0

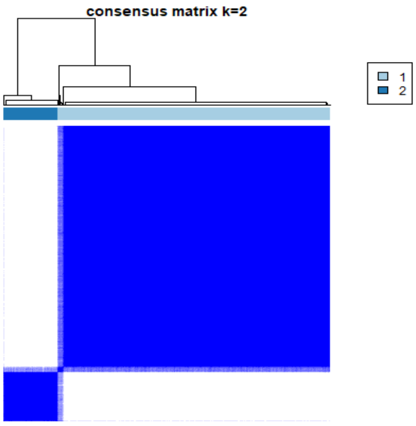
consensus matrix k=2
1
2

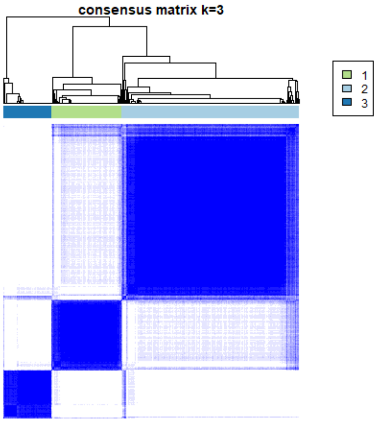
consensus matrix k=3
1
2
3

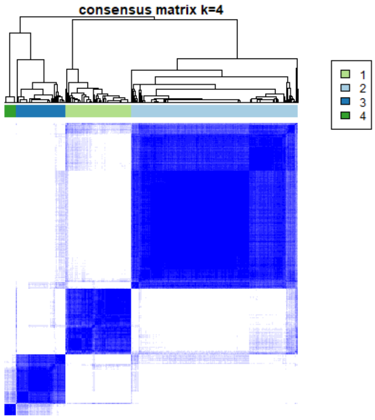
consensus matrix k=4
1
2
3
4

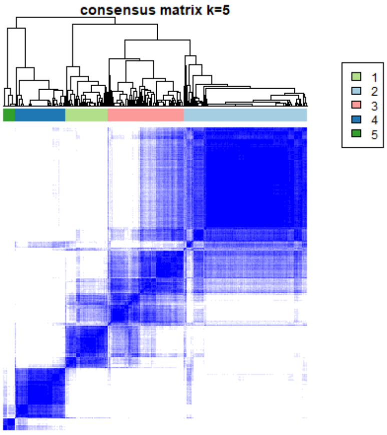
consensus matrix k=5
1
2
3
4
5

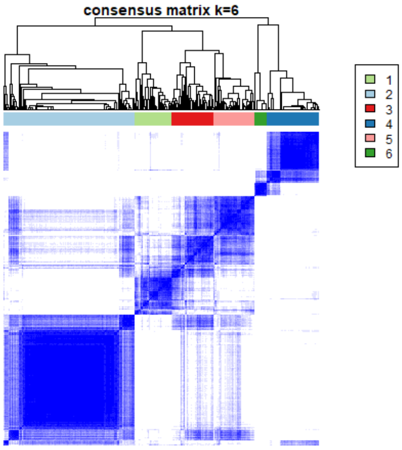
consensus matrix k=6
1
2
3
4
5
6

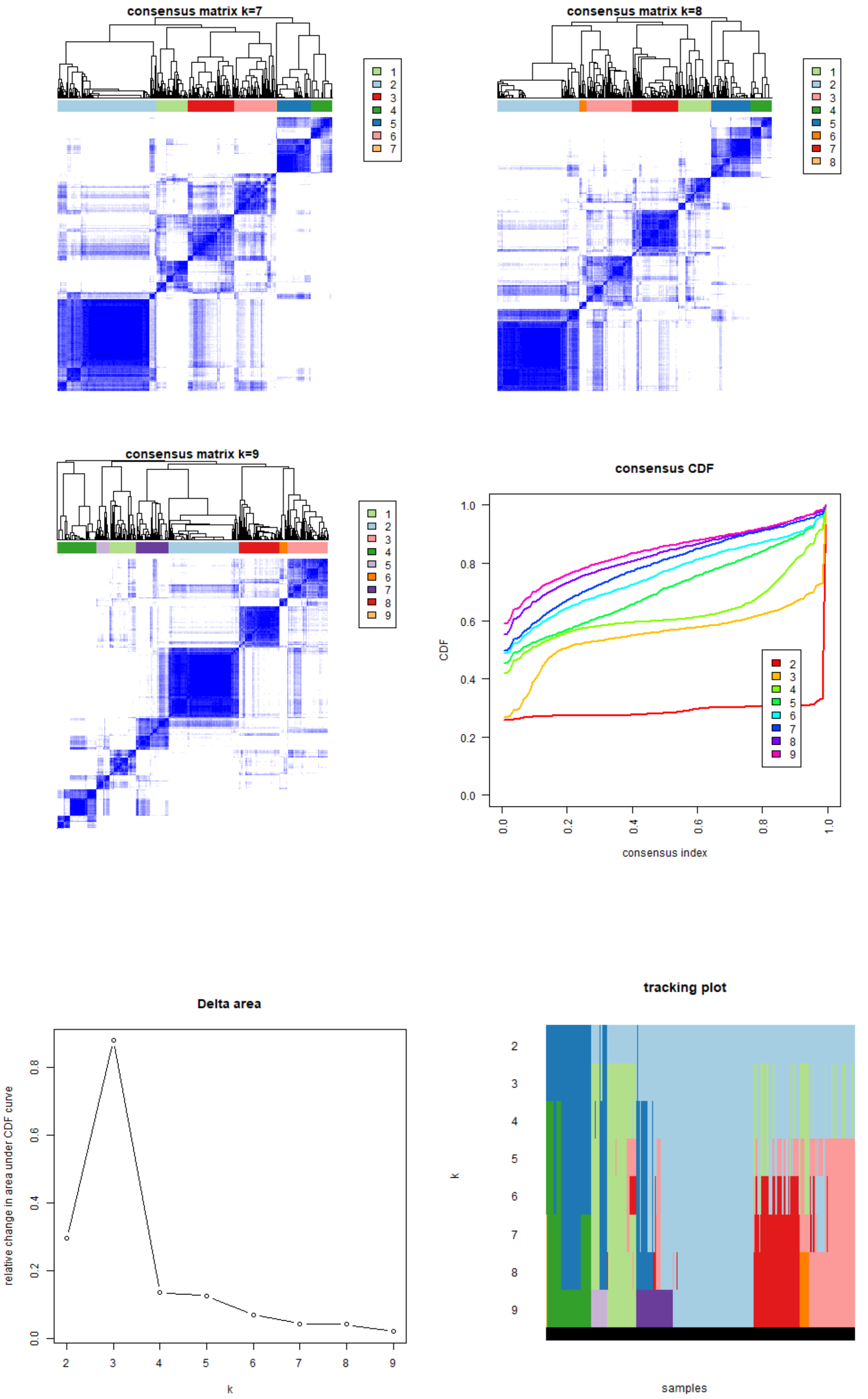
consensus matrix k=7
consensus matrix k=8
consensus matrix k=9
consensus CDF
CDF
consensus index
Delta area
relative change in area under CDF curve
k
tracking plot
k
samples
