# Supplementary material for: Predicting Prognosis and Distinguishing Cold and Hot Tumors in Bladder Urothelial Carcinoma Based on Necroptosis-Associated lncRNAs
Source: Front Immunol. 2022 Jul 4;13:916800. doi: 10.3389/fimmu.2022.916800 (PMC9289196; doi:10.3389/fimmu.2022.916800)
Supplement: Supplementary Figure 3 — Drug sensitivity analysis among clusters. [file DataSheet_3.pdf]

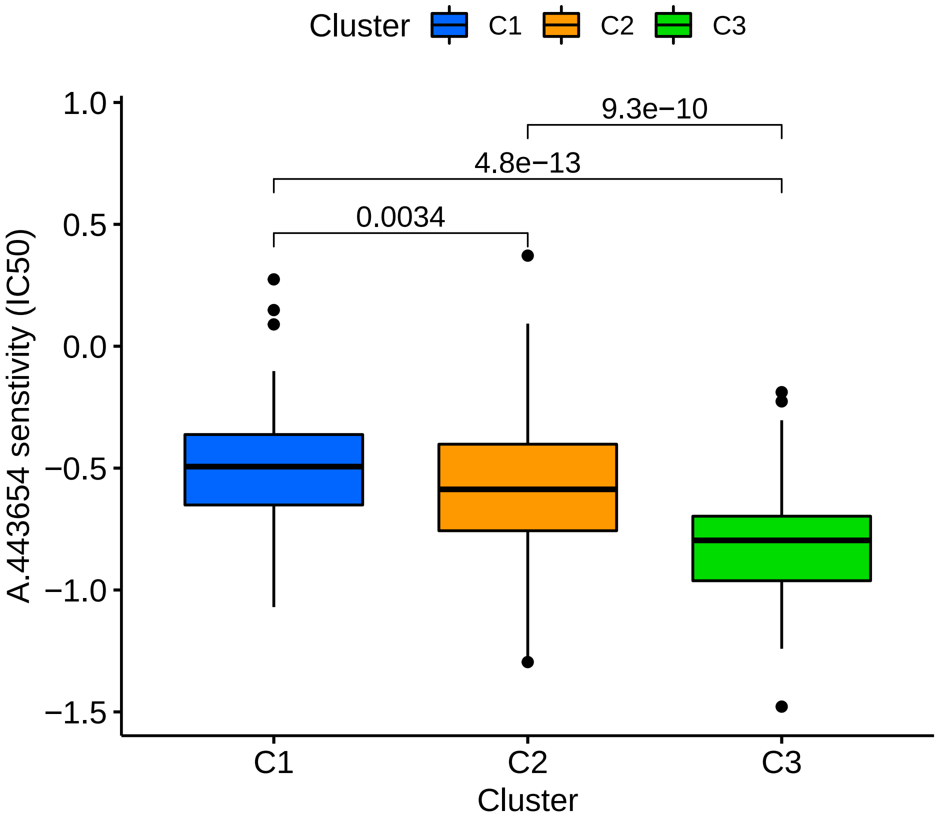

Cluster
C1
C2
C3
9.3e−10
4.8e−13
0.0034
1.0
0.5
0.0
−0.5
−1.0
−1.5
A.443654 senstivity (IC50)
C1
C2
C3
Cluster

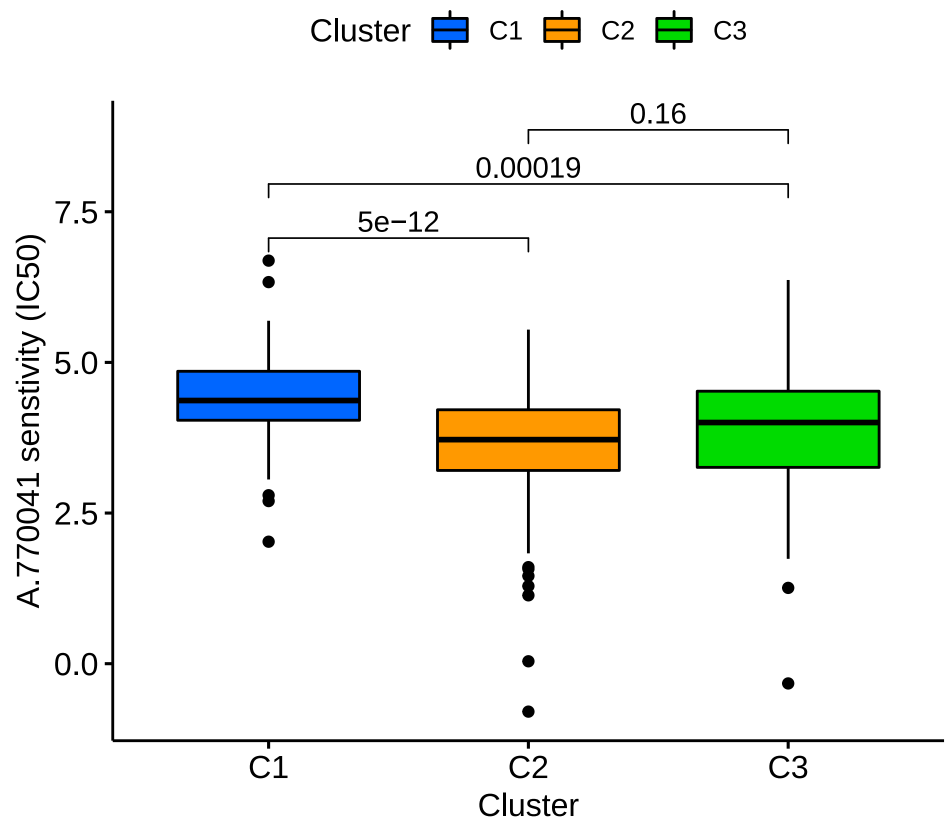
Cluster
C1
C2
C3
0.16
0.00019
5e−12
7.5
5.0
2.5
0.0
A.770041 senstivity (IC50)
C1
C2
C3
Cluster

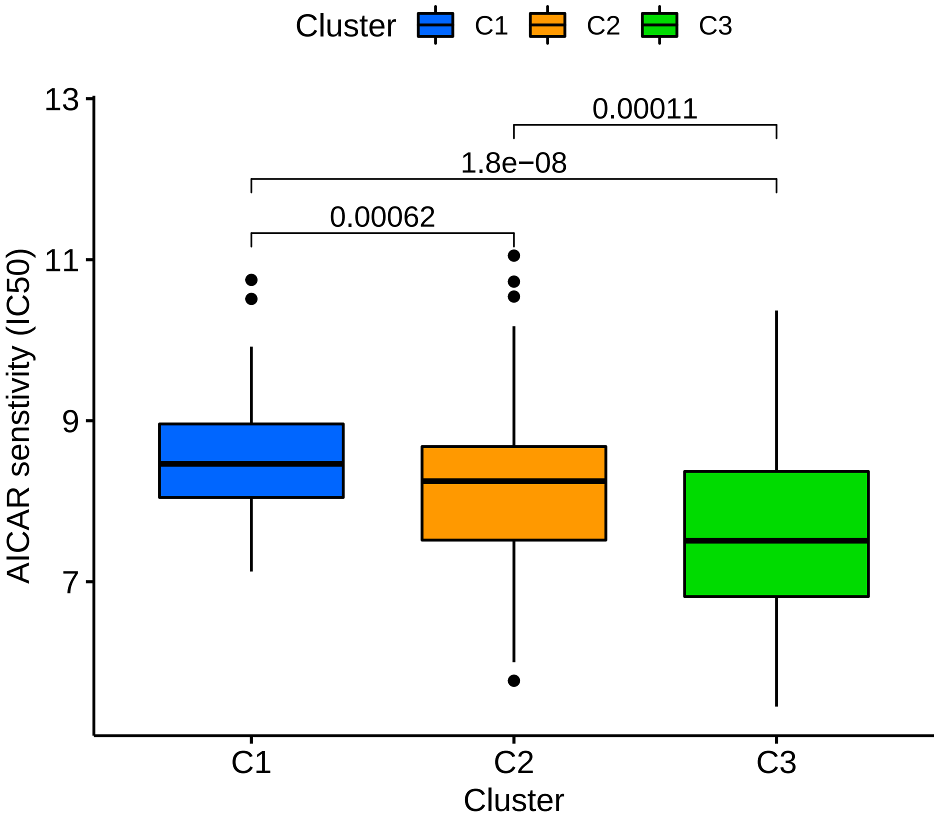
Cluster
C1
C2
C3
13
11
9
7
AICAR senstivity (IC50)
0.00011
1.8e−08
0.00062
C1
C2
C3
Cluster

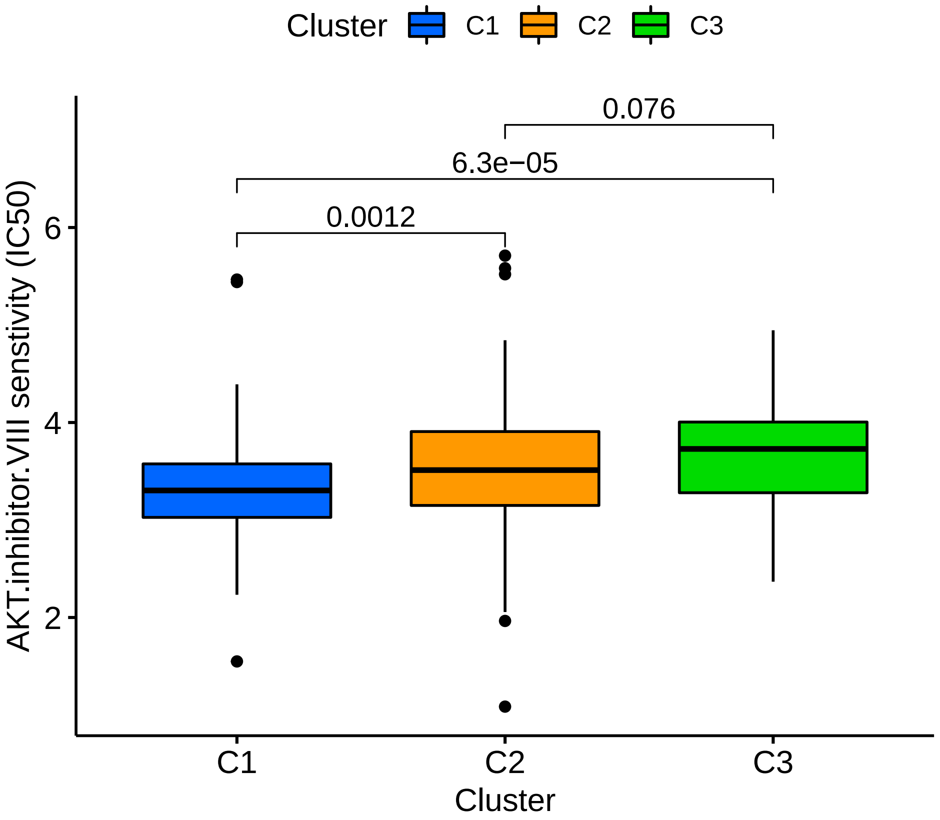
Cluster
C1
C2
C3
0.076
6.3e−05
0.0012
AKT.inhibitor.VIII senstivity (IC50)
6
4
2
C1
C2
C3
Cluster

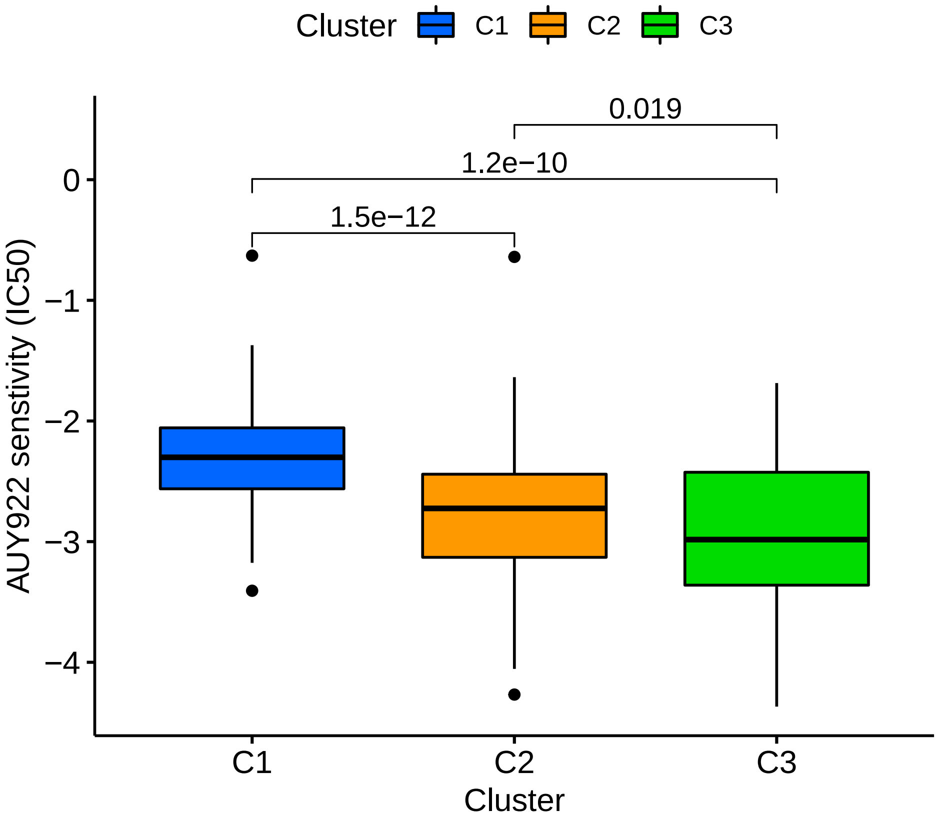

Cluster
C1
C2
C3
0.019
1.2e−10
1.5e−12
0
−1
−2
−3
−4
AUY922 senstivity (IC50)
C1
C2
C3
Cluster

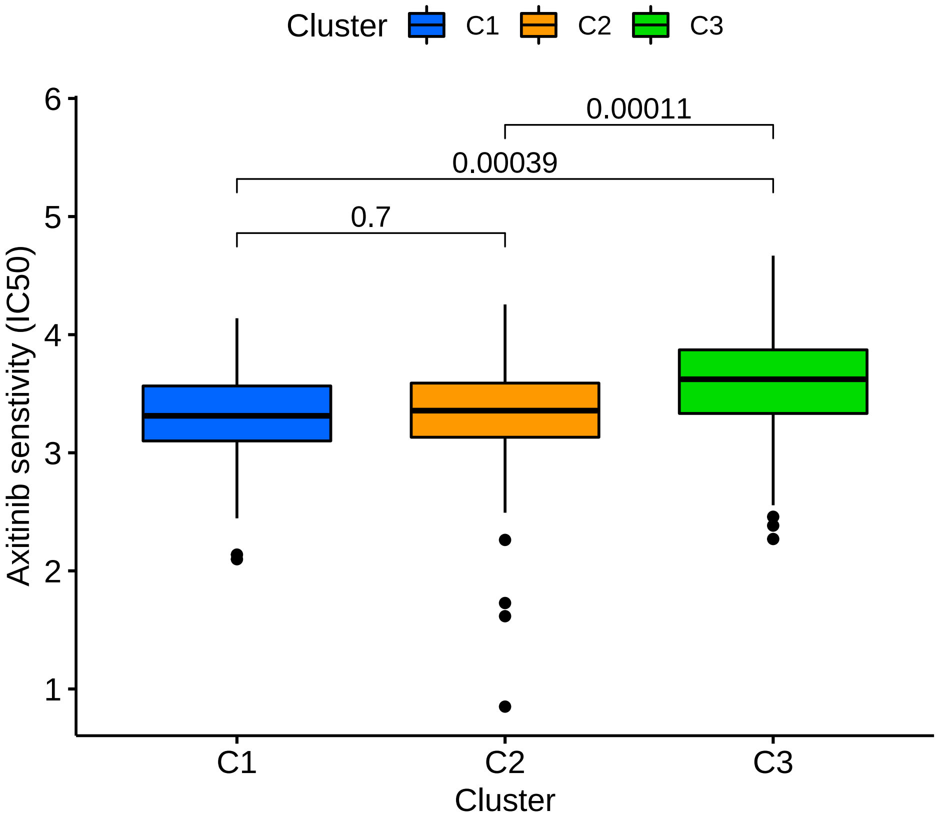

Cluster
C1
C2
C3
0.00011
0.00039
0.7
6
5
4
3
2
1
Axitinib senstivity (IC50)
C1
C2
C3
Cluster

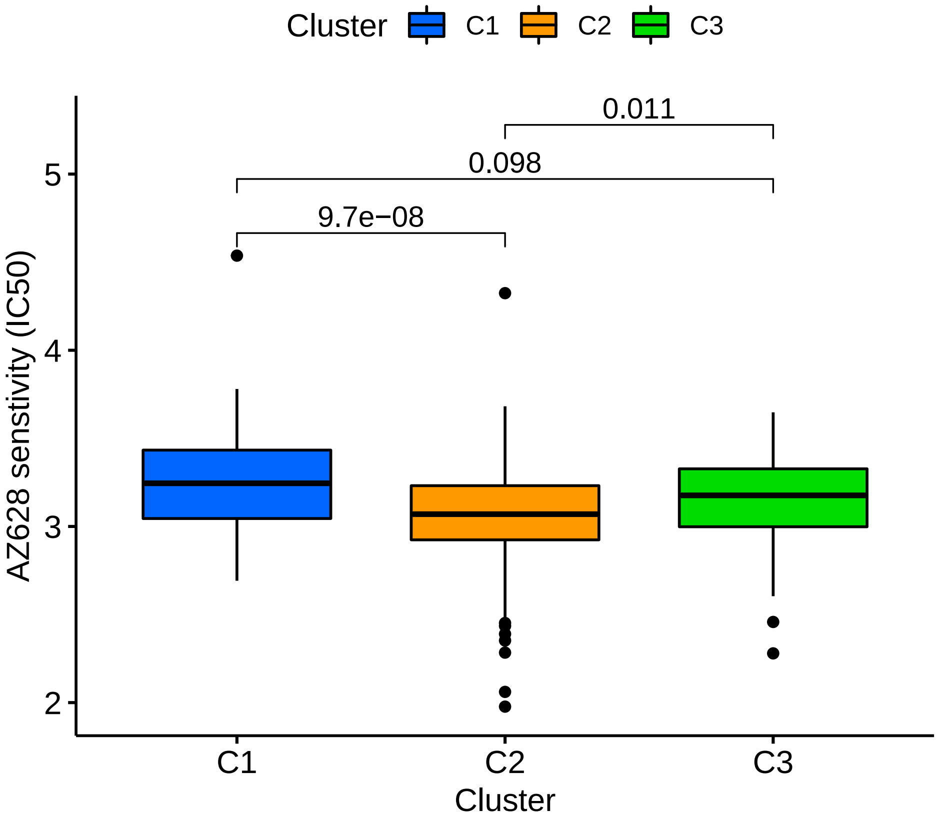
Cluster
C1
C2
C3
0.011
0.098
9.7e−08
5
4
3
2
AZ628 senstivity (IC50)
C1
C2
C3
Cluster

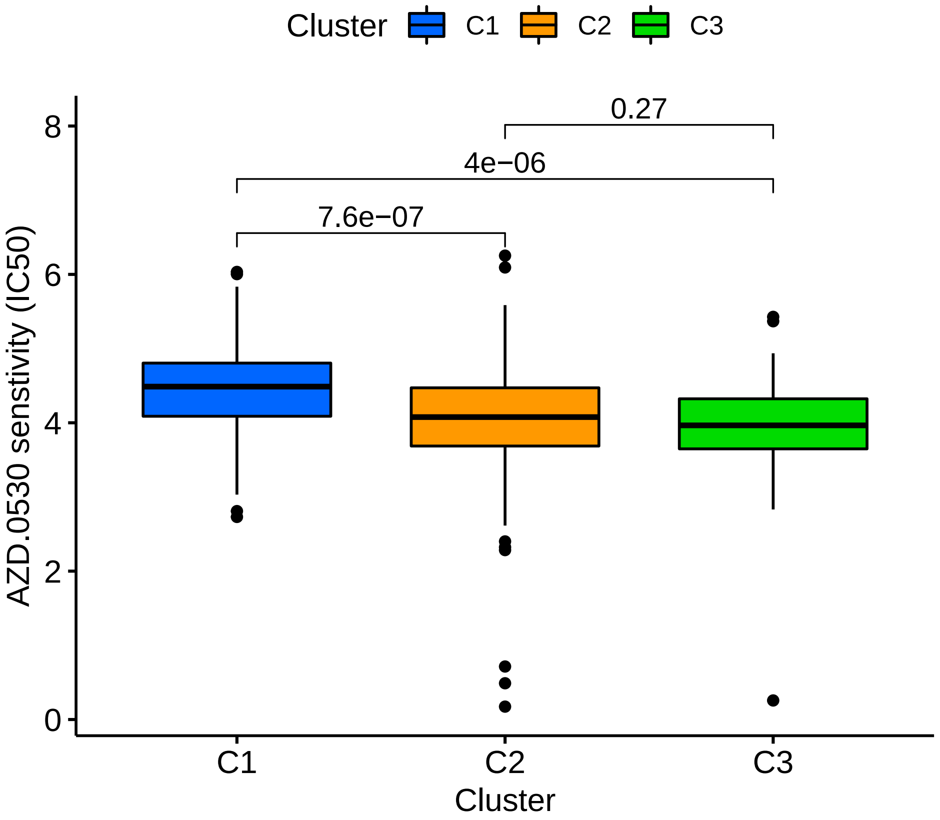

Cluster
C1
C2
C3
0.27
4e−06
7.6e−07
AZD.0530 senstivity (IC50)
8
6
4
2
0
C1
C2
C3
Cluster

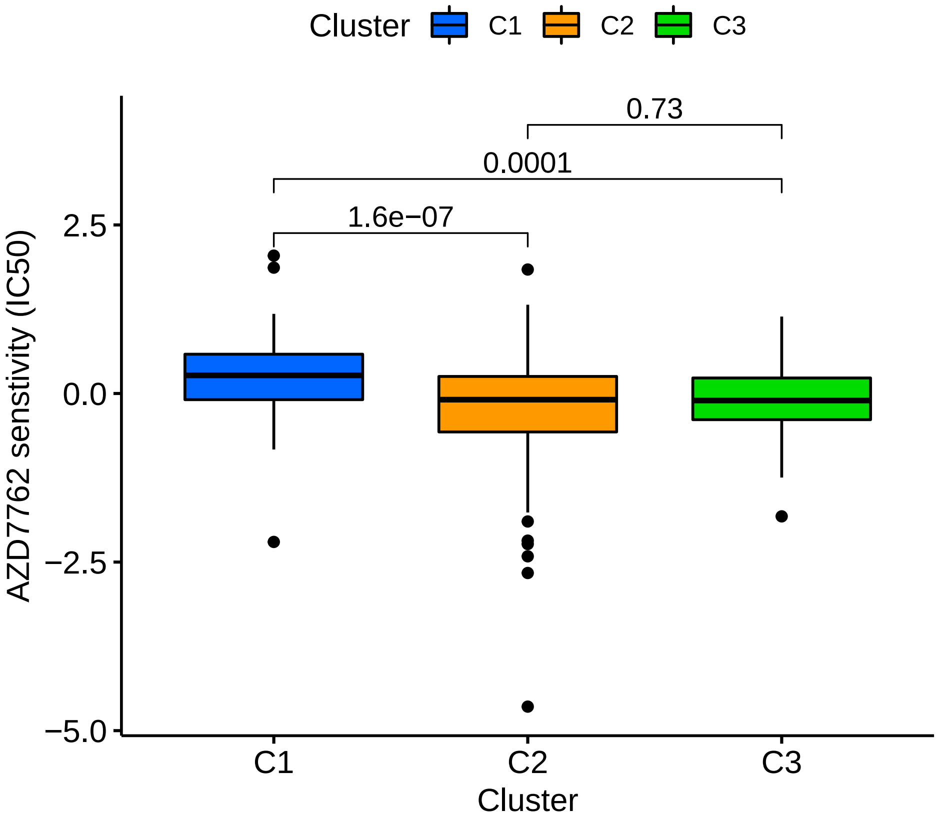

Cluster
C1
C2
C3
0.73
0.0001
1.6e−07
2.5
0.0
−2.5
−5.0
AZD7762 senstivity (IC50)
C1
C2
C3
Cluster

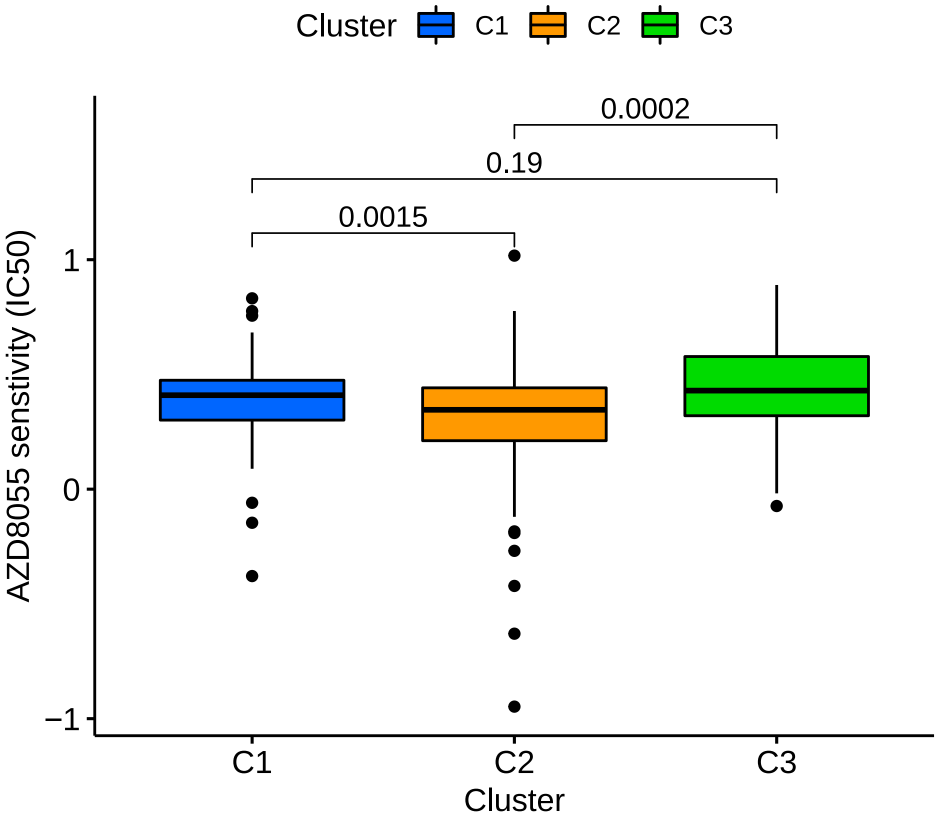
Cluster
C1
C2
C3
0.0002
0.19
0.0015
AZD8055 senstivity (IC50)
1
0
−1
C1
C2
C3
Cluster

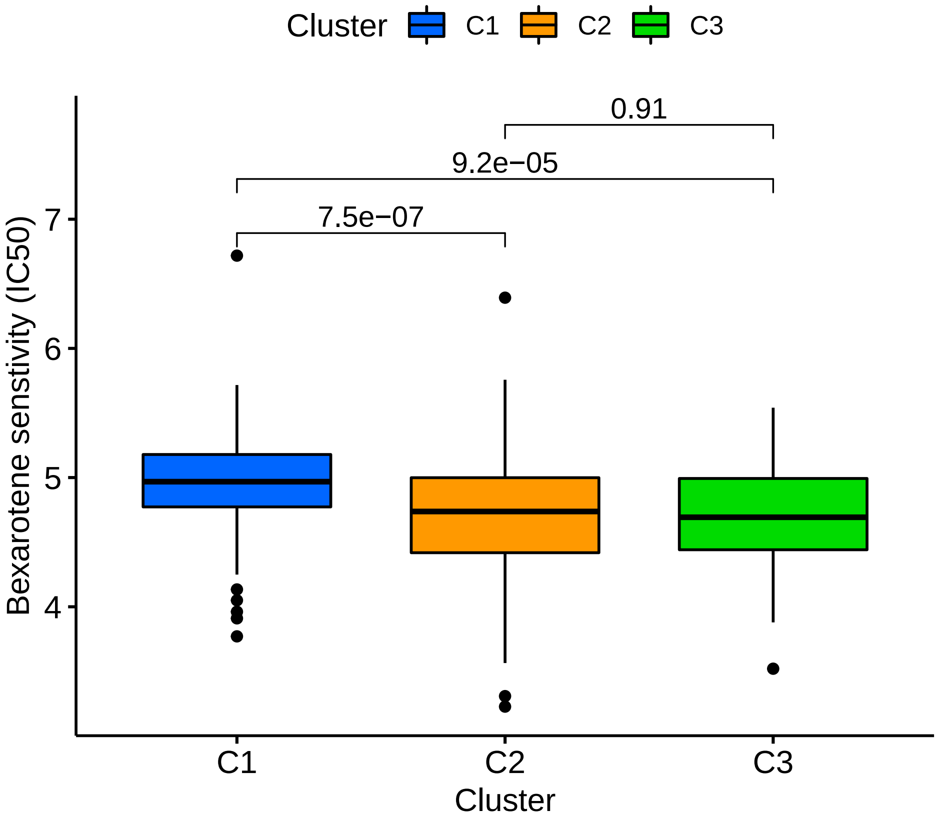
Cluster
C1
C2
C3
0.91
9.2e−05
7.5e−07
Bexarotene senstivity (IC50)
7
6
5
4
C1
C2
C3
Cluster

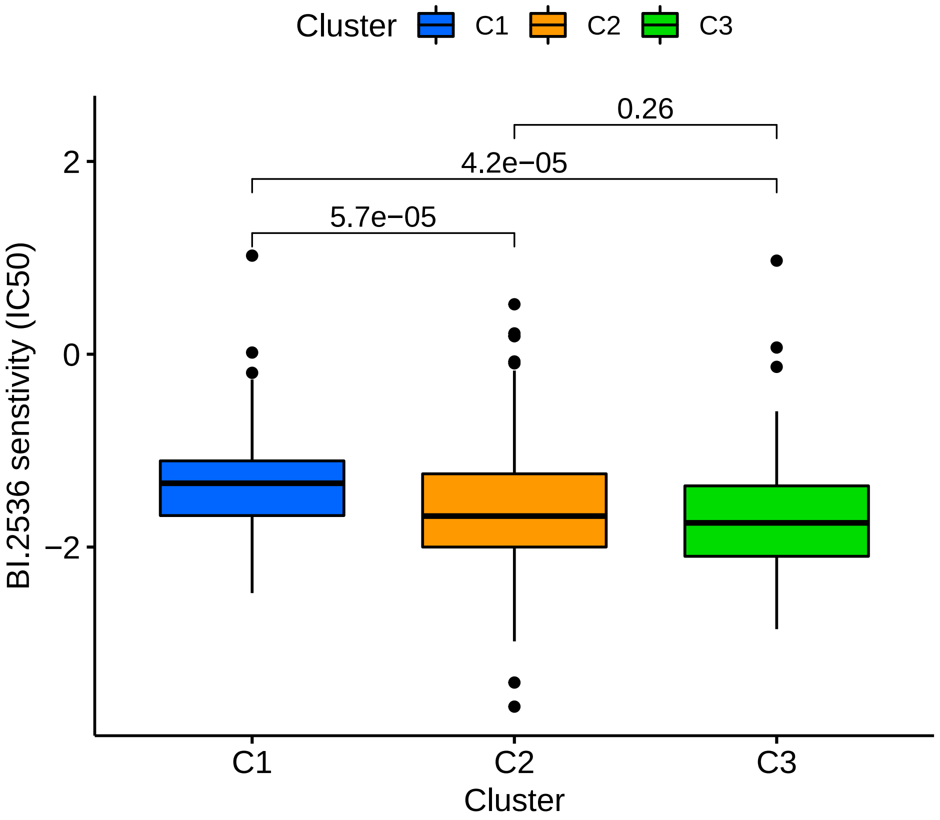
Cluster
C1
C2
C3
0.26
4.2e−05
5.7e−05
2
0
−2
BI.2536 senstivity (IC50)
C1
C2
C3
Cluster

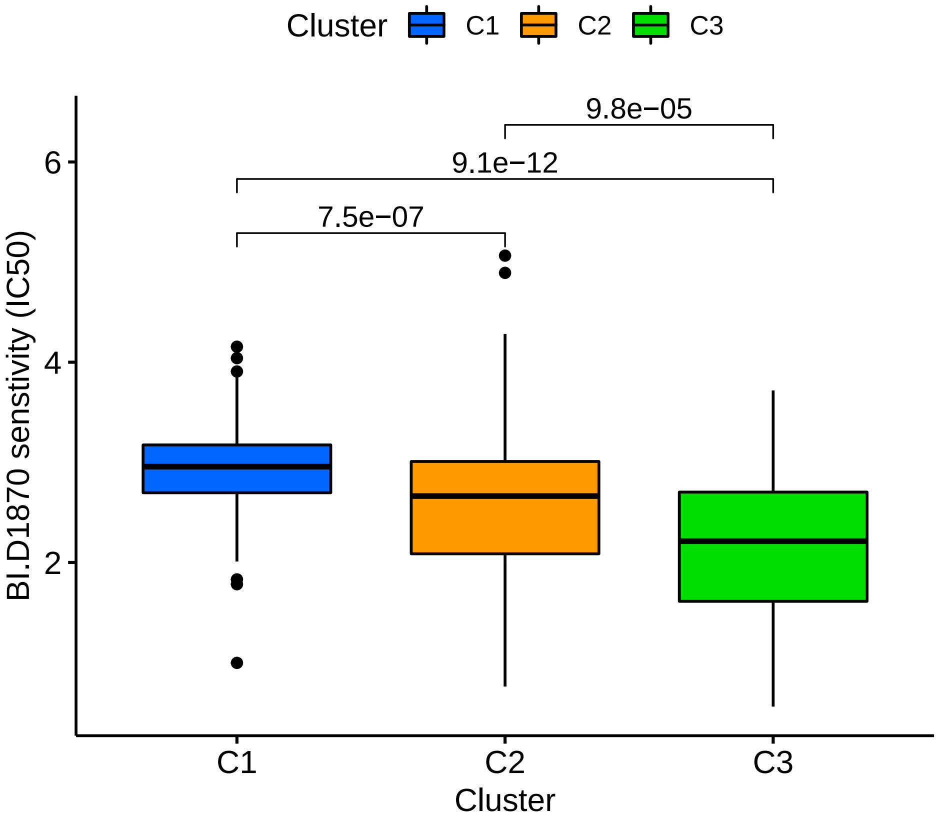
Cluster
C1
C2
C3
9.8e−05
9.1e−12
7.5e−07
6
4
2
BI.D1870 senstivity (IC50)
C1
C2
C3
Cluster

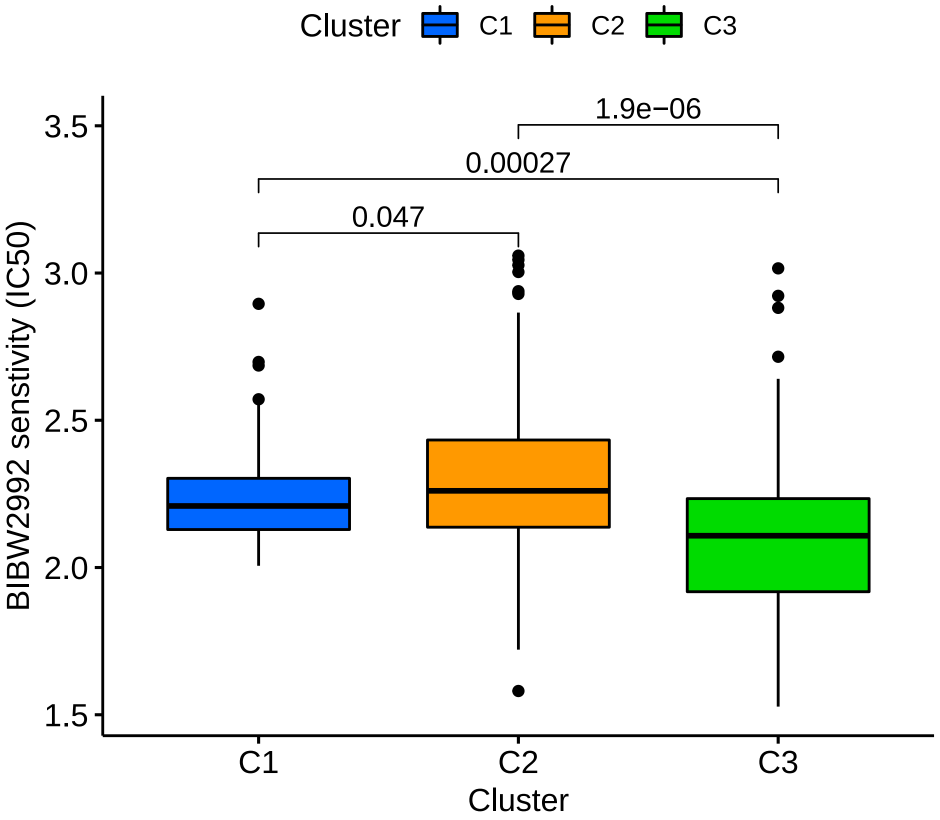
Cluster
C1
C2
C3
1.9e−06
0.00027
0.047
BIBW2992 senstivity (IC50)
3.5
3.0
2.5
2.0
1.5
C1
C2
C3
Cluster

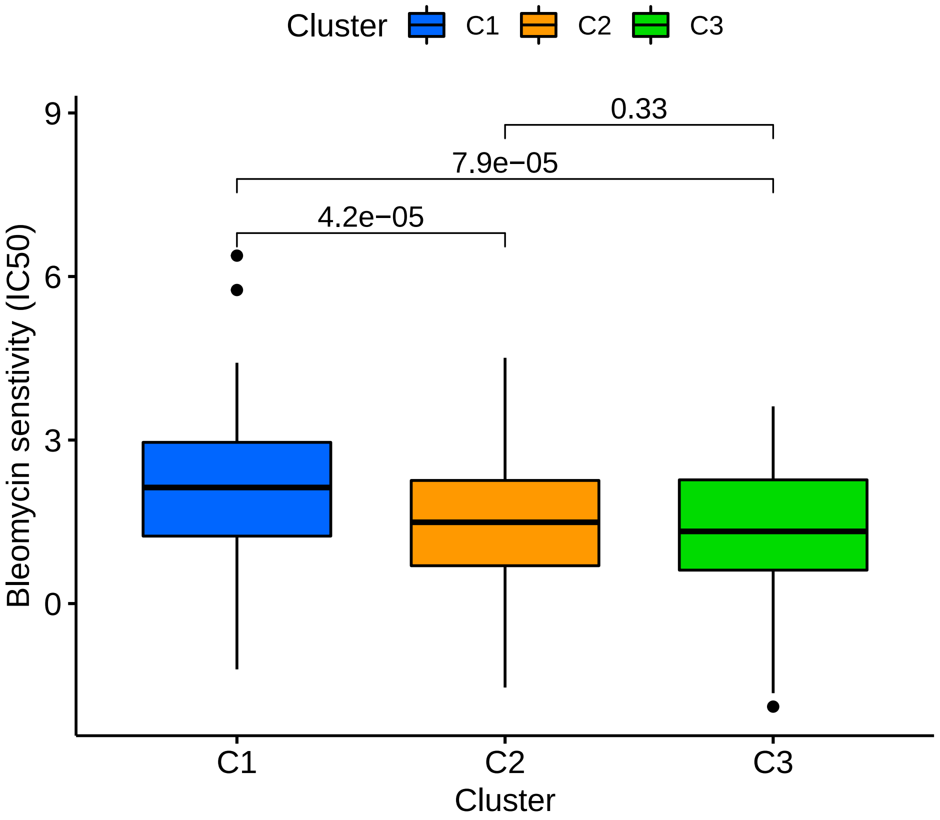
Cluster
C1
C2
C3
0.33
7.9e−05
4.2e−05
Bleomycin senstivity (IC50)
9
6
3
0
C1
C2
C3
Cluster

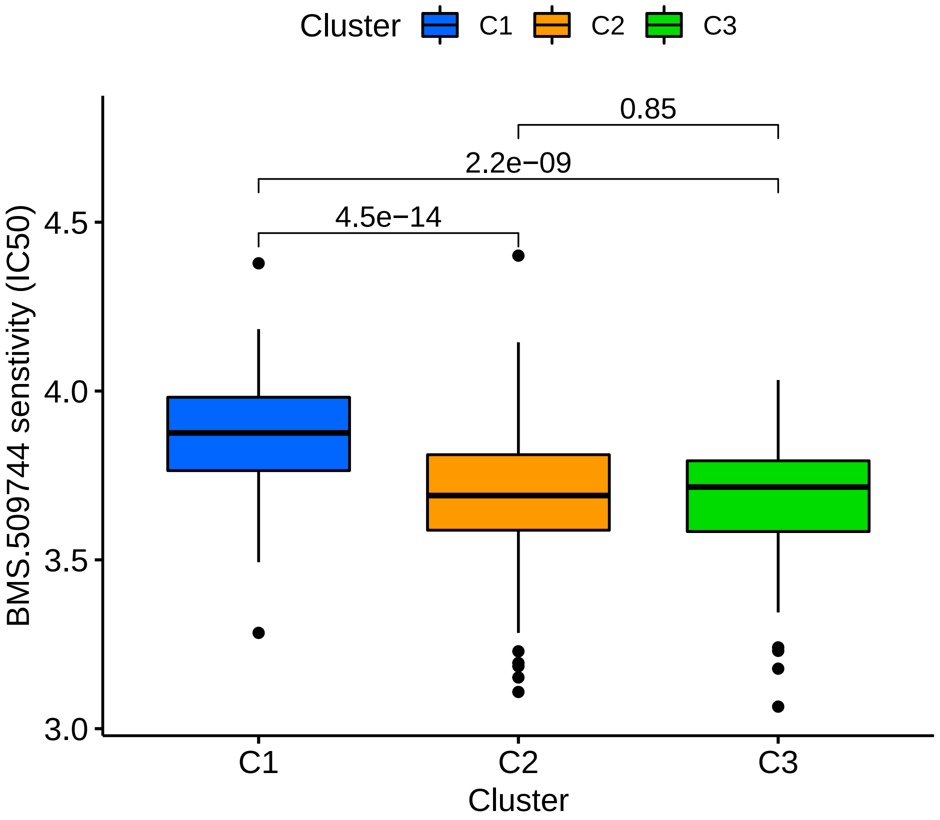

Cluster
C1
C2
C3
0.85
2.2e−09
4.5e−14
BMS.509744 senstivity (IC50)
4.5
4.0
3.5
3.0
C1
C2
C3
Cluster

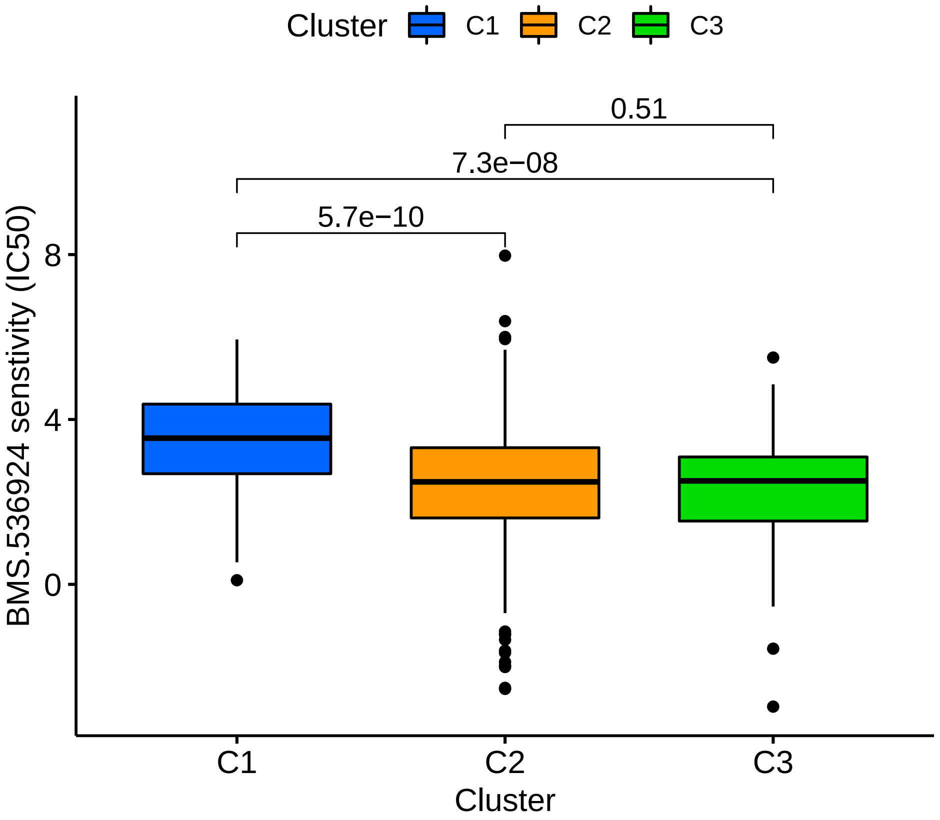

Cluster
C1
C2
C3
0.51
7.3e−08
5.7e−10
BMS.536924 senstivity (IC50)
8
4
0
C1
C2
C3
Cluster

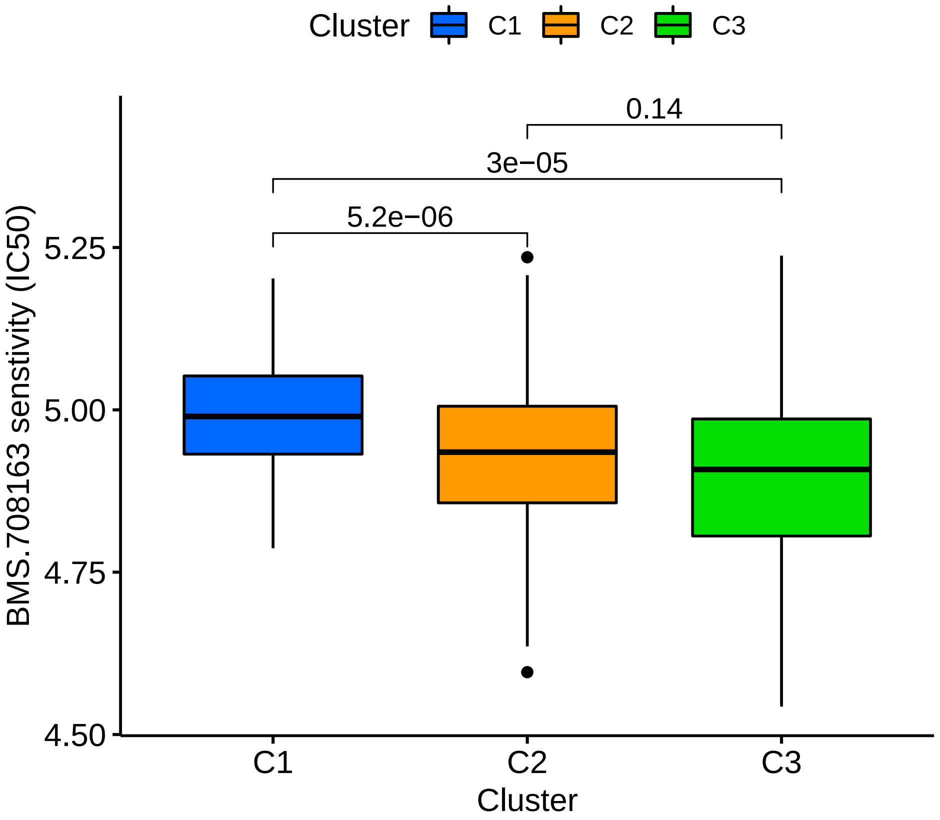

Cluster
C1
C2
C3
0.14
3e−05
5.2e−06
BMS.708163 senstivity (IC50)
5.25
5.00
4.75
4.50
C1
C2
C3
Cluster

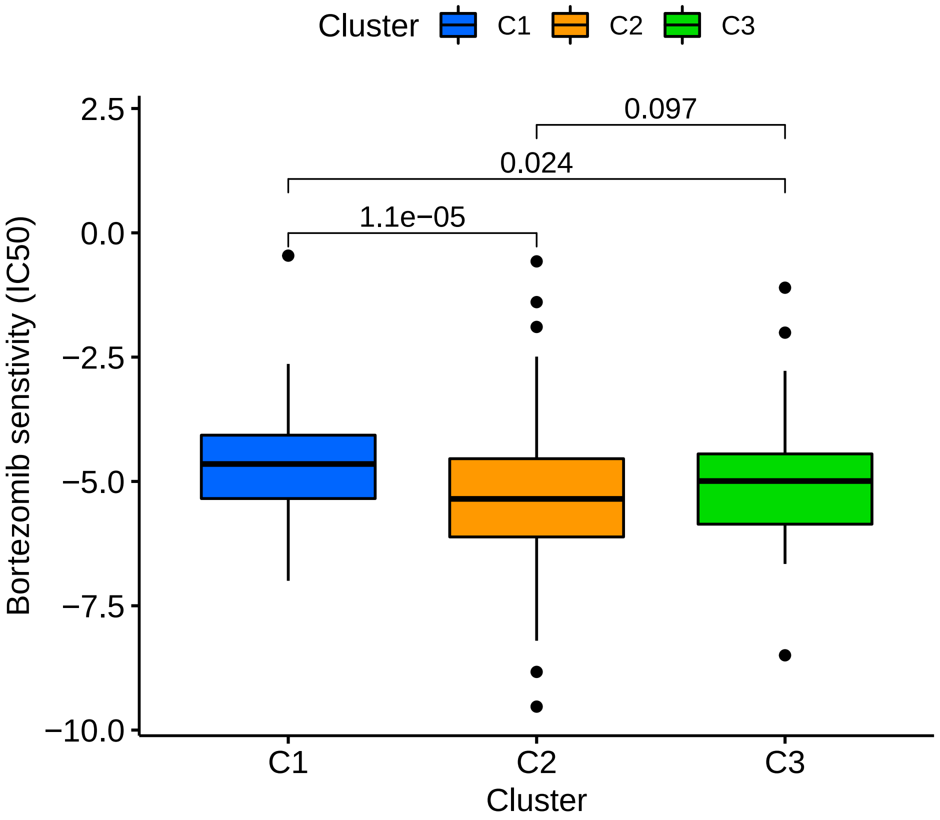
Cluster
C1
C2
C3
Bortezomib senstivity (IC50)
2.5
0.0
−2.5
−5.0
−7.5
−10.0
0.097
0.024
1.1e−05
C1
C2
C3
Cluster

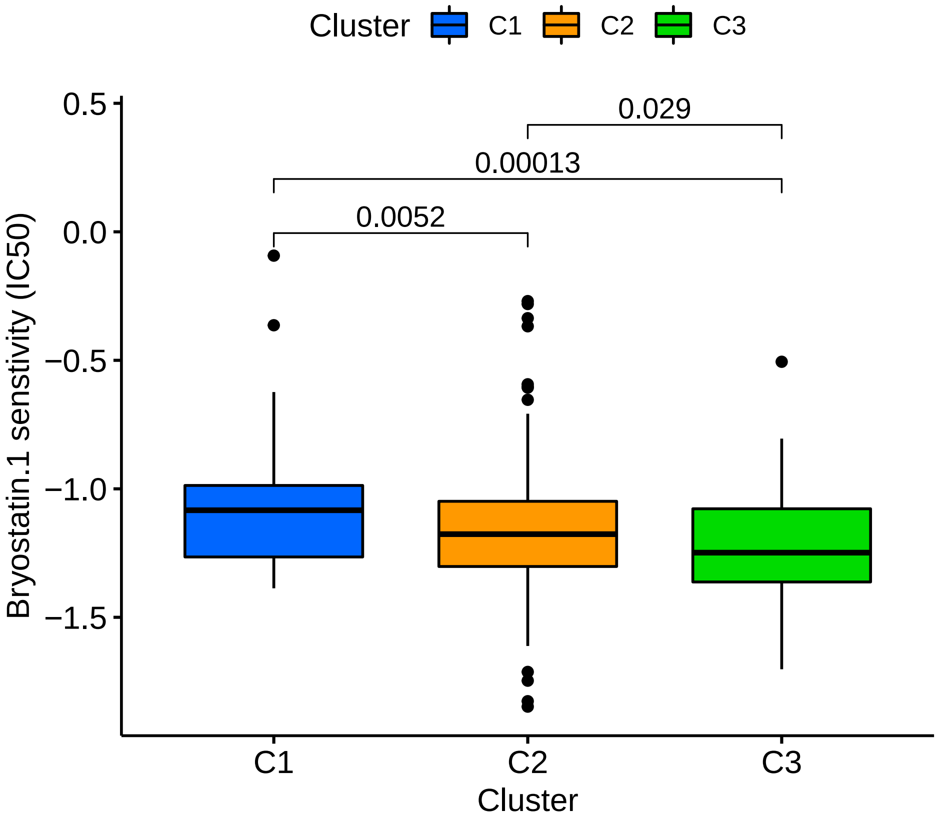

Cluster
C1
C2
C3
0.029
0.00013
0.0052
0.5
0.0
−0.5
−1.0
−1.5
Bryostatin.1 senstivity (IC50)
C1
C2
C3
Cluster

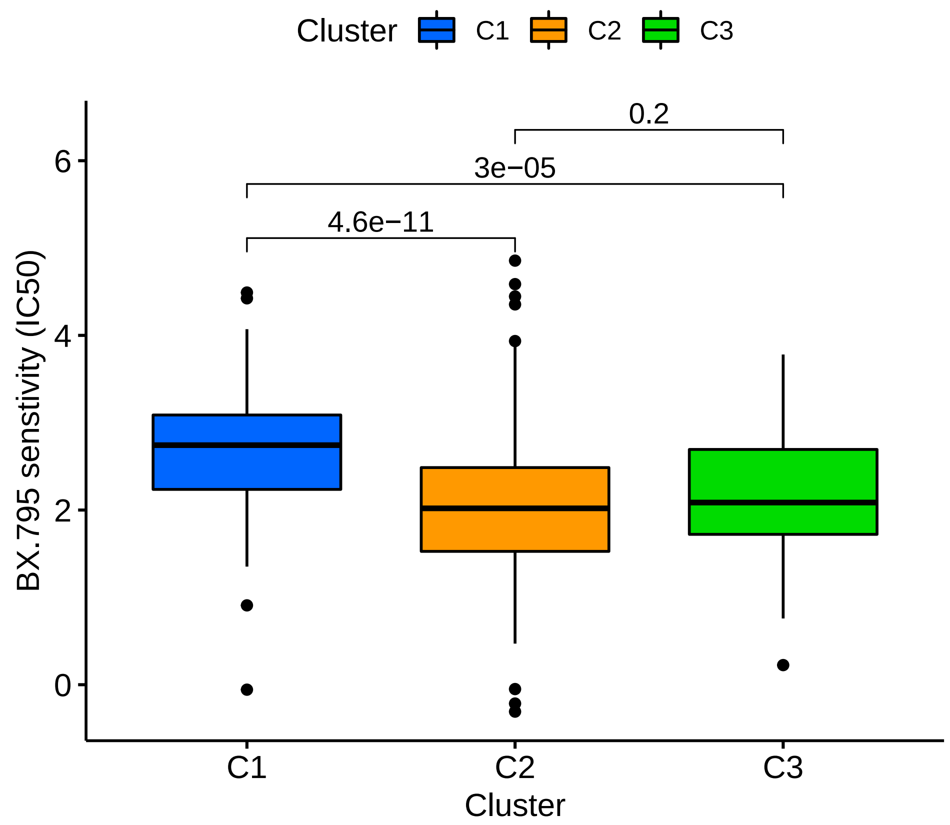

Cluster
C1
C2
C3
0.2
3e−05
4.6e−11
6
4
2
0
BX.795 senstivity (IC50)
C1
C2
C3
Cluster

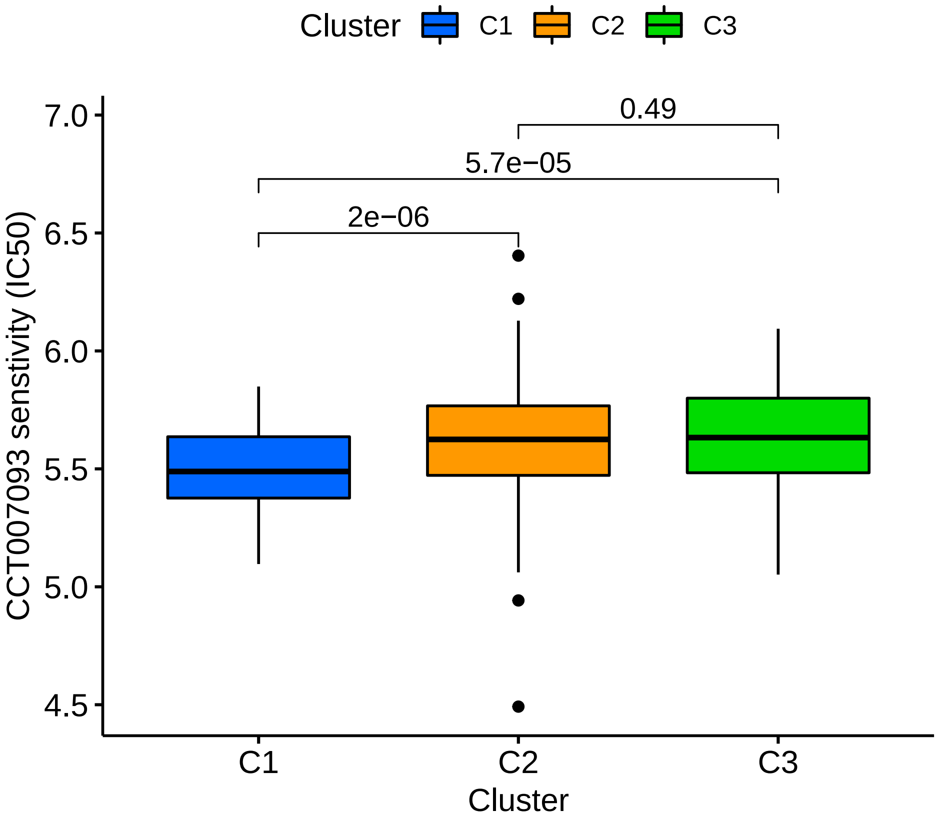
Cluster
C1
C2
C3
CCT007093 senstivity (IC50)
7.0
6.5
6.0
5.5
5.0
4.5
0.49
5.7e−05
2e−06
C1
C2
C3
Cluster

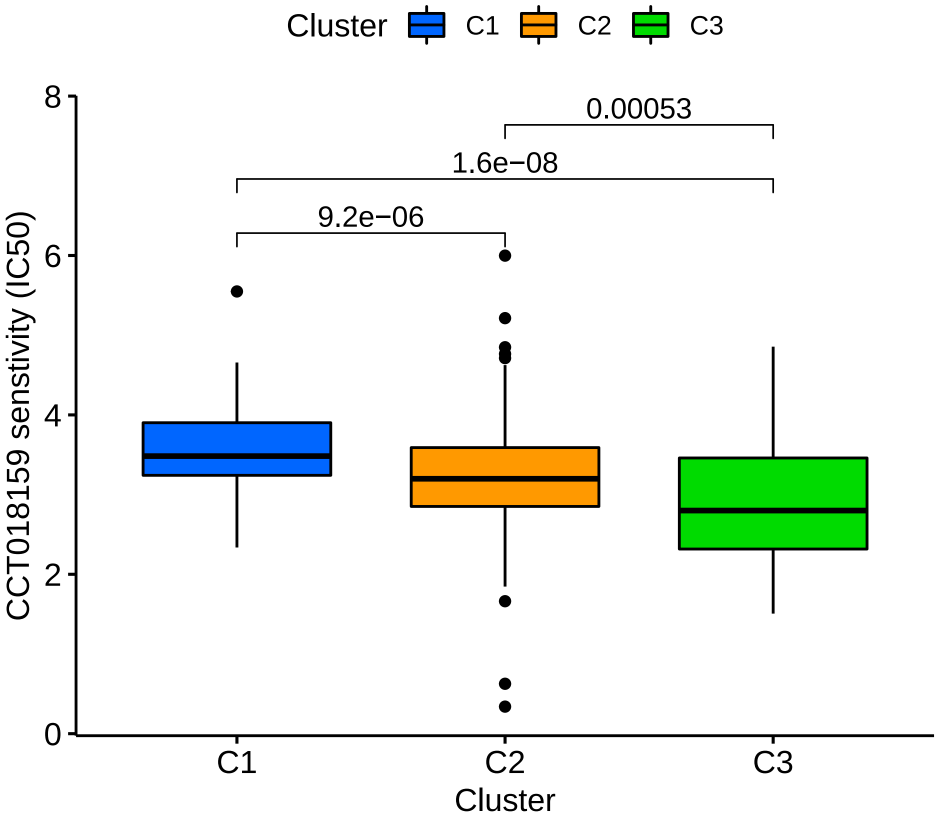

Cluster
C1
C2
C3
0.00053
1.6e−08
9.2e−06
8
6
4
2
0
CCT018159 senstivity (IC50)
C1
C2
C3
Cluster

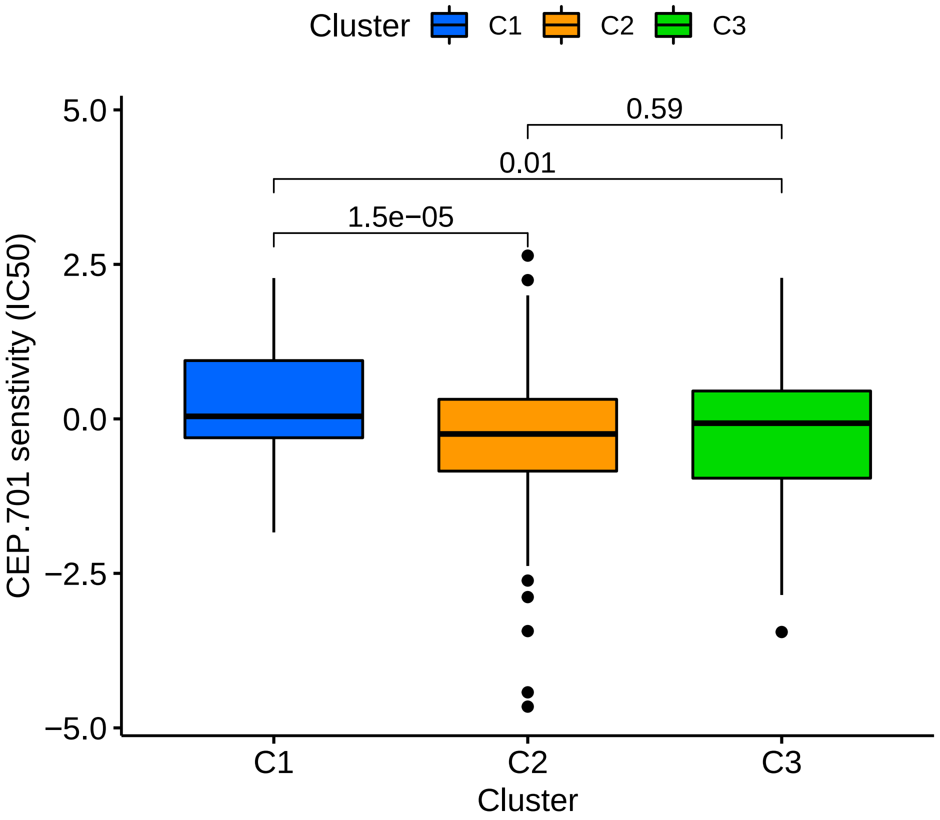

Cluster
C1
C2
C3
0.59
0.01
1.5e−05
5.0
2.5
0.0
−2.5
−5.0
CEP.701 senstivity (IC50)
Cluster

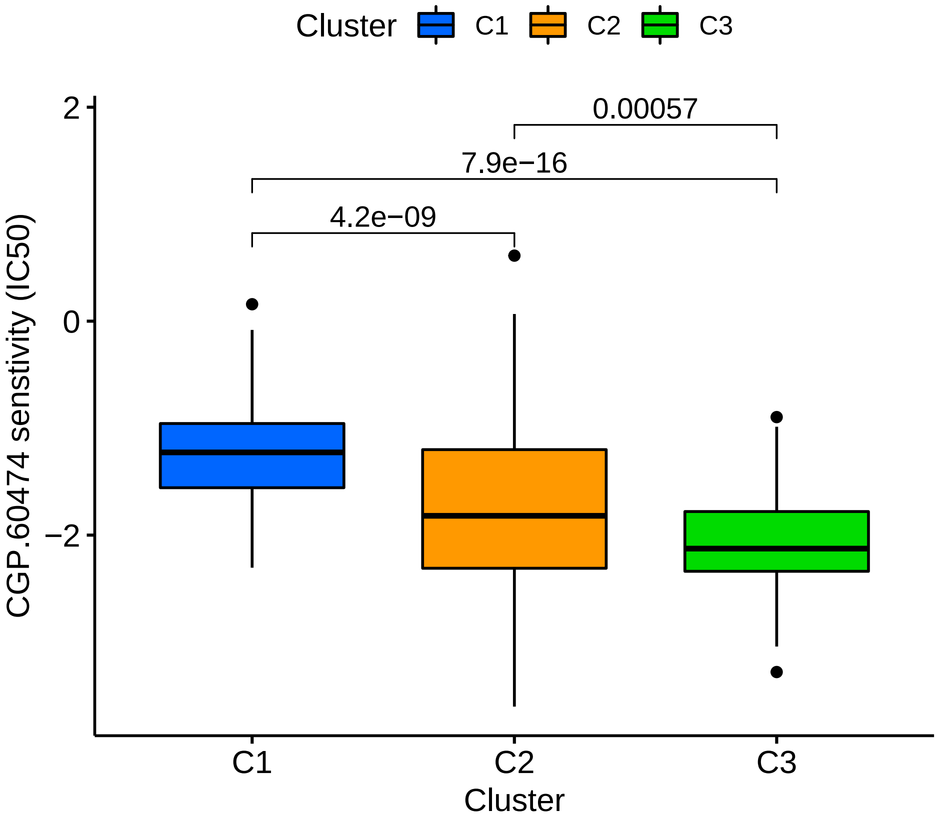
Cluster
C1
C2
C3
0.00057
7.9e−16
4.2e−09
CGP.60474 senstivity (IC50)
2
0
−2
C1
C2
C3
Cluster

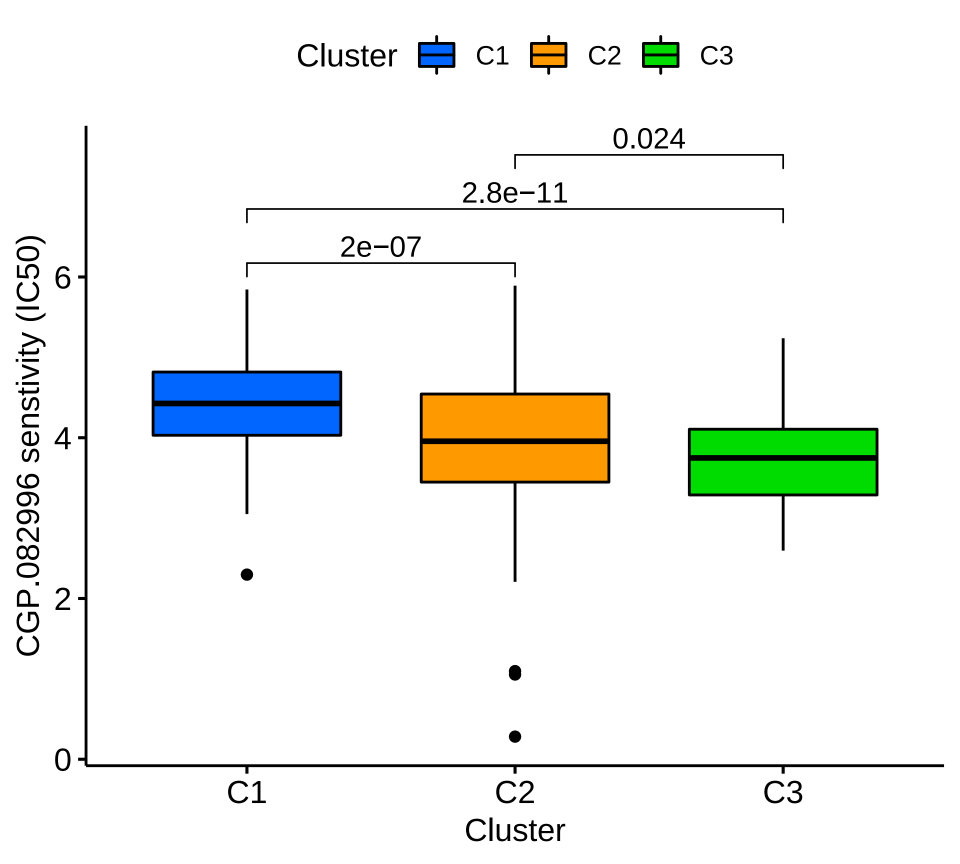
Cluster
C1
C2
C3
0.024
2.8e−11
2e−07
CGP.082996 senstivity (IC50)
6
4
2
0
C1
C2
C3
Cluster

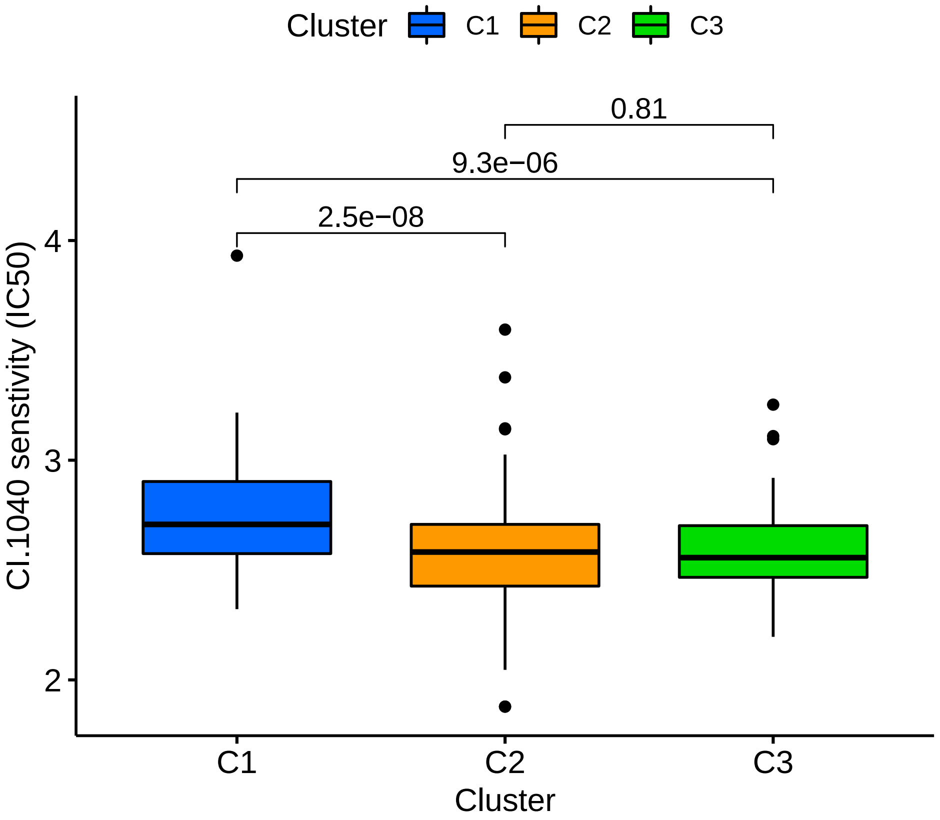
Cluster
C1
C2
C3
0.81
9.3e−06
2.5e−08
4
3
2
CI.1040 senstivity (IC50)
C1
C2
C3
Cluster

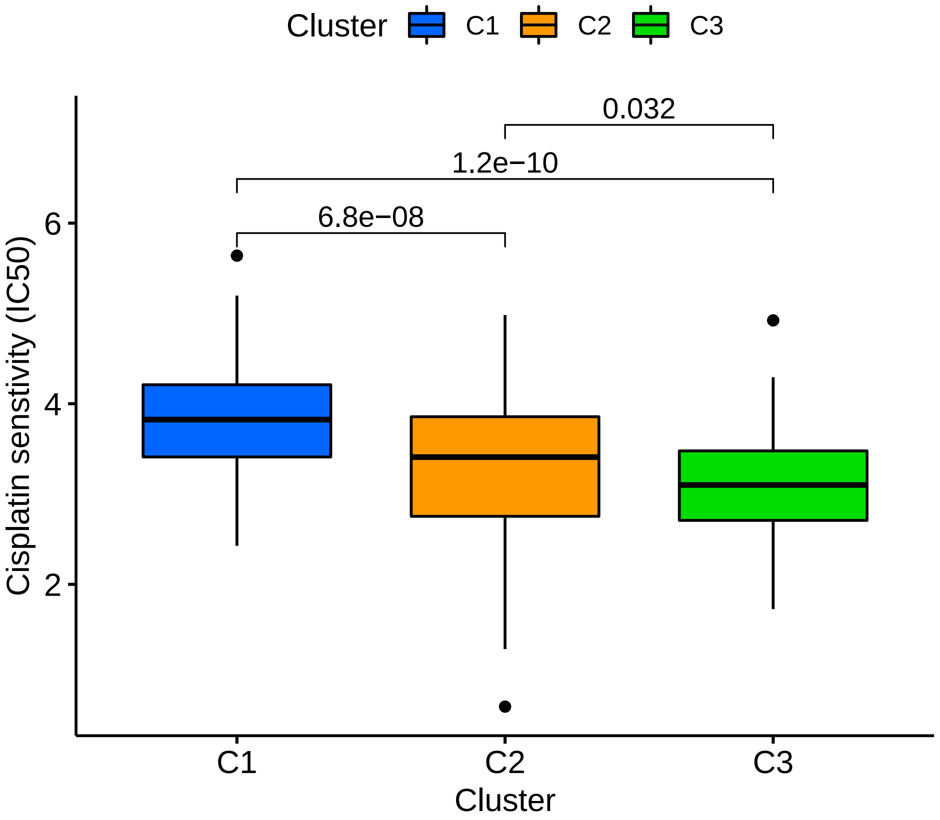
Cluster
C1
C2
C3
0.032
1.2e−10
6.8e−08
Cisplatin senstivity (IC50)
6
4
2
C1
C2
C3
Cluster

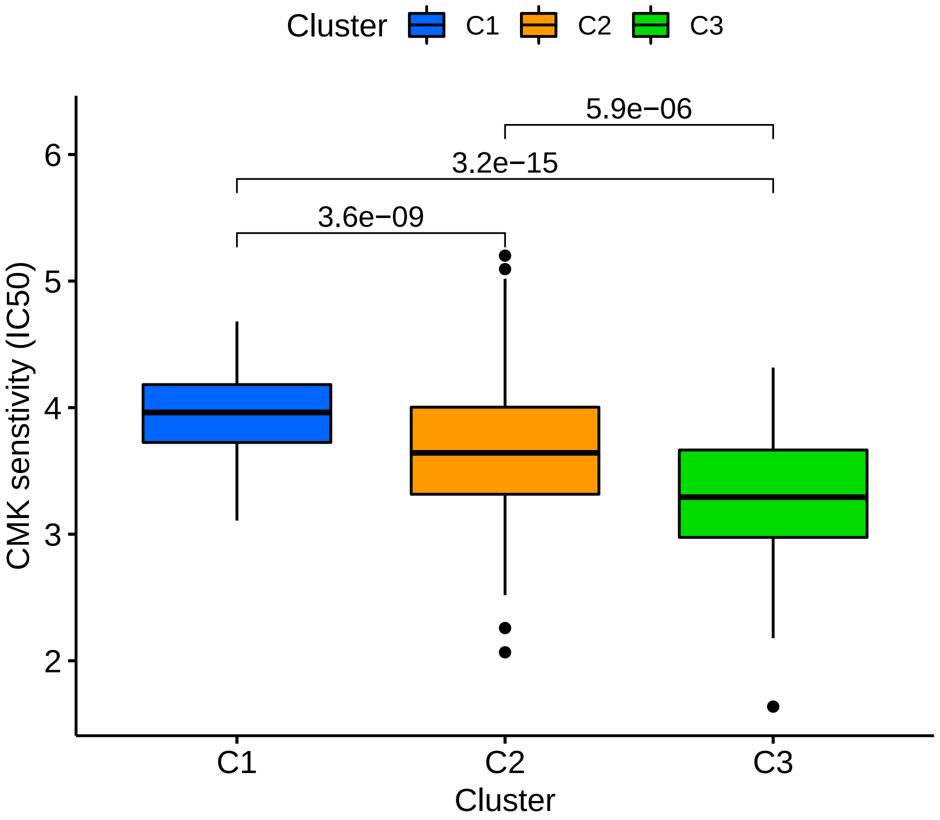

Cluster
C1
C2
C3
5.9e−06
3.2e−15
3.6e−09
6
5
4
3
2
CMK senstivity (IC50)
C1
C2
C3
Cluster

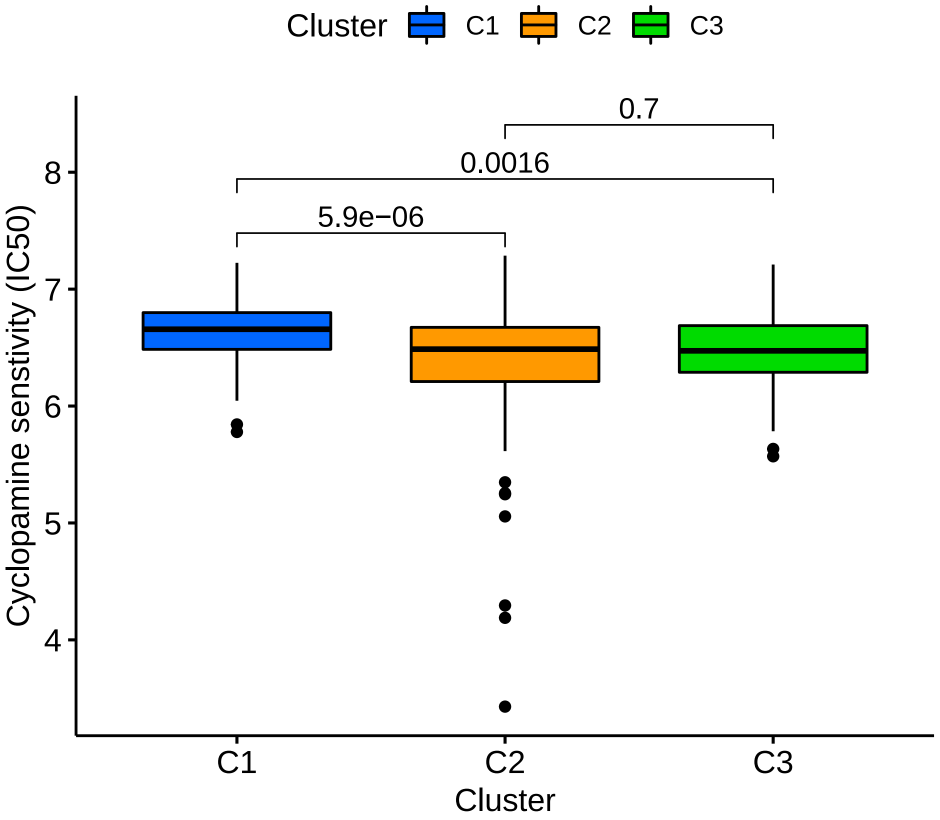
Cluster
C1
C2
C3
0.7
0.0016
5.9e−06
Cyclopamine senstivity (IC50)
8
7
6
5
4
C1
C2
C3
Cluster

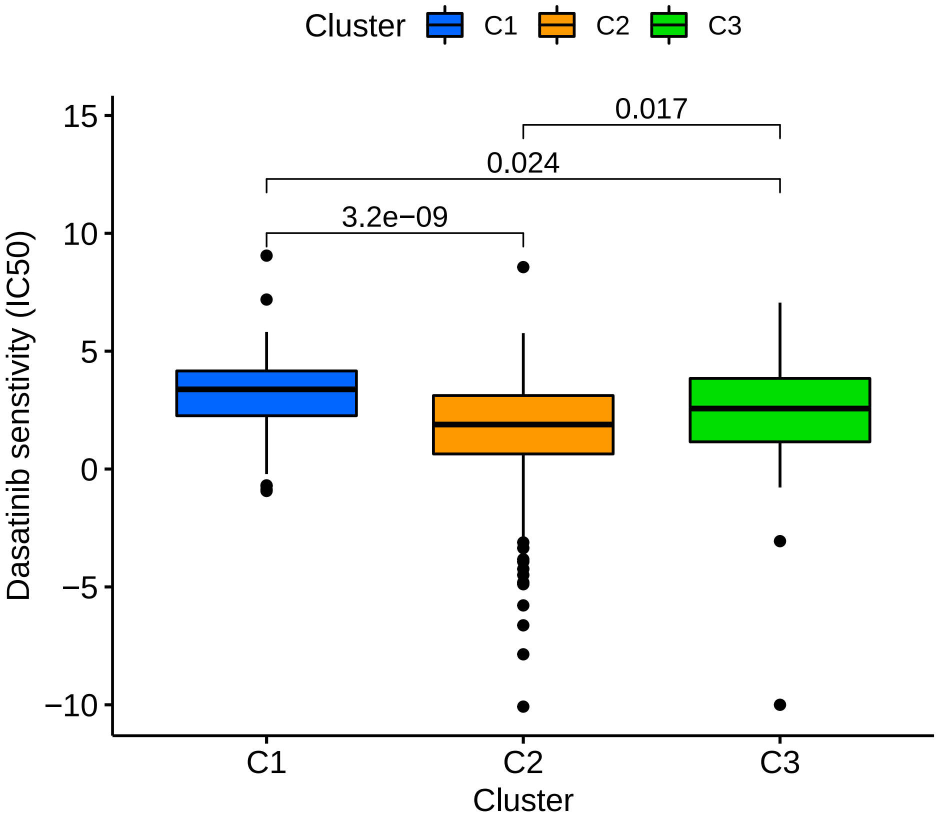
Cluster
C1
C2
C3
0.017
0.024
3.2e−09
15
10
5
0
−5
−10
Dasatinib senstivity (IC50)
C1
C2
C3
Cluster

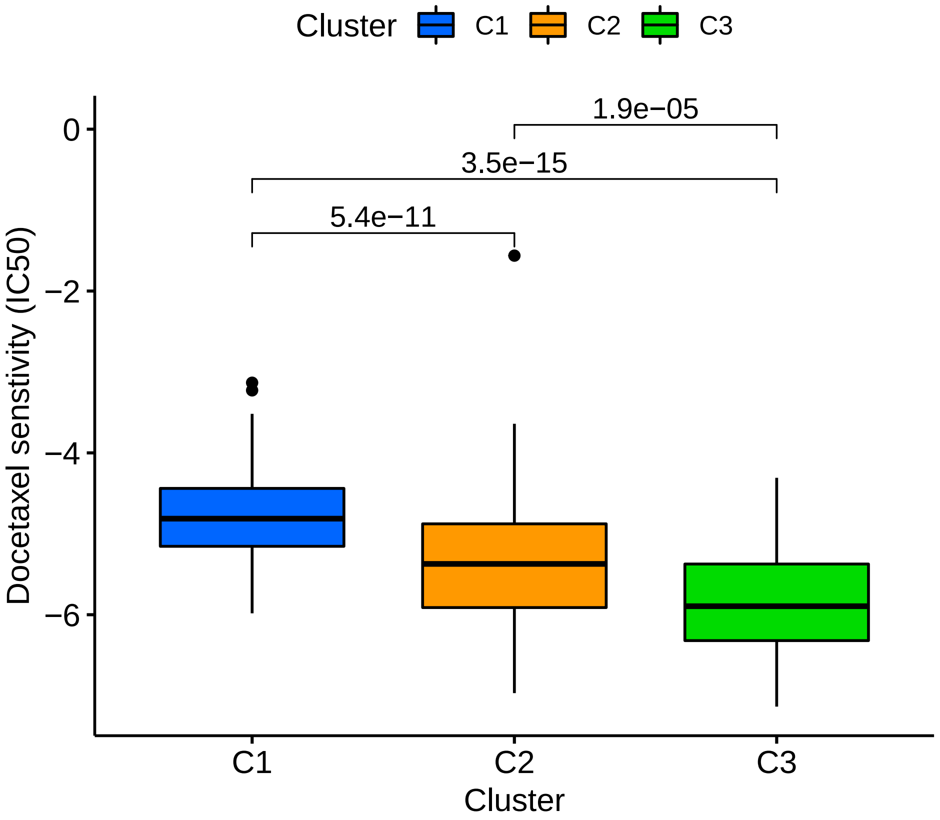

Cluster
C1
C2
C3
1.9e−05
3.5e−15
5.4e−11
0
−2
−4
−6
Docetaxel senstivity (IC50)
C1
C2
C3
Cluster

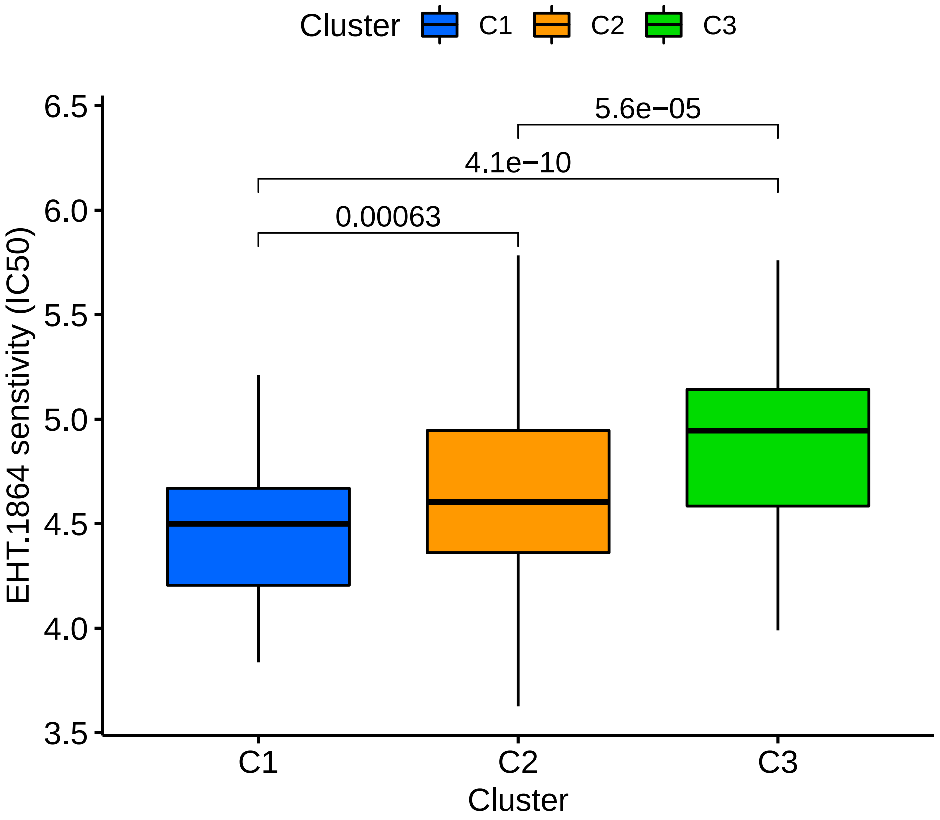

Cluster
C1
C2
C3
EHT.1864 senstivity (IC50)
6.5
6.0
5.5
5.0
4.5
4.0
3.5
5.6e−05
4.1e−10
0.00063
C1
C2
C3
Cluster

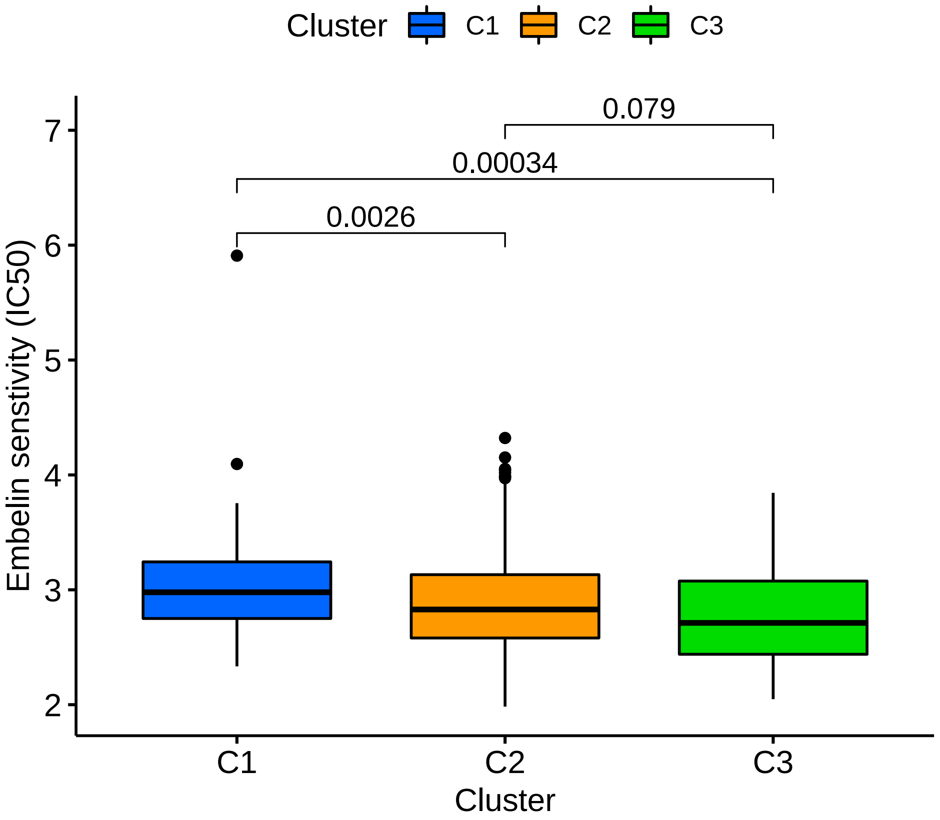

Cluster
C1
C2
C3
0.079
0.00034
0.0026
Embelin senstivity (IC50)
7
6
5
4
3
2
C1
C2
C3
Cluster

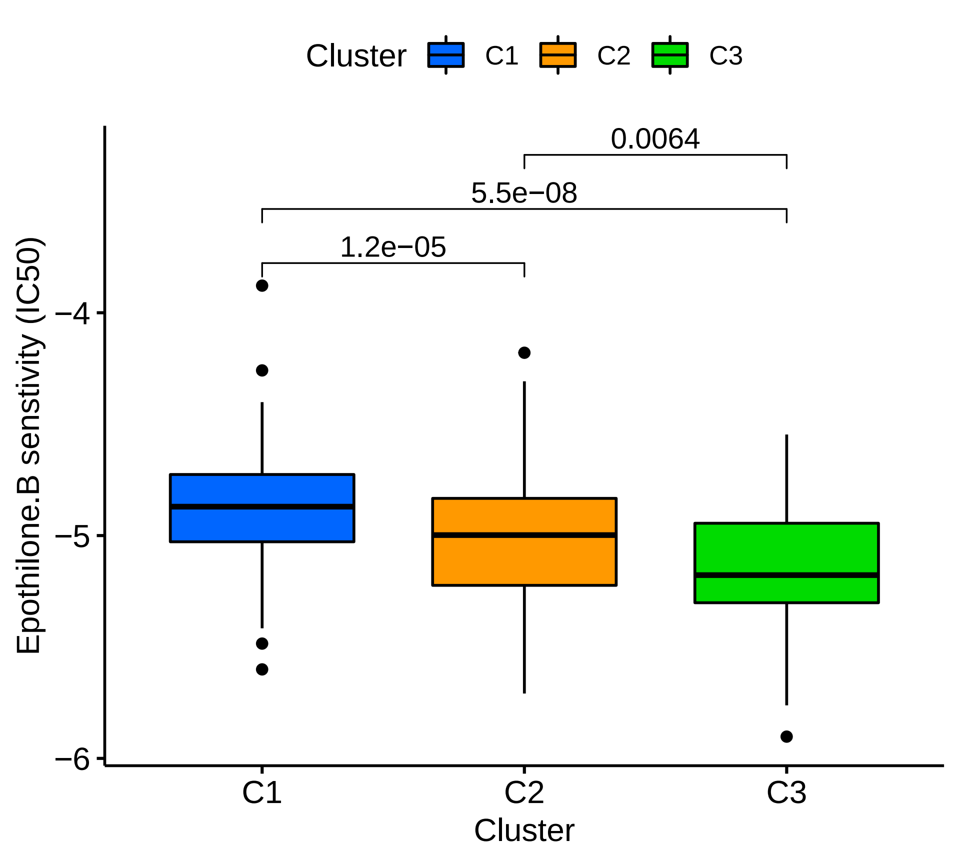

Cluster
C1
C2
C3
0.0064
5.5e−08
1.2e−05
−4
−5
−6
Epothilone.B senstivity (IC50)
C1
C2
C3
Cluster

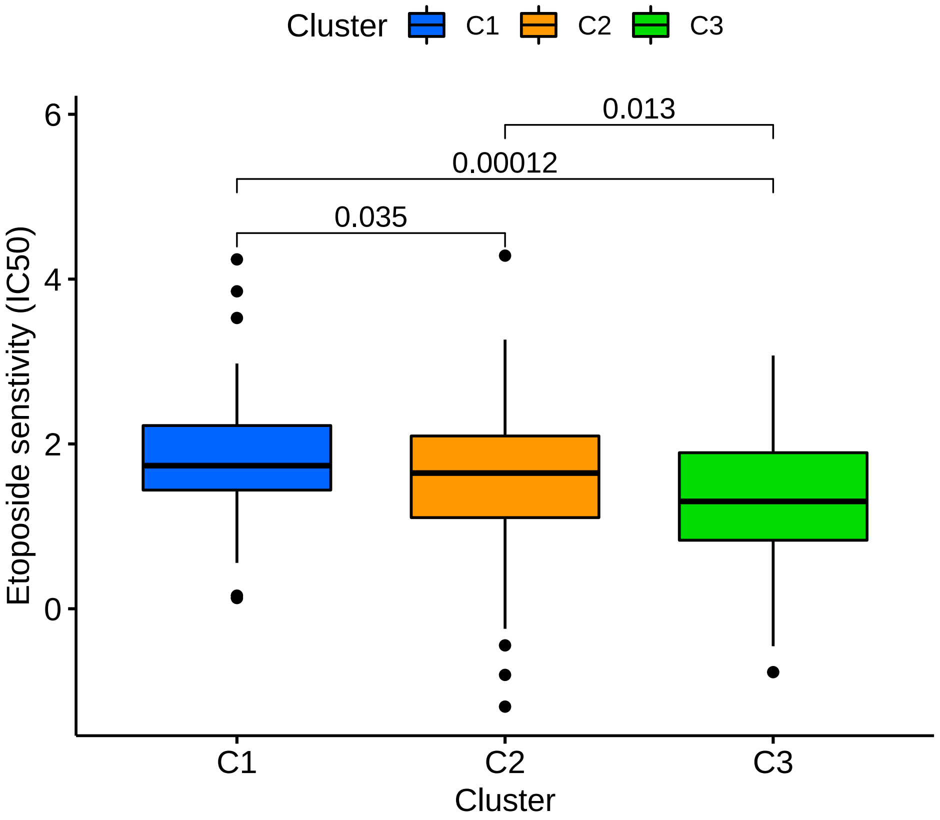

Cluster
C1
C2
C3
0.013
0.00012
0.035
Etoposide senstivity (IC50)
6
4
2
0
C1
C2
C3
Cluster

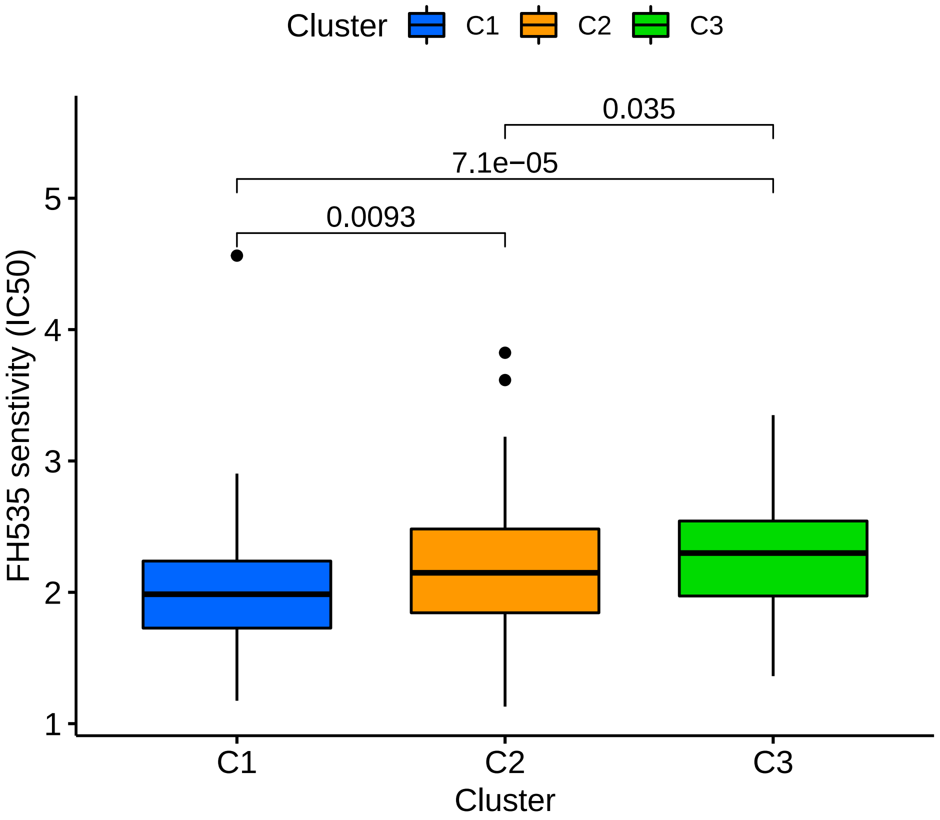
Cluster
C1
C2
C3
0.035
7.1e−05
0.0093
FH535 senstivity (IC50)
5
4
3
2
1
C1
C2
C3
Cluster

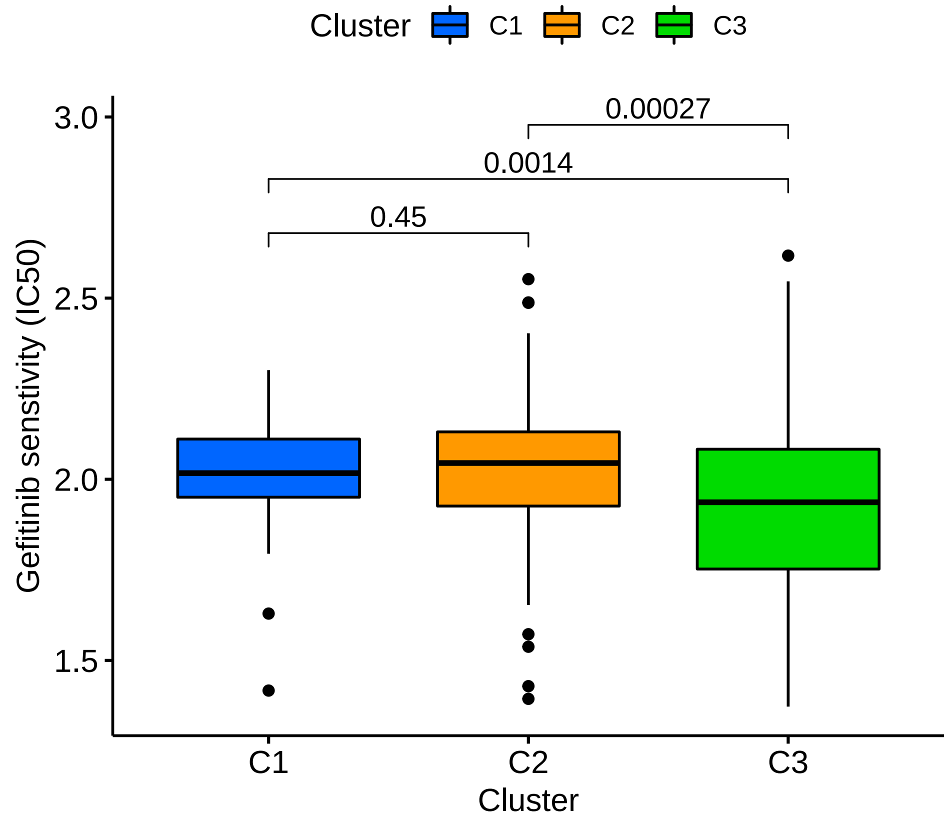

Cluster
C1
C2
C3
0.00027
0.0014
0.45
3.0
2.5
2.0
1.5
Gefitinib senstivity (IC50)
C1
C2
C3
Cluster

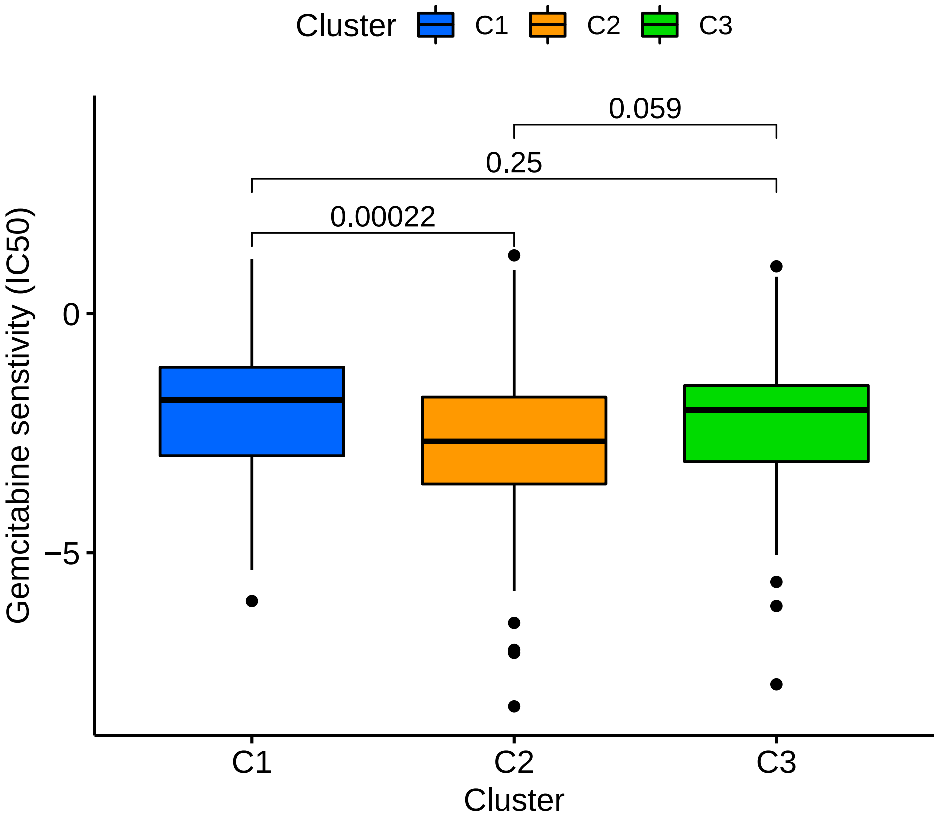

Cluster
C1
C2
C3
0.059
0.25
0.00022
Gemcitabine senstivity (IC50)
0
−5
C1
C2
C3
Cluster

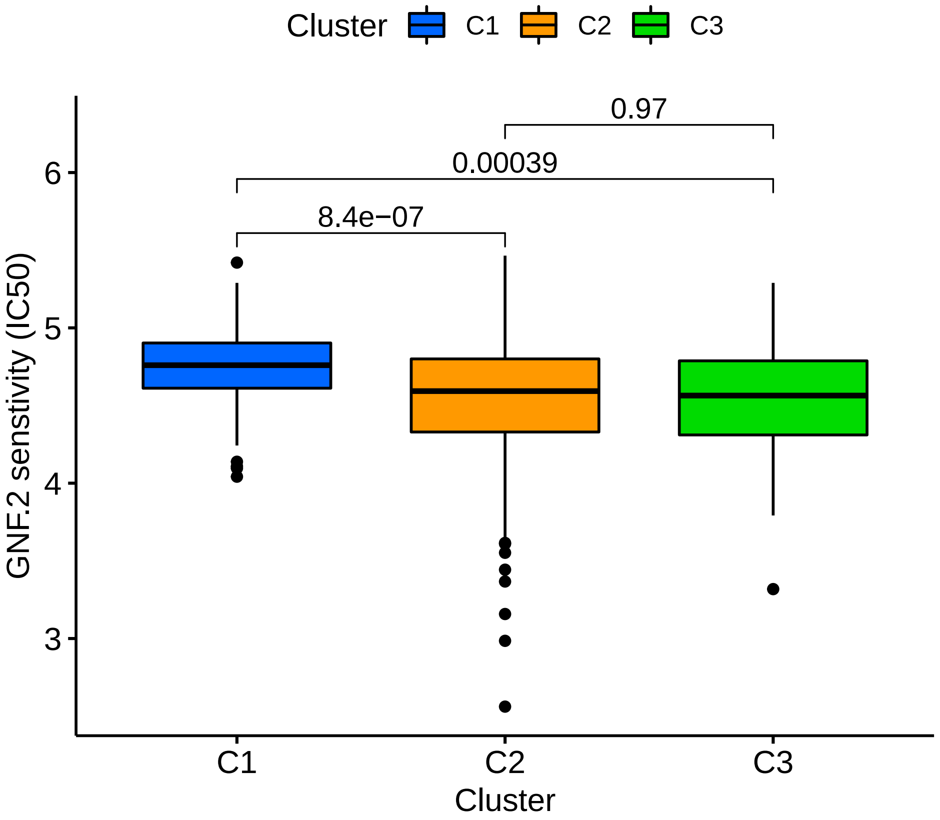

Cluster
C1
C2
C3
0.97
0.00039
8.4e−07
GNF.2 senstivity (IC50)
6
5
4
3
C1
C2
C3
Cluster

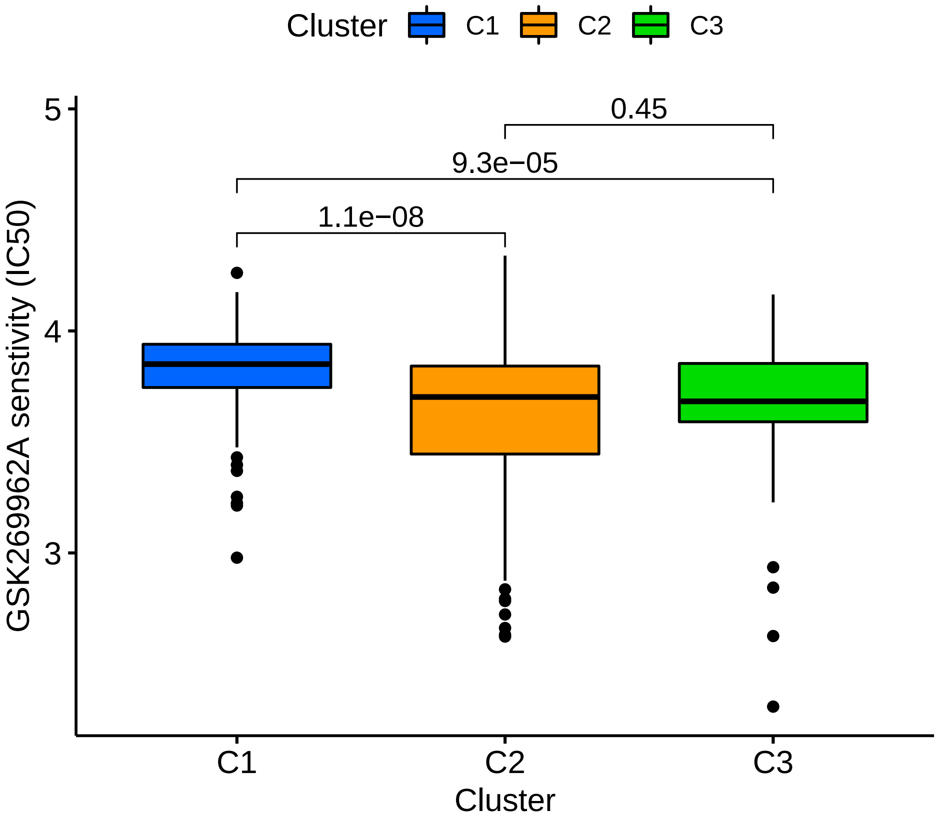

Cluster
C1
C2
C3
GSK269962A senstivity (IC50)
5
4
3
0.45
9.3e−05
1.1e−08
C1
C2
C3
Cluster

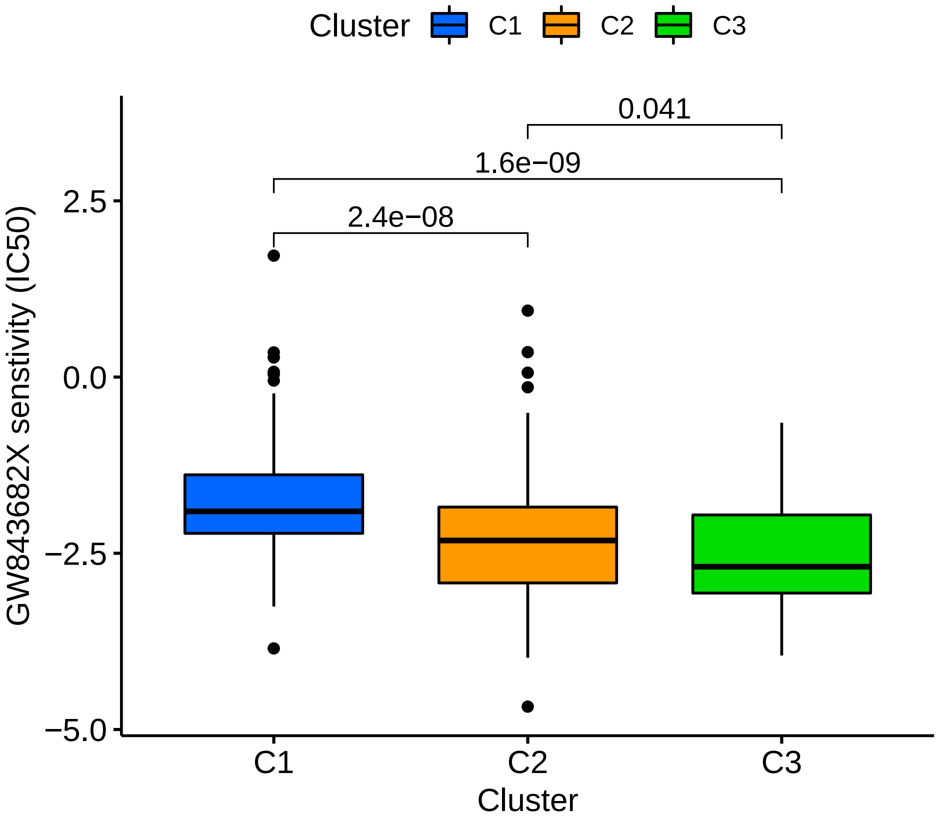
Cluster
C1
C2
C3
0.041
1.6e−09
2.4e−08
2.5
0.0
−2.5
−5.0
GW843682X senstivity (IC50)
C1
C2
C3
Cluster

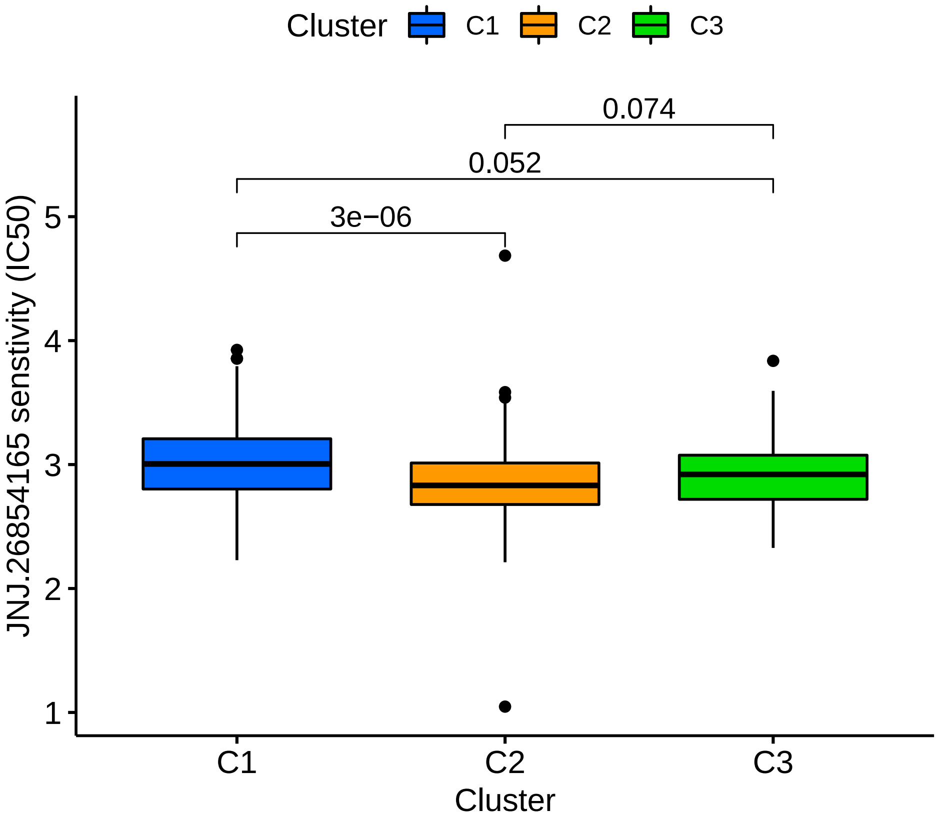

Cluster
C1
C2
C3
0.074
0.052
3e−06
JNJ.26854165 senstivity (IC50)
5
4
3
2
1
C1
C2
C3
Cluster

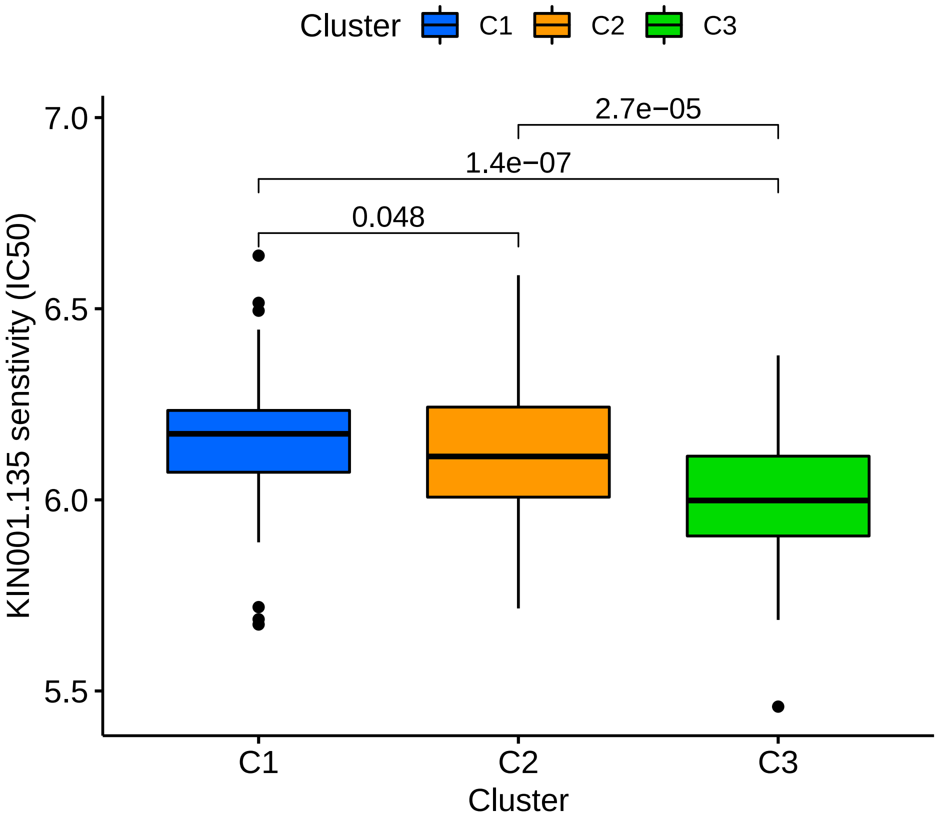

Cluster
C1
C2
C3
2.7e−05
1.4e−07
0.048
7.0
6.5
6.0
5.5
KIN001.135 senstivity (IC50)
C1
C2
C3
Cluster

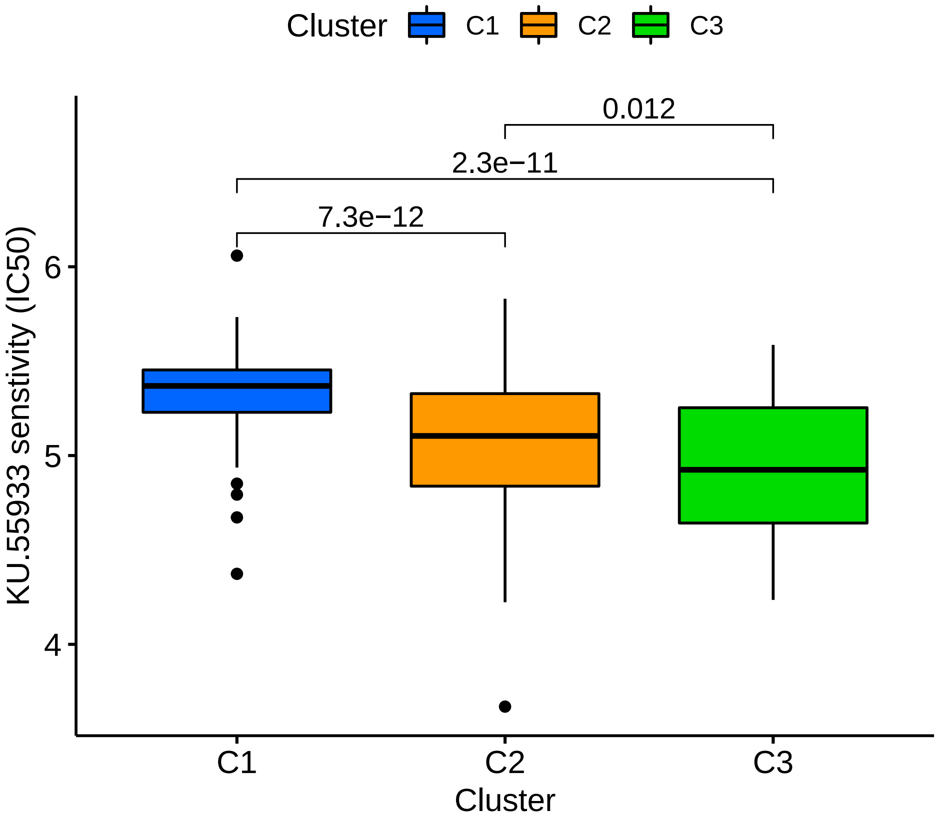

Cluster
C1
C2
C3
0.012
2.3e−11
7.3e−12
KU.55933 senstivity (IC50)
6
5
4
C1
C2
C3
Cluster

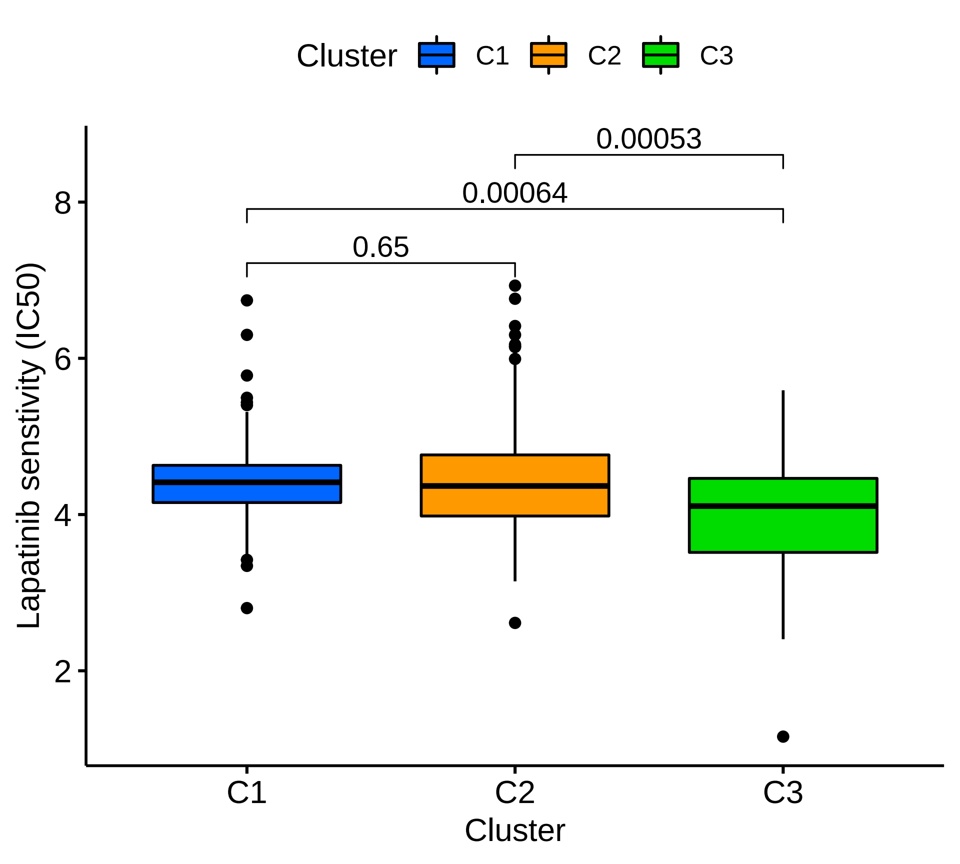

Cluster
C1
C2
C3
Lapatinib senstivity (IC50)
8
6
4
2
0.00053
0.00064
0.65
C1
C2
C3
Cluster

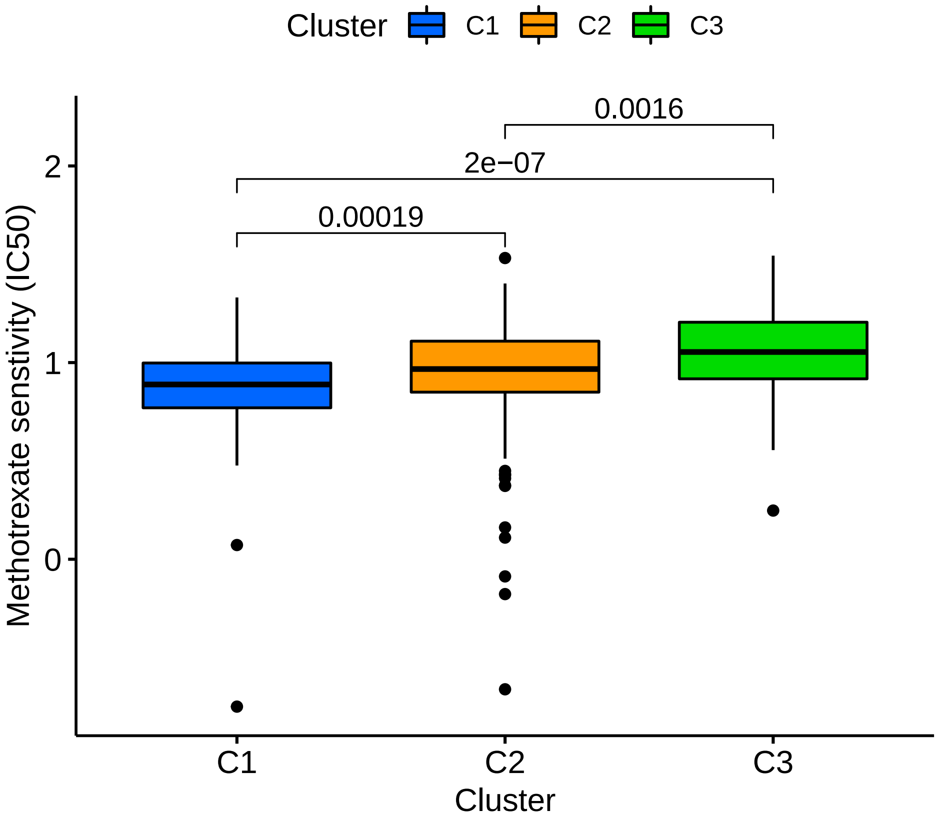

Cluster
C1
C2
C3
0.0016
2e−07
0.00019
Methotrexate senstivity (IC50)
2
1
0
C1
C2
C3
Cluster

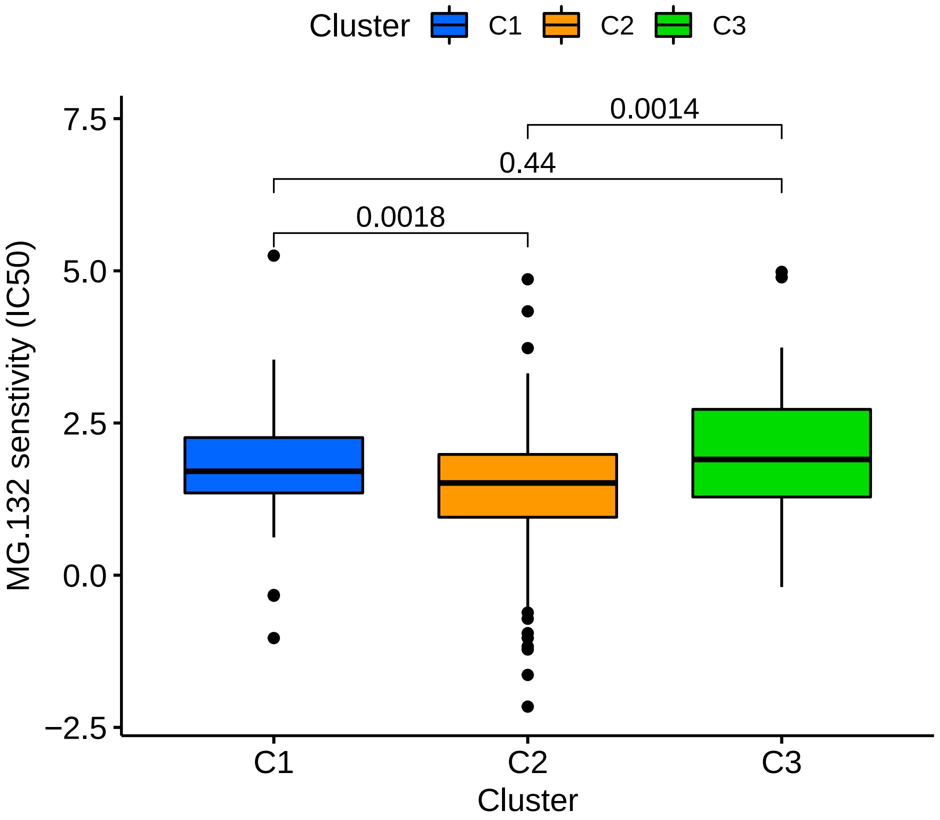

Cluster
C1
C2
C3
0.0014
0.44
0.0018
7.5
5.0
2.5
0.0
−2.5
MG.132 senstivity (IC50)
C1
C2
C3
Cluster

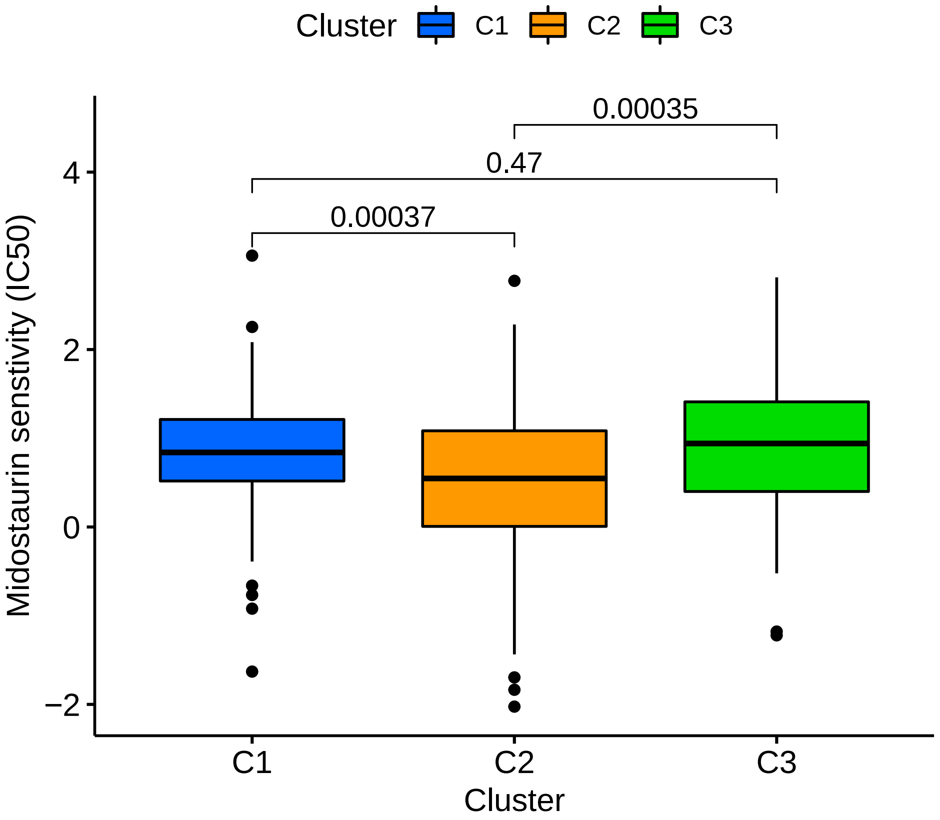

Cluster
C1
C2
C3
0.00035
0.47
0.00037
4
2
0
−2
Midostaurin senstivity (IC50)
C1
C2
C3
Cluster

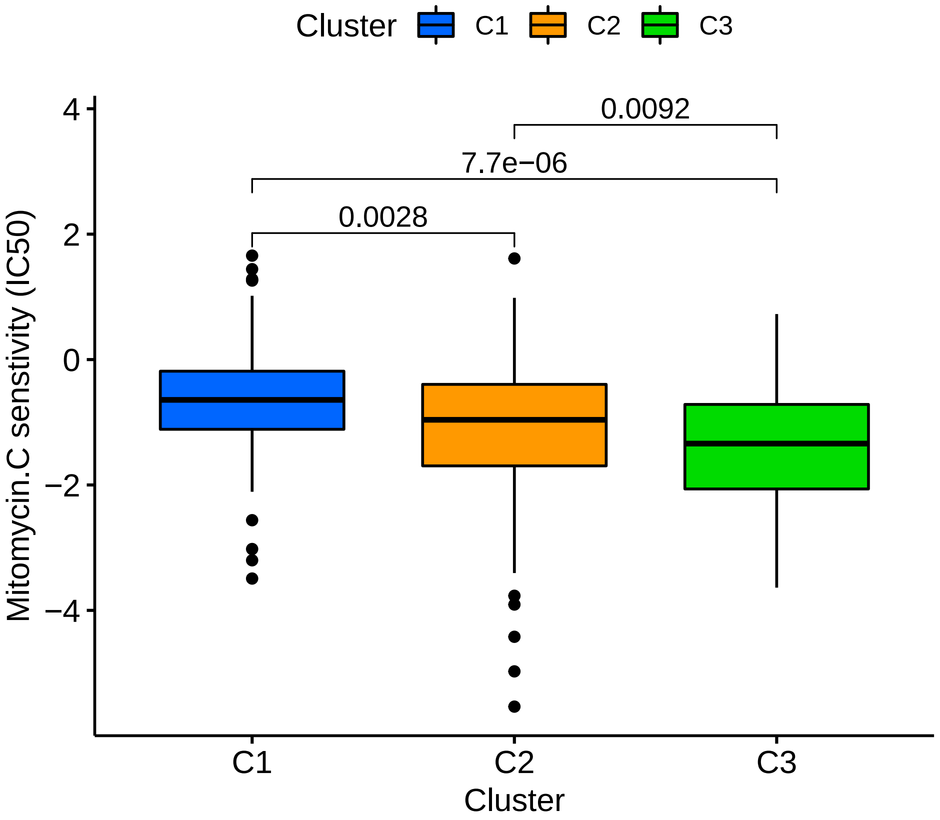

Cluster
C1
C2
C3
0.0092
7.7e−06
0.0028
4
2
0
−2
−4
Mitomycin.C senstivity (IC50)
C1
C2
C3
Cluster

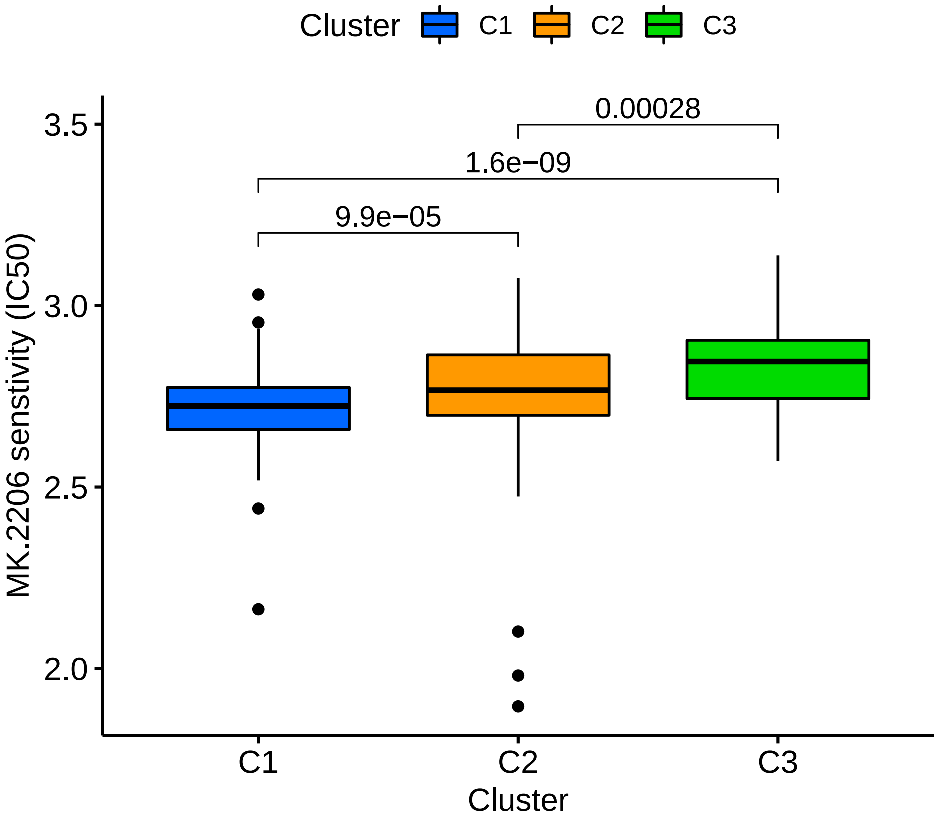

Cluster
C1
C2
C3
0.00028
1.6e−09
9.9e−05
3.5
3.0
2.5
2.0
MK.2206 senstivity (IC50)
C1
C2
C3
Cluster

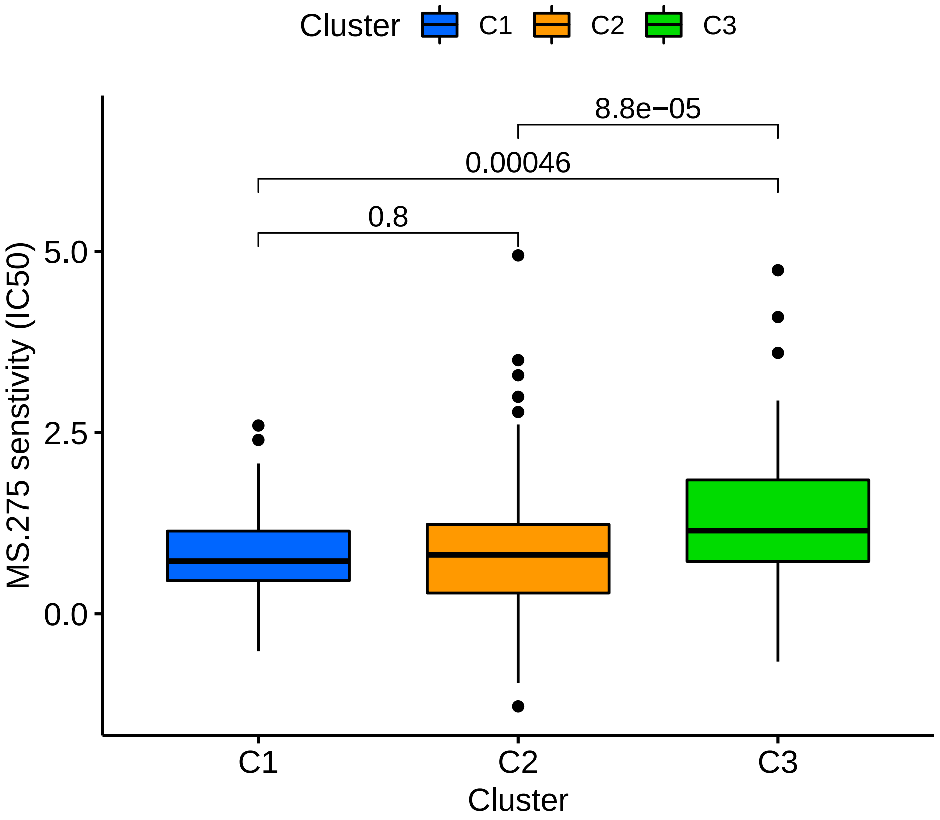
Cluster
C1
C2
C3
8.8e−05
0.00046
0.8
5.0
2.5
0.0
MS.275 senstivity (IC50)
C1
C2
C3
Cluster

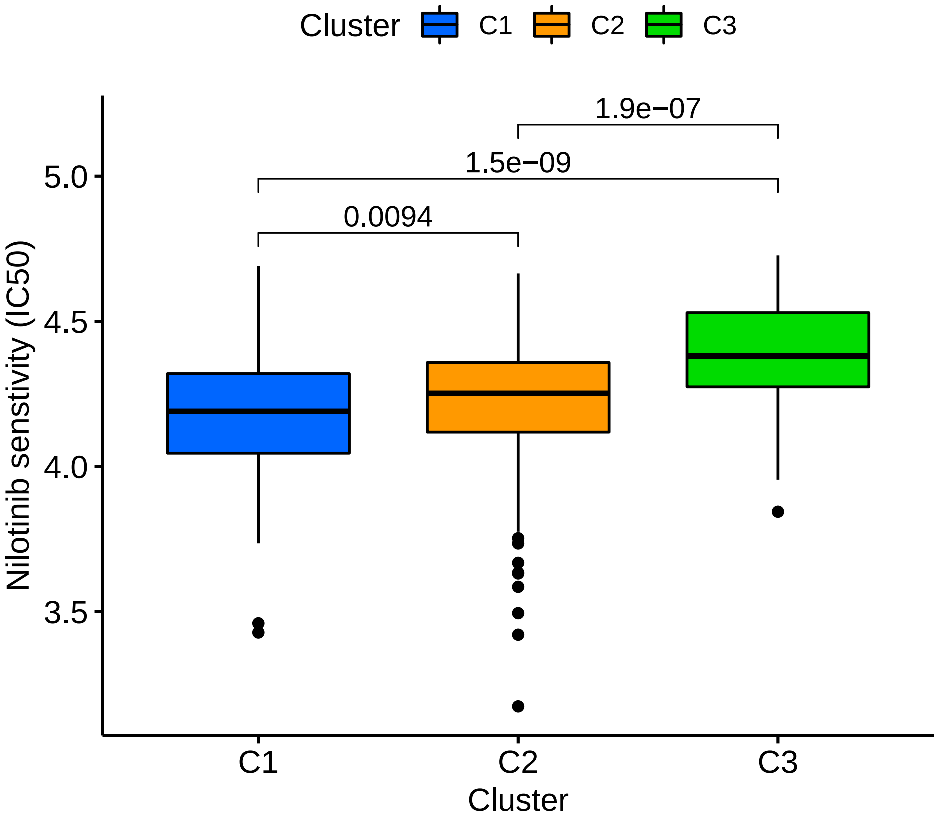

Cluster
C1
C2
C3
1.9e−07
1.5e−09
0.0094
5.0
4.5
4.0
3.5
Nilotinib senstivity (IC50)
C1
C2
C3
Cluster

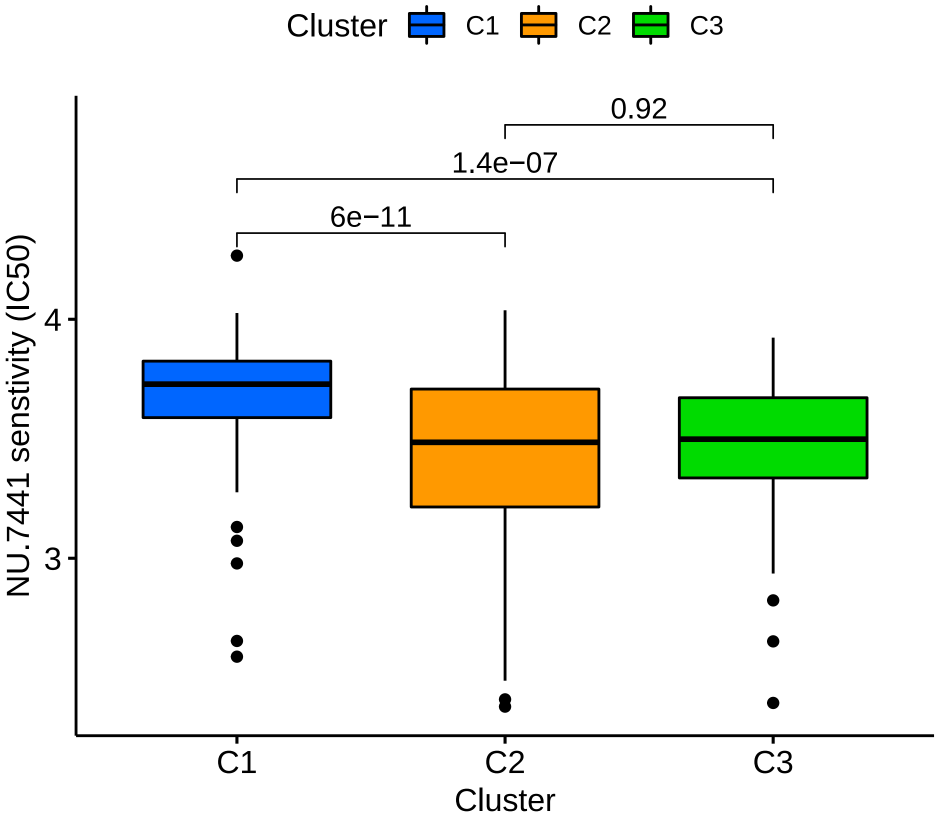
Cluster
C1
C2
C3
0.92
1.4e−07
6e−11
NU.7441 senstivity (IC50)
4
3
C1
C2
C3
Cluster

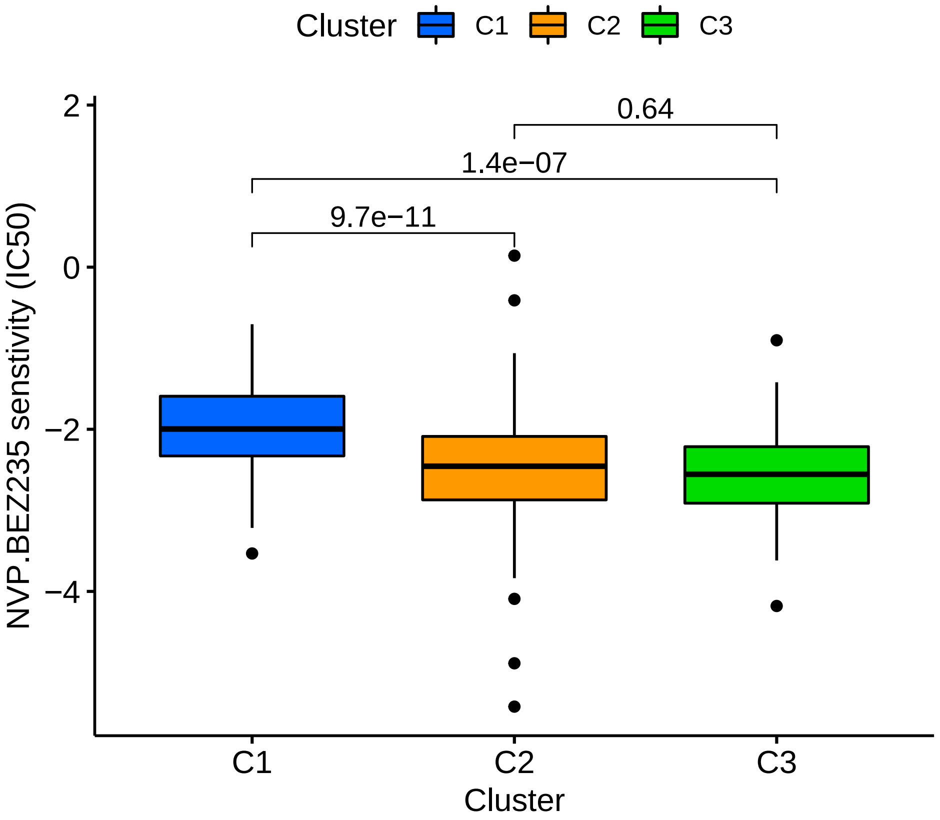

Cluster
C1
C2
C3
NVP.BEZ235 senstivity (IC50)
2
0
−2
−4
0.64
1.4e−07
9.7e−11
C1
C2
C3
Cluster

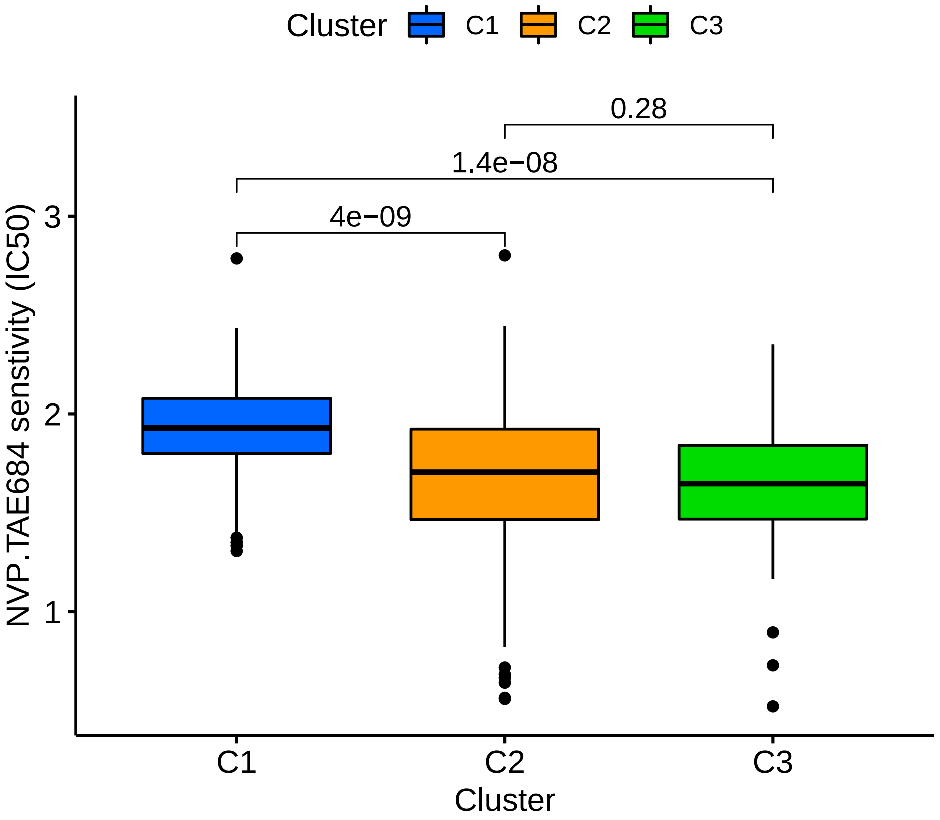

Cluster
C1
C2
C3
0.28
1.4e−08
4e−09
NVP.TAE684 senstivity (IC50)
3
2
1
C1
C2
C3
Cluster

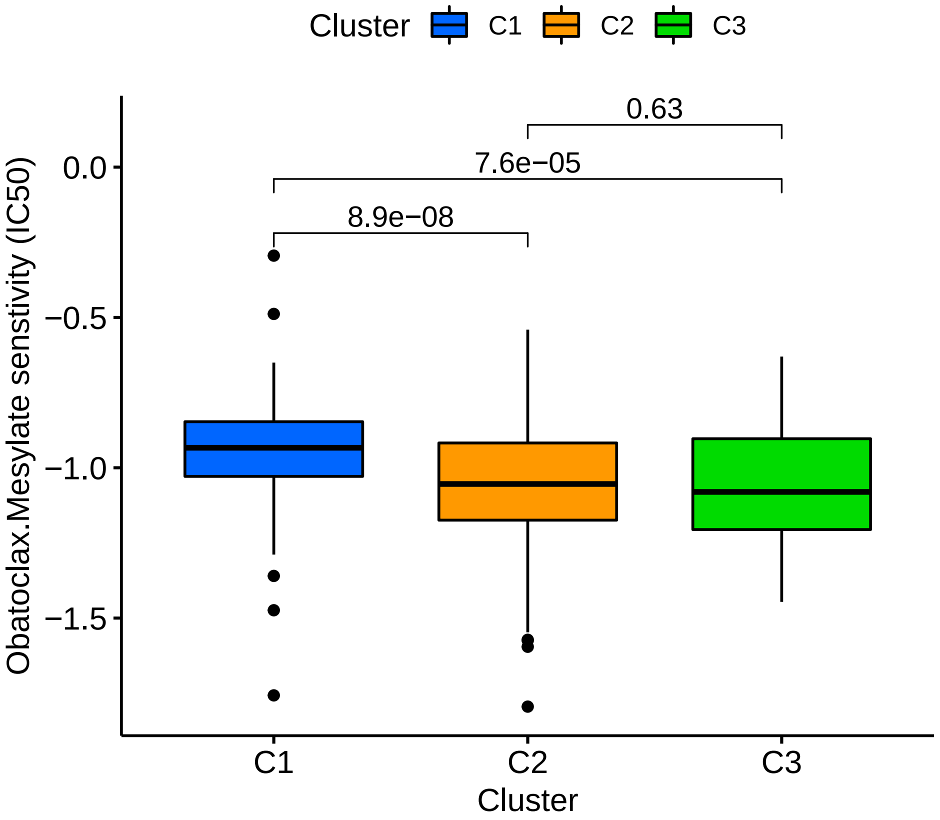

Cluster
C1
C2
C3
0.63
7.6e−05
8.9e−08
0.0
−0.5
−1.0
−1.5
Obatoclax.Mesylate senstivity (IC50)
C1
C2
C3
Cluster

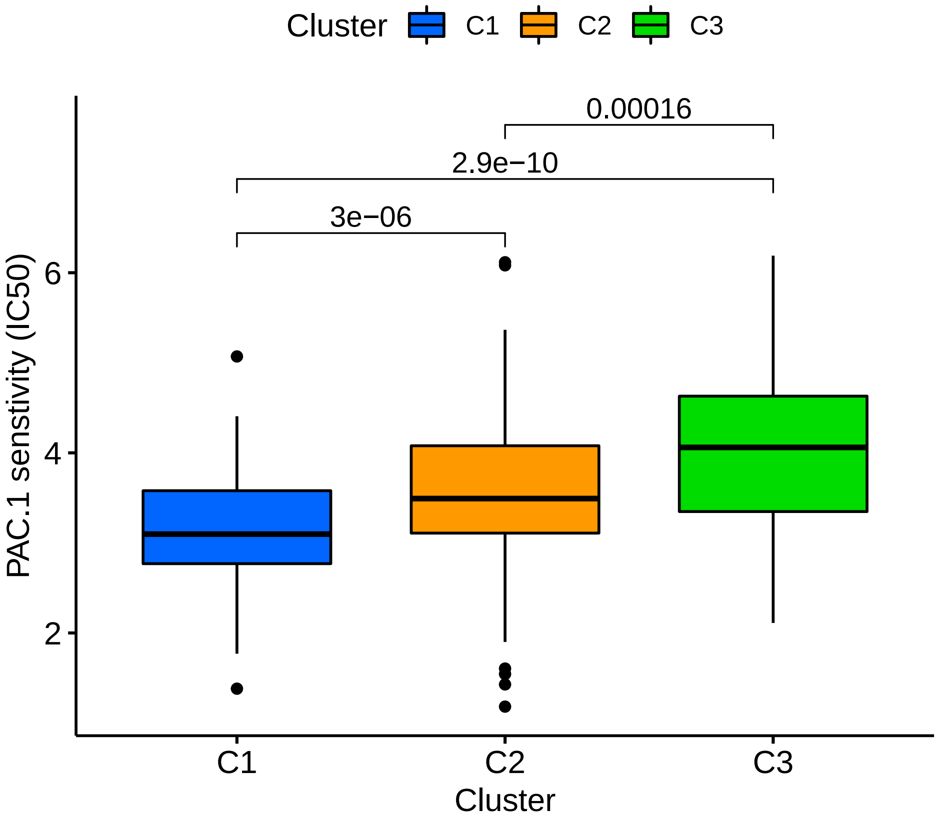

Cluster
C1
C2
C3
0.00016
2.9e−10
3e−06
PAC.1 senstivity (IC50)
6
4
2
C1
C2
C3
Cluster

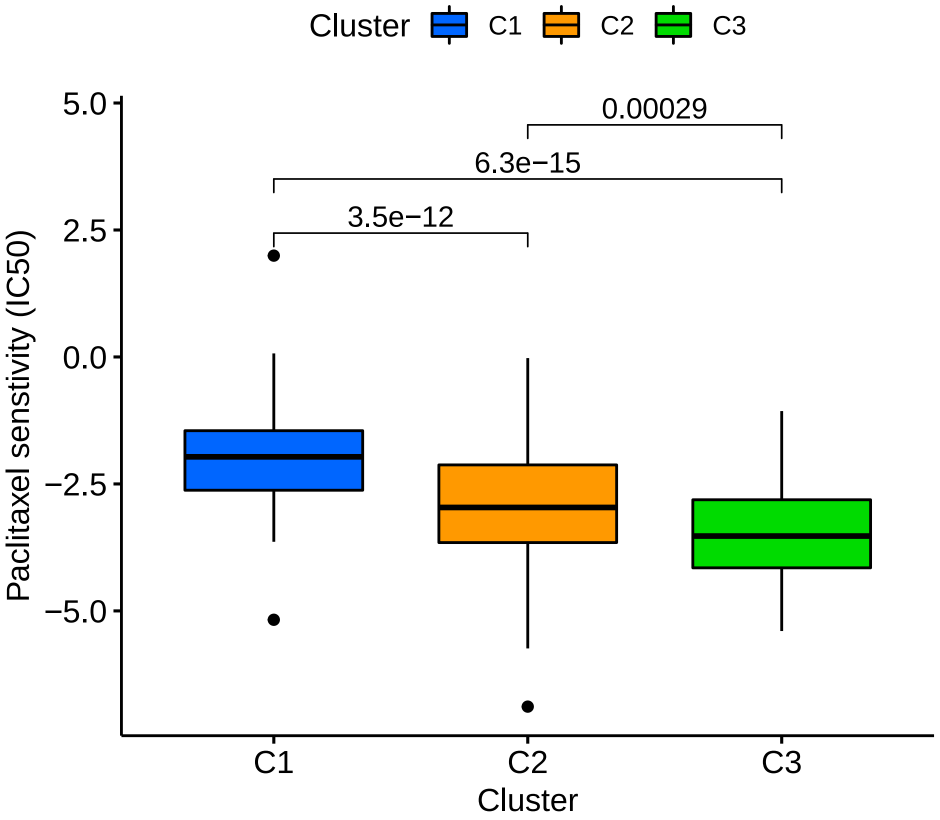

Cluster
C1
C2
C3
0.00029
6.3e−15
3.5e−12
5.0
2.5
0.0
−2.5
−5.0
Paclitaxel senstivity (IC50)
C1
C2
C3
Cluster

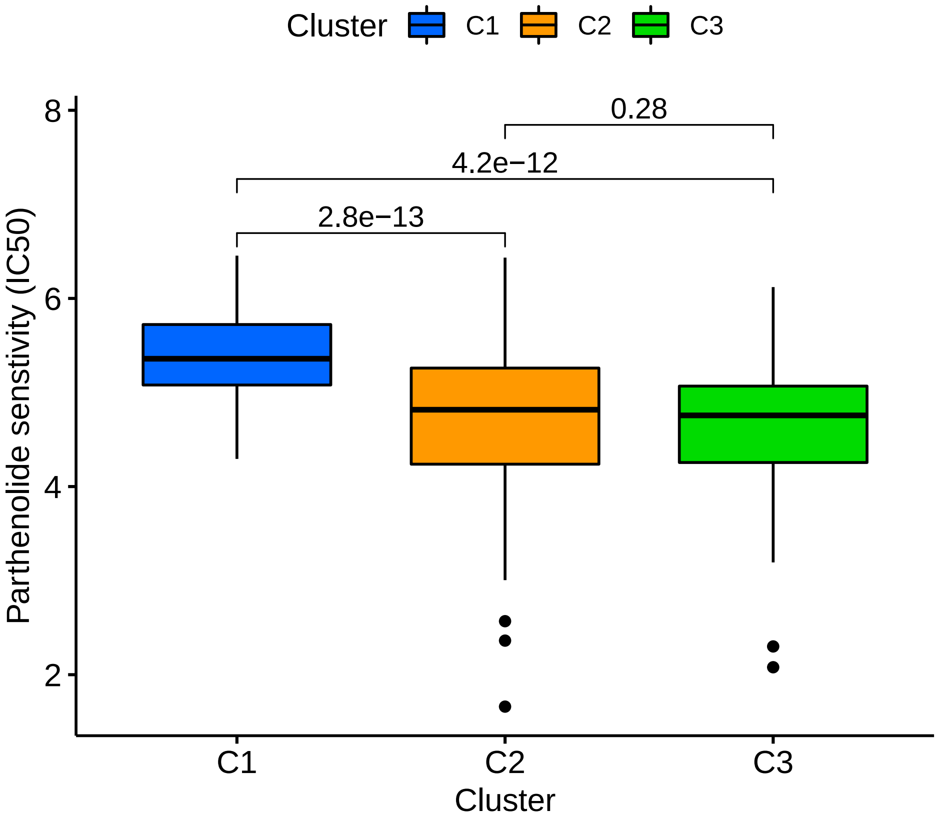

Cluster
C1
C2
C3
Parthenolide senstivity (IC50)
8
6
4
2
0.28
4.2e−12
2.8e−13
C1
C2
C3
Cluster

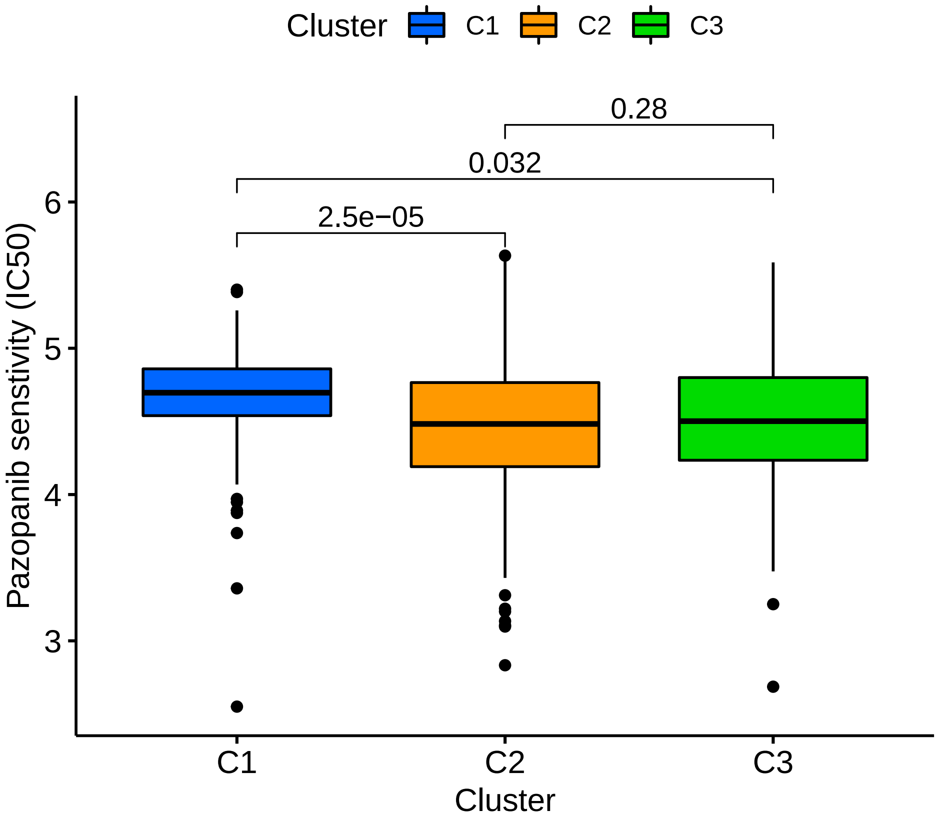

Cluster
C1
C2
C3
0.28
0.032
2.5e−05
6
5
4
3
Pazopanib senstivity (IC50)
C1
C2
C3
Cluster

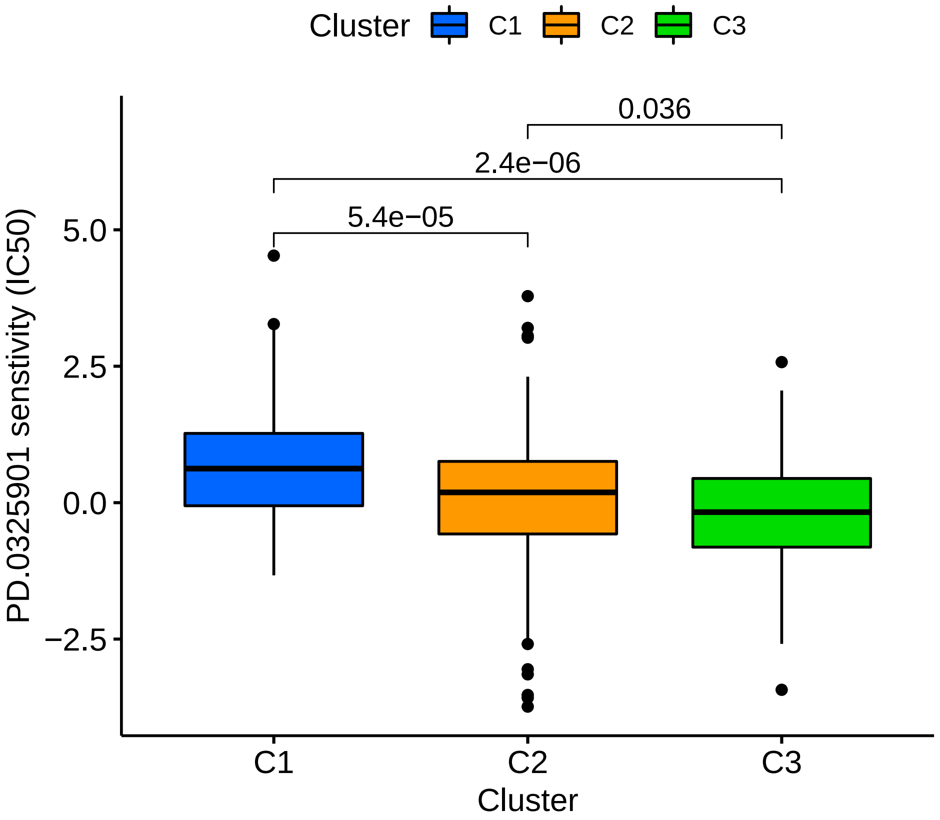

Cluster
C1
C2
C3
0.036
2.4e−06
5.4e−05
PD.0325901 senstivity (IC50)
5.0
2.5
0.0
−2.5
C1
C2
C3
Cluster

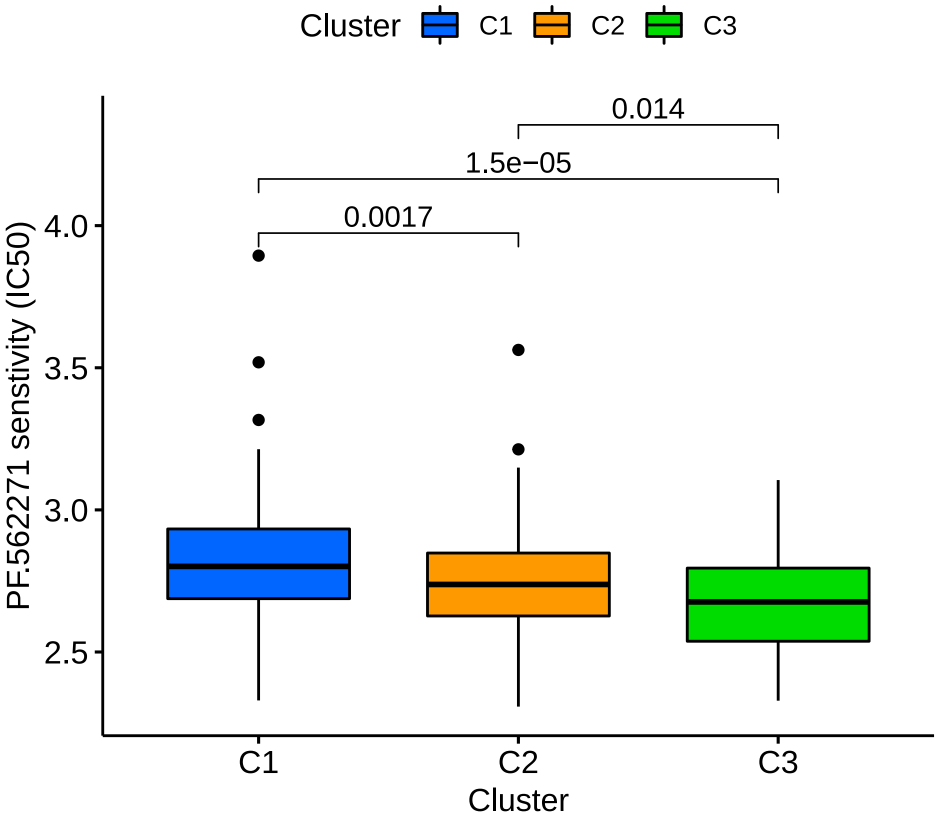
Cluster
C1
C2
C3
0.014
1.5e−05
0.0017
PF.562271 senstivity (IC50)
4.0
3.5
3.0
2.5
C1
C2
C3
Cluster

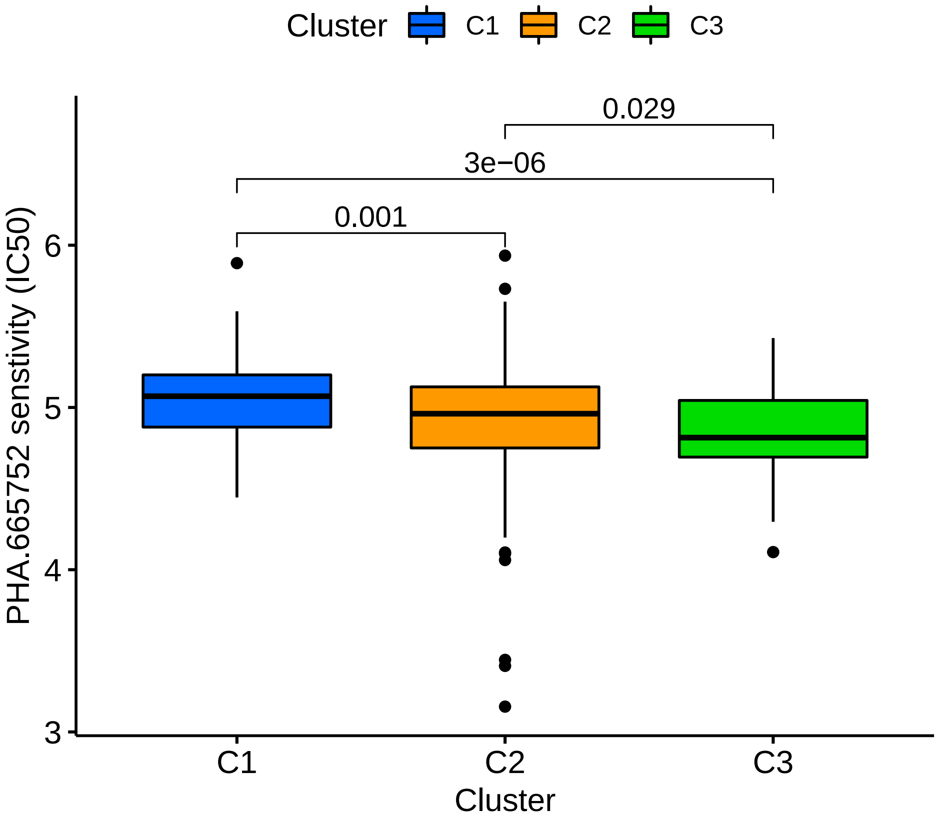
Cluster
C1
C2
C3
0.029
3e−06
0.001
PHA.665752 senstivity (IC50)
6
5
4
3
C1
C2
C3
Cluster

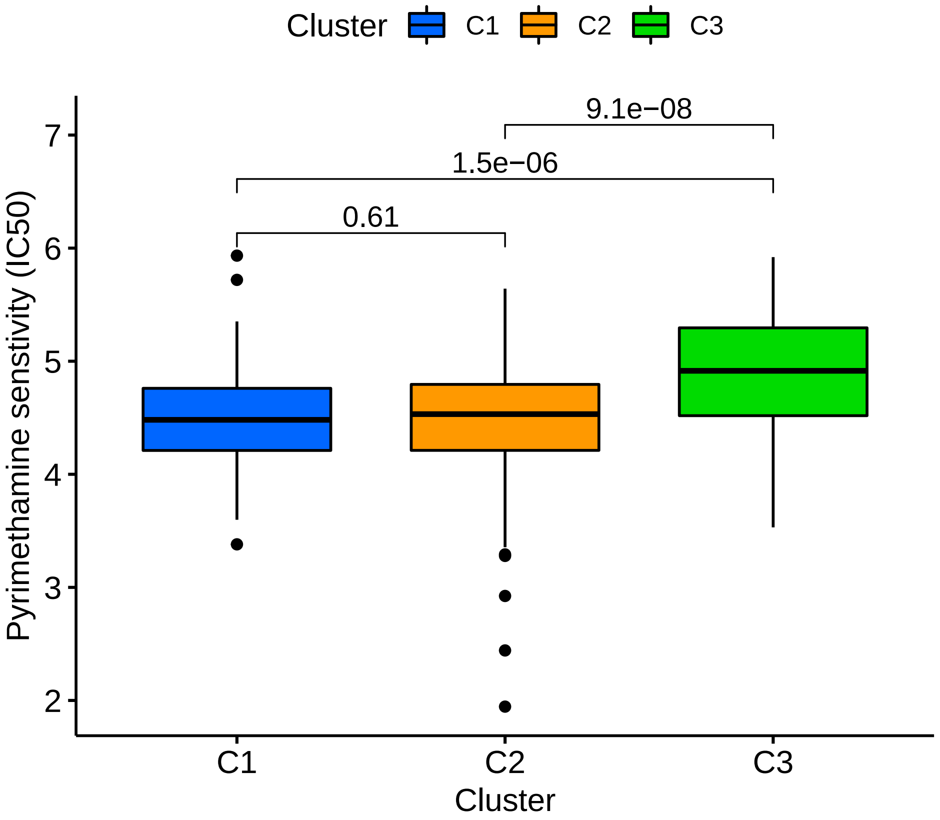

Cluster
C1
C2
C3
9.1e−08
1.5e−06
0.61
Pyrimethamine senstivity (IC50)
7
6
5
4
3
2
C1
C2
C3
Cluster

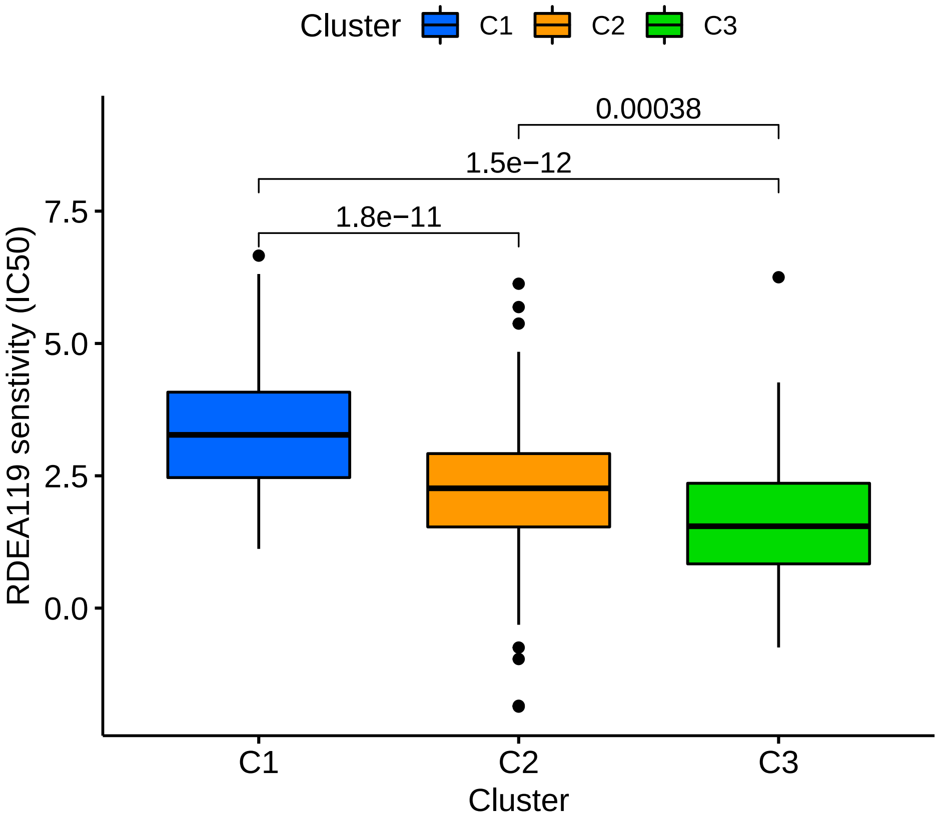

Cluster
C1
C2
C3
0.00038
1.5e−12
1.8e−11
RDEA119 senstivity (IC50)
7.5
5.0
2.5
0.0
C1
C2
C3
Cluster

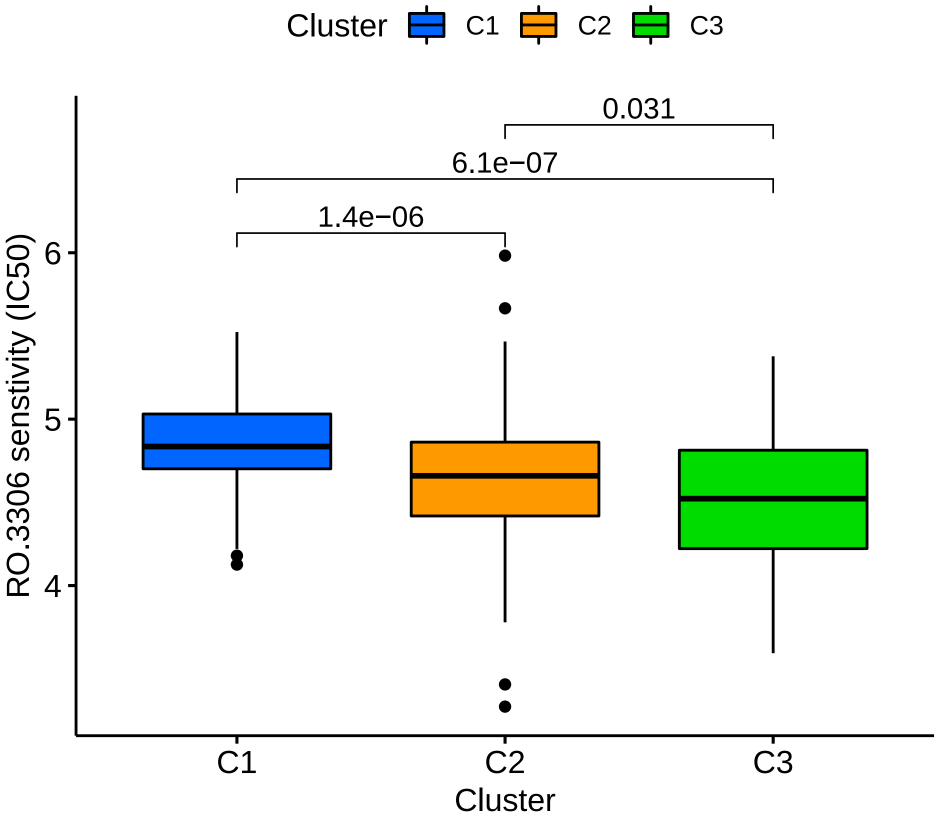
Cluster
C1
C2
C3
0.031
6.1e−07
1.4e−06
RO.3306 senstivity (IC50)
6
5
4
C1
C2
C3
Cluster

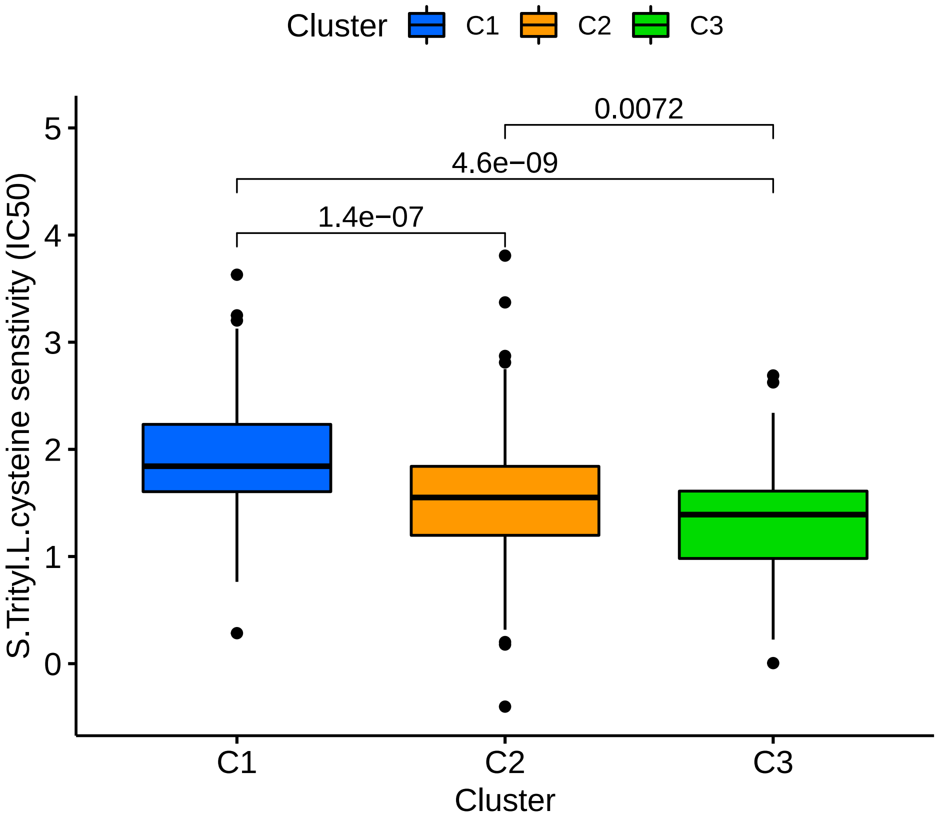

Cluster
C1
C2
C3
0.0072
4.6e−09
1.4e−07
S.Trityl.L.cysteine senstivity (IC50)
5
4
3
2
1
0
C1
C2
C3
Cluster

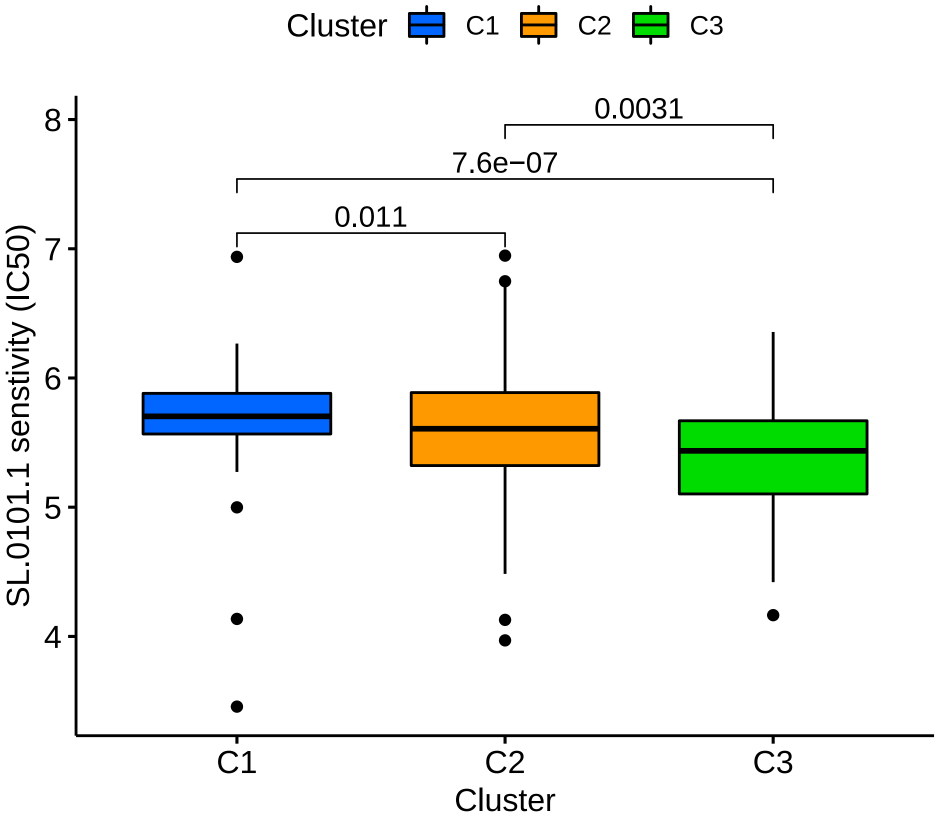

Cluster
C1
C2
C3
0.0031
7.6e−07
0.011
8
7
6
5
4
SL.0101.1 senstivity (IC50)
C1
C2
C3
Cluster

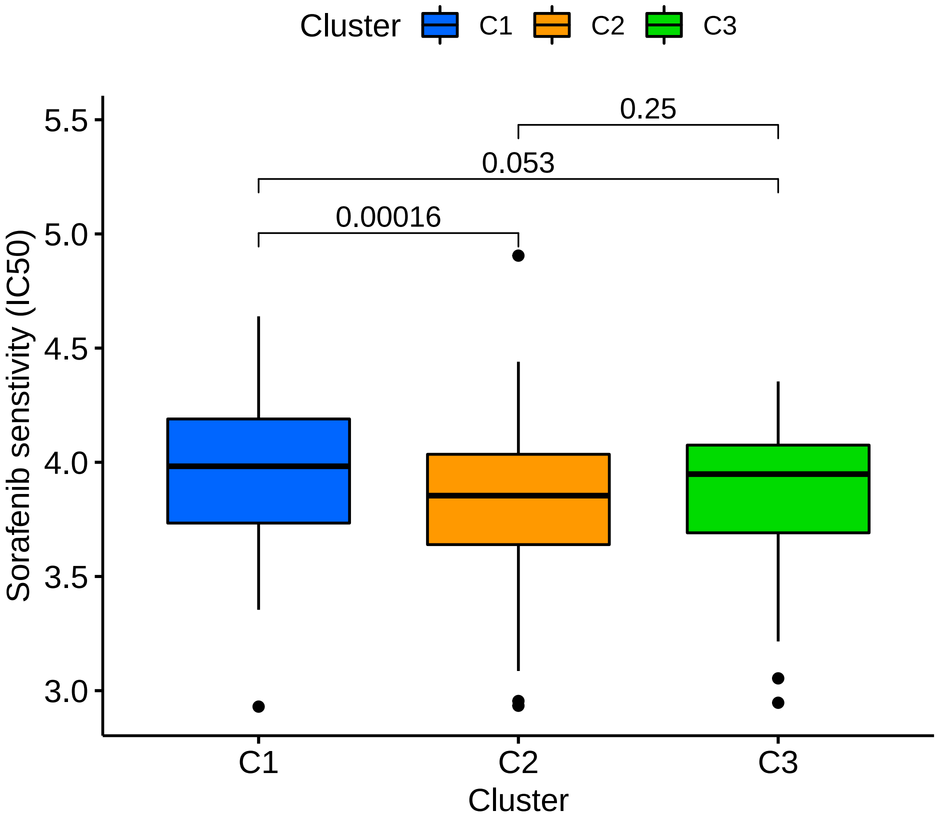

Cluster
C1
C2
C3
0.25
0.053
0.00016
5.5
5.0
4.5
4.0
3.5
3.0
Sorafenib senstivity (IC50)
C1
C2
C3
Cluster

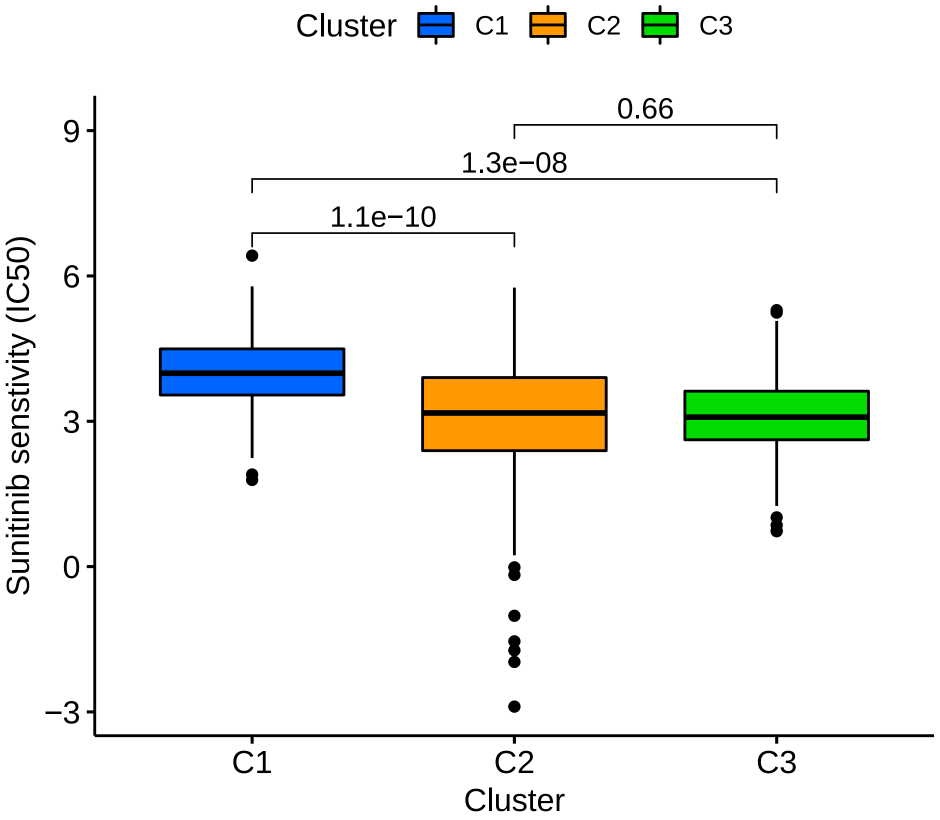
Cluster
C1
C2
C3
0.66
1.3e−08
1.1e−10
Sunitinib senstivity (IC50)
9
6
3
0
−3
C1
C2
C3
Cluster

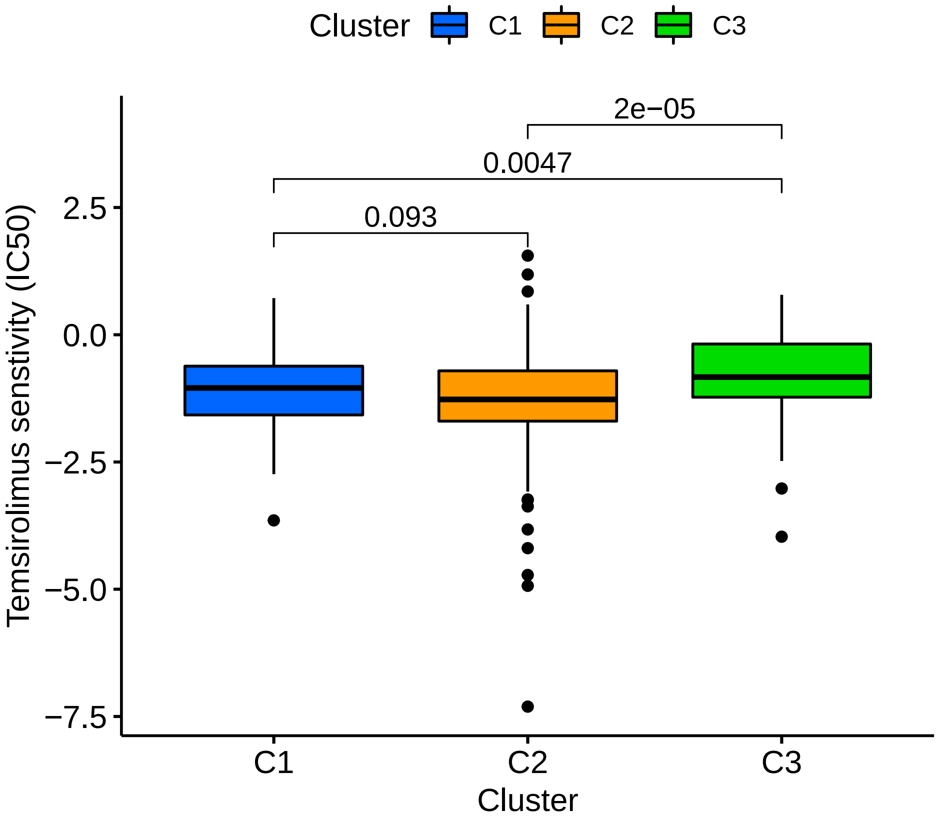

Cluster
C1
C2
C3
2e−05
0.0047
0.093
2.5
0.0
−2.5
−5.0
−7.5
Temsirolimus senstivity (IC50)
C1
C2
C3
Cluster

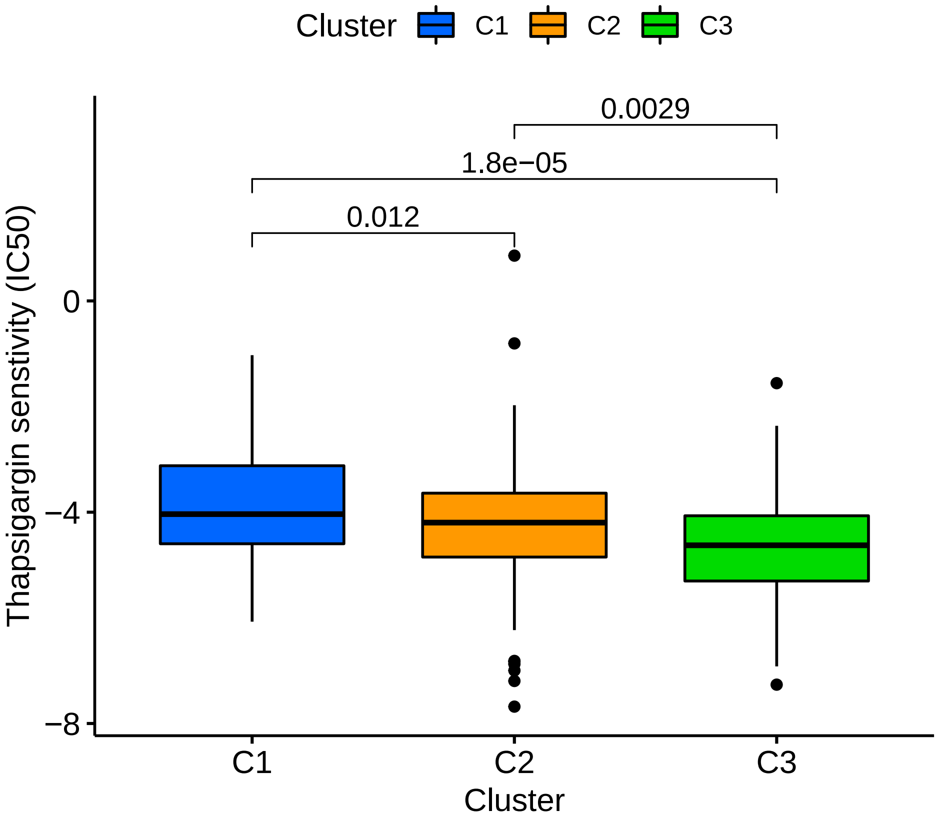

Cluster
C1
C2
C3
0.0029
1.8e−05
0.012
Thapsigargin senstivity (IC50)
0
−4
−8
C1
C2
C3
Cluster

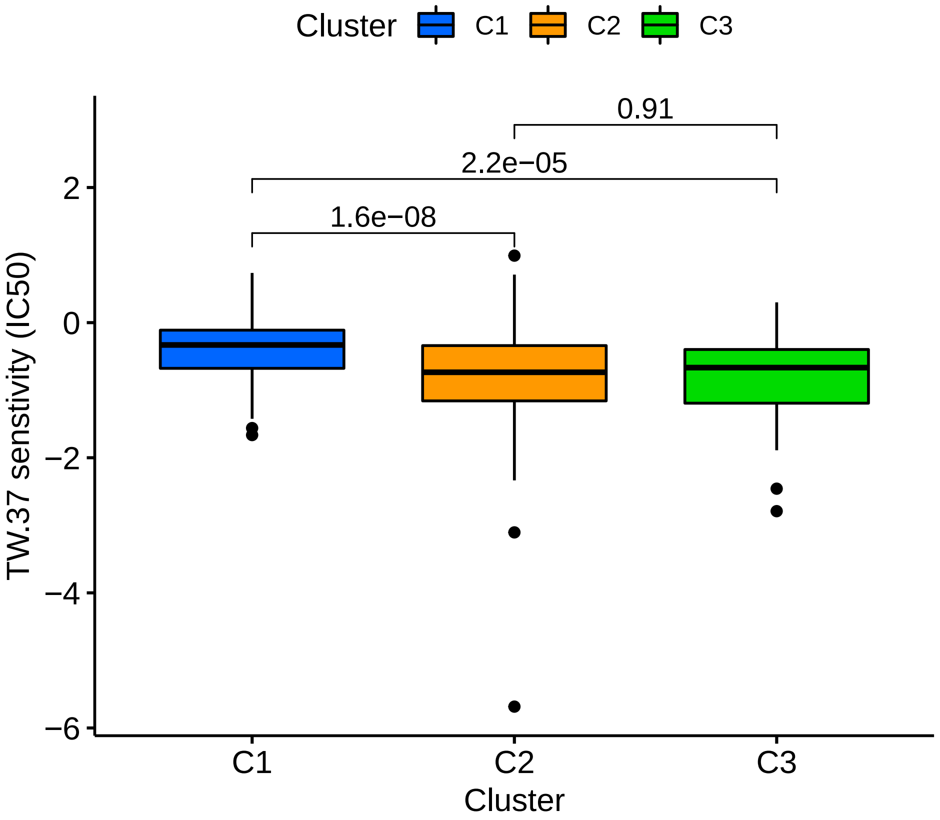
Cluster
C1
C2
C3
0.91
2.2e−05
1.6e−08
2
0
−2
−4
−6
TW.37 senstivity (IC50)
C1
C2
C3
Cluster

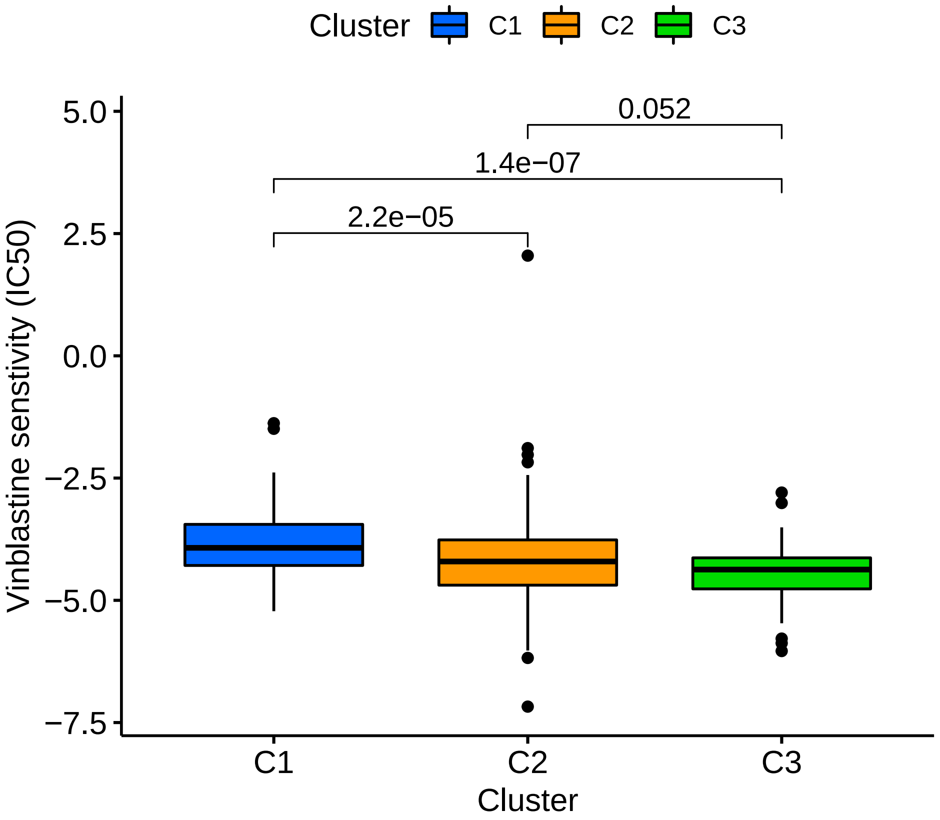

Cluster
C1
C2
C3
0.052
1.4e−07
2.2e−05
5.0
2.5
0.0
−2.5
−5.0
−7.5
Vinblastine senstivity (IC50)
C1
C2
C3
Cluster

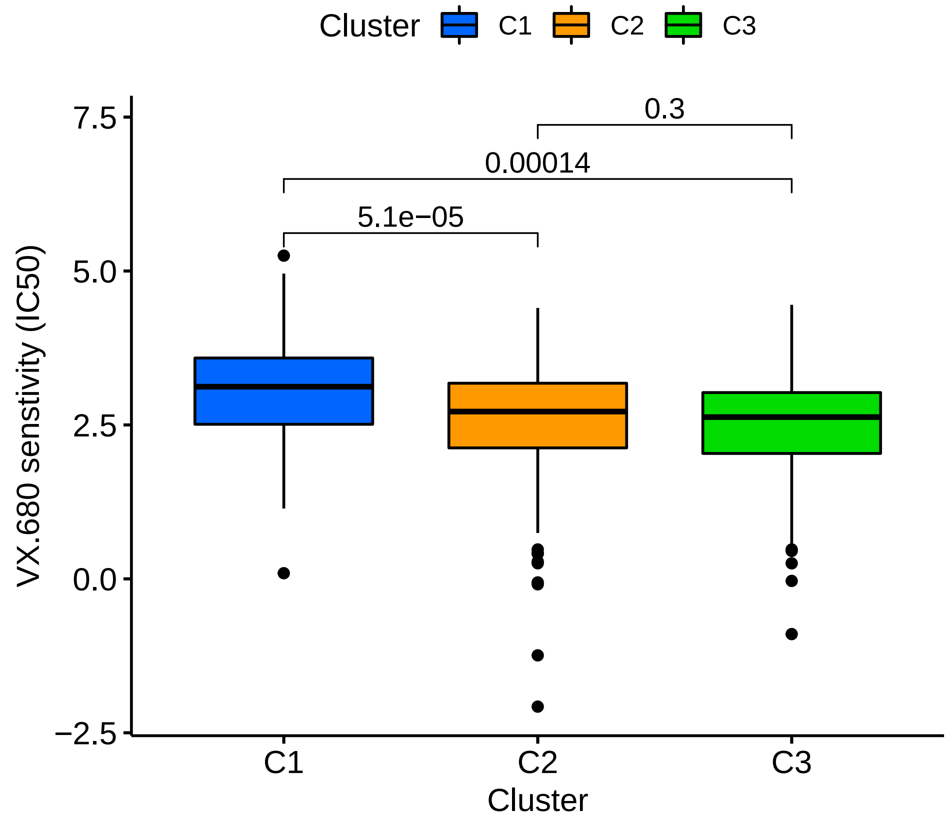

Cluster
C1
C2
C3
0.3
0.00014
5.1e−05
VX.680 senstivity (IC50)
7.5
5.0
2.5
0.0
−2.5
C1
C2
C3
Cluster

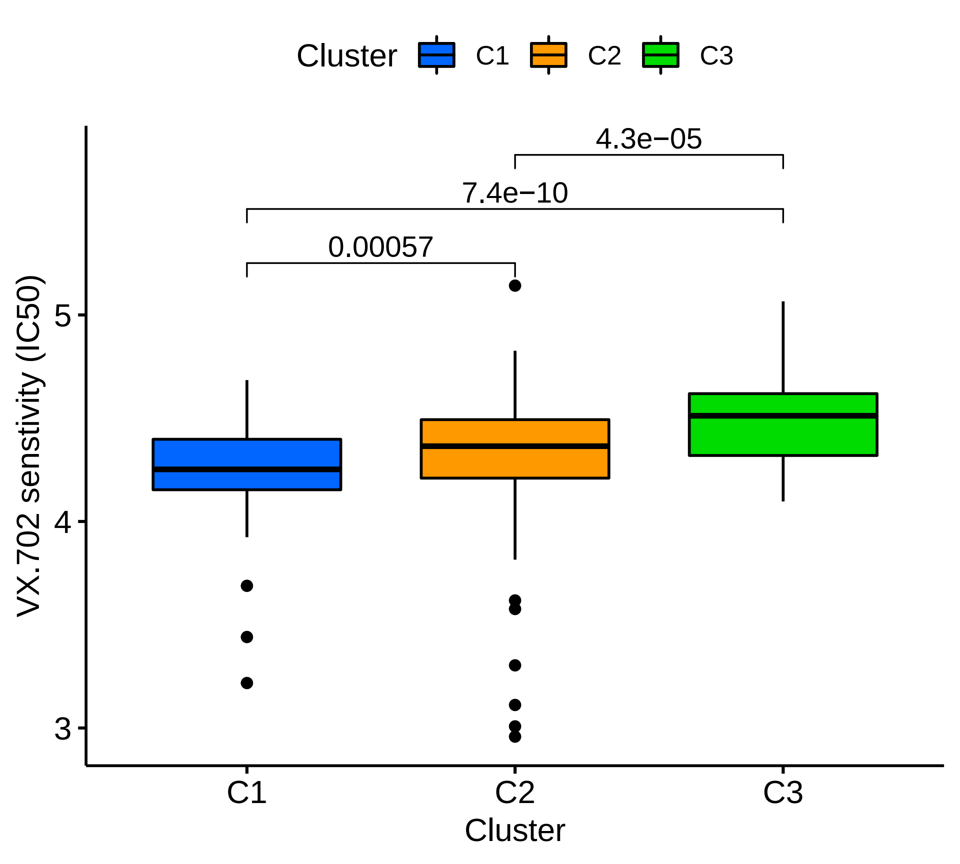

Cluster
C1
C2
C3
4.3e−05
7.4e−10
0.00057
VX.702 senstivity (IC50)
5
4
3
C1
C2
C3
Cluster

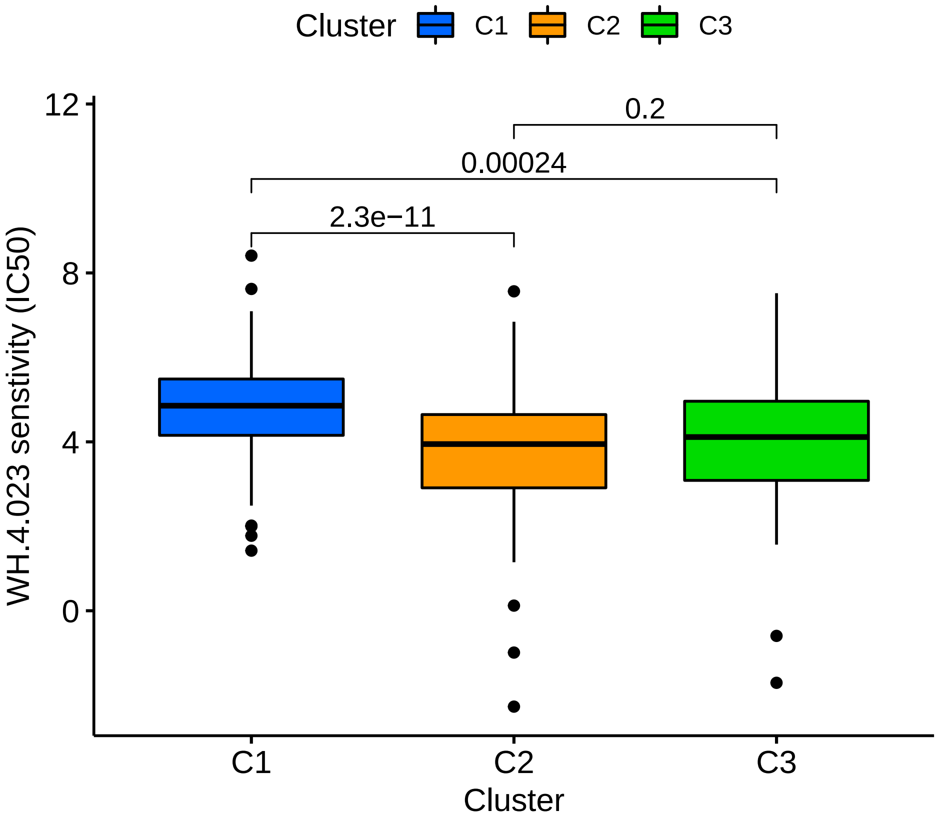

Cluster
C1
C2
C3
12
8
4
0
WH.4.023 senstivity (IC50)
0.2
0.00024
2.3e−11
C1
C2
C3
Cluster

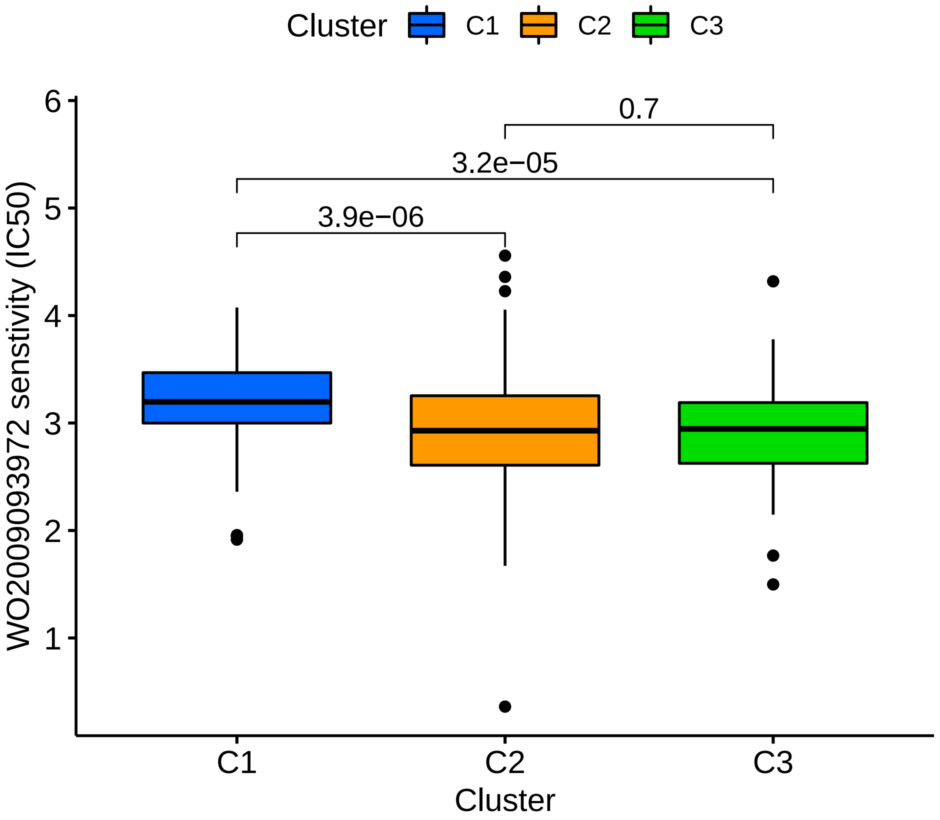
Cluster
C1
C2
C3
WO2009093972 senstivity (IC50)
6
5
4
3
2
1
0.7
3.2e−05
3.9e−06
C1
C2
C3
Cluster

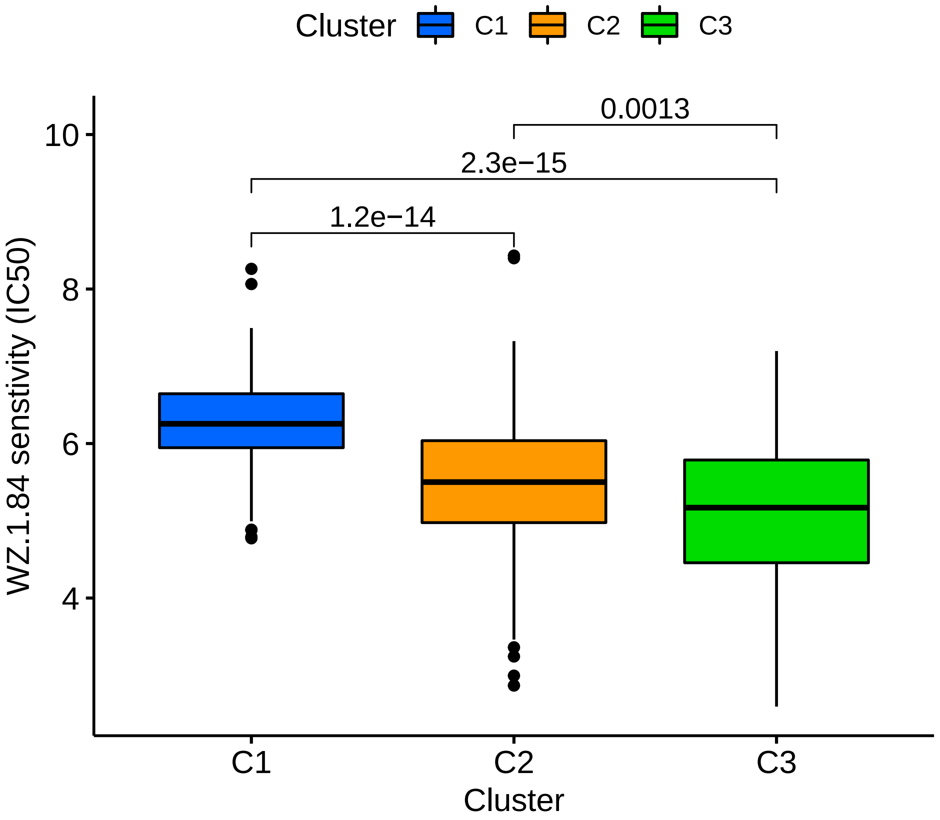

Cluster
C1
C2
C3
0.0013
2.3e−15
1.2e−14
10
8
6
4
WZ.1.84 senstivity (IC50)
C1
C2
C3
Cluster

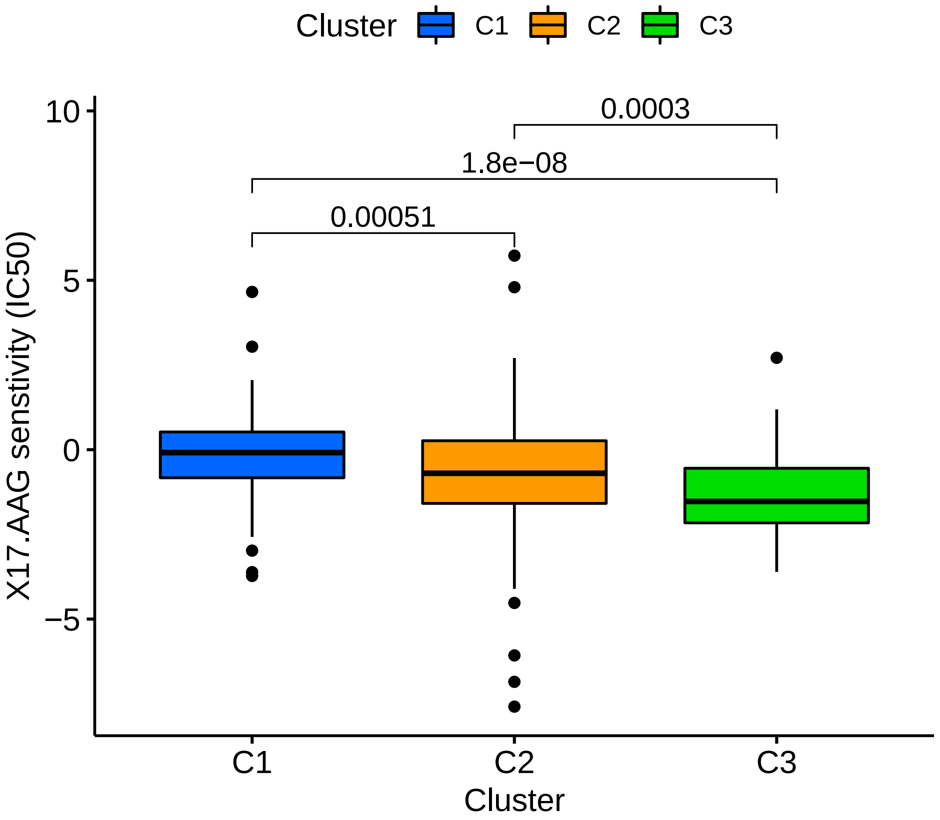
Cluster
C1
C2
C3
0.0003
1.8e−08
0.00051
10
5
0
−5
X17.AAG senstivity (IC50)
C1
C2
C3
Cluster

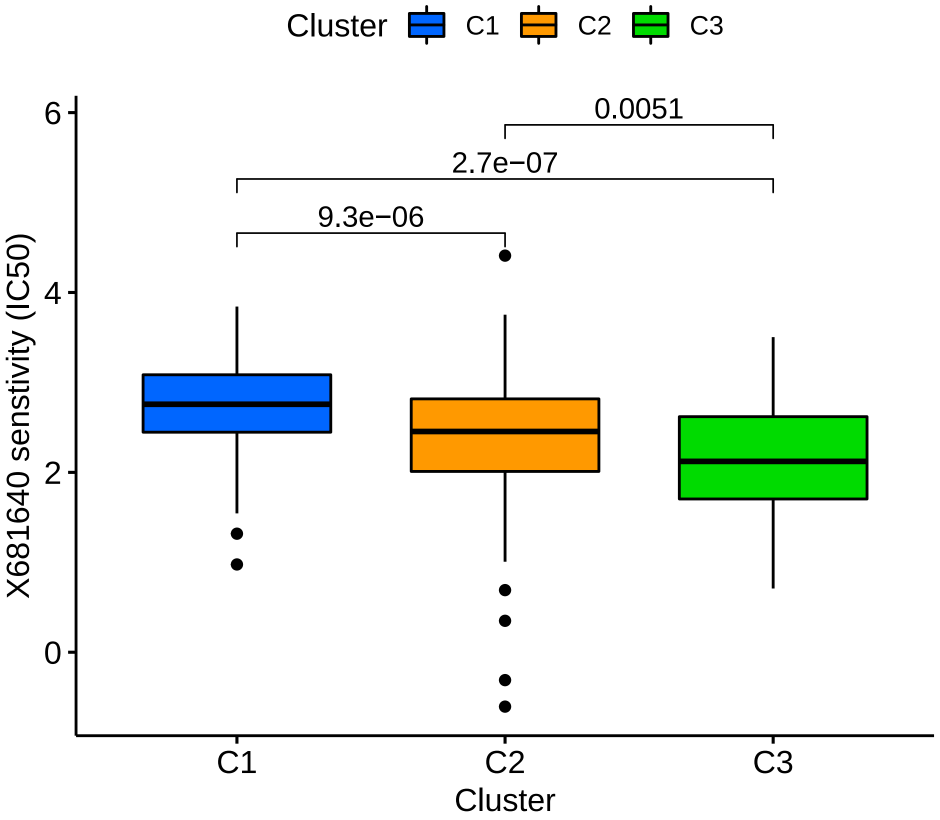

Cluster
C1
C2
C3
0.0051
2.7e−07
9.3e−06
6
4
2
0
X681640 senstivity (IC50)
C1
C2
C3
Cluster

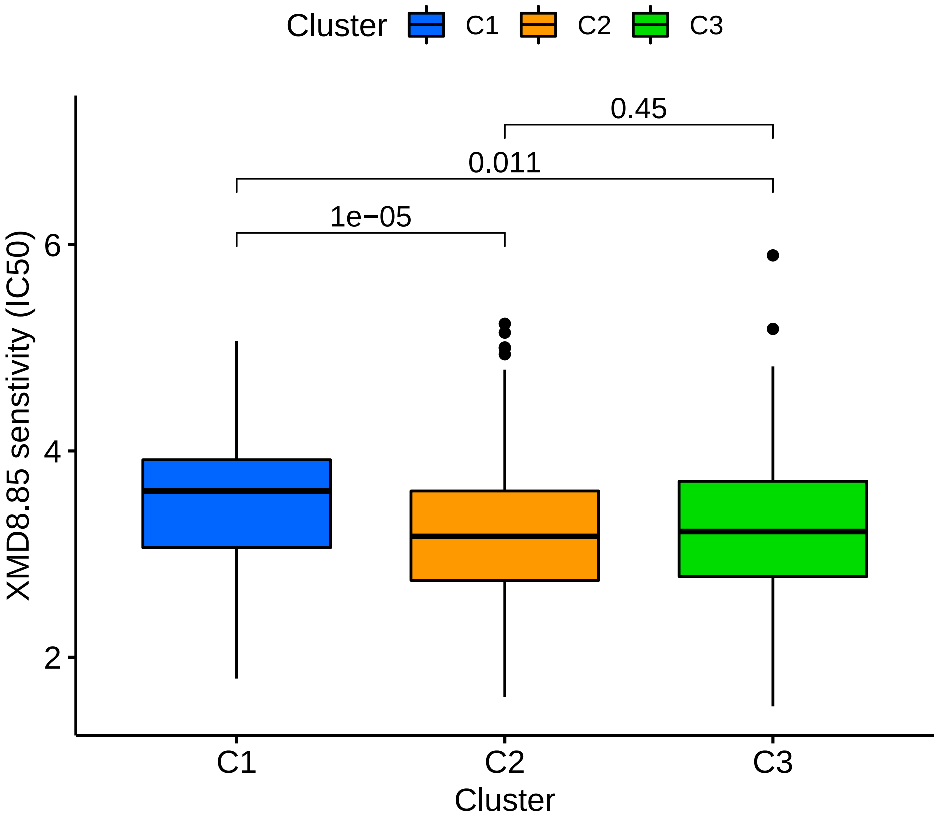
Cluster
C1
C2
C3
0.45
0.011
1e−05
6
4
2
XMD8.85 senstivity (IC50)
C1
C2
C3
Cluster
