# Supplementary figures and images for: Predicting Prognosis and Distinguishing Cold and Hot Tumors in Bladder Urothelial Carcinoma Based on Necroptosis-Associated lncRNAs
Source: Front Immunol. 2022 Jul 4;13:916800. doi: 10.3389/fimmu.2022.916800 (PMC9289196; doi:10.3389/fimmu.2022.916800)

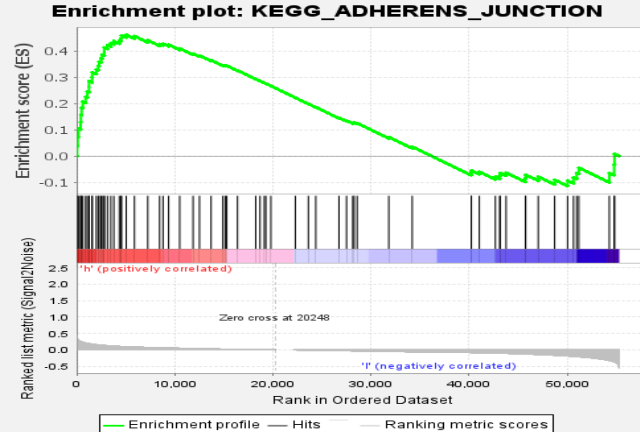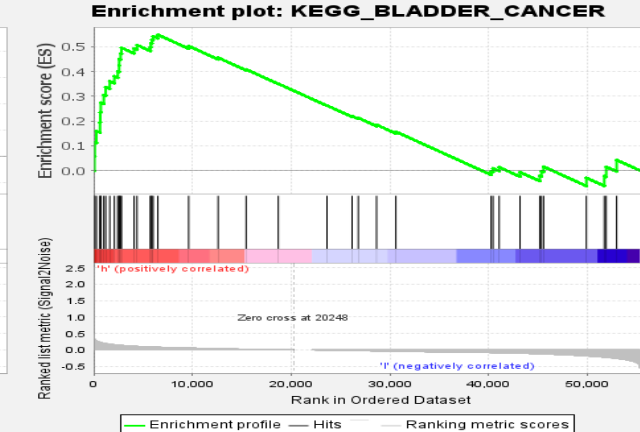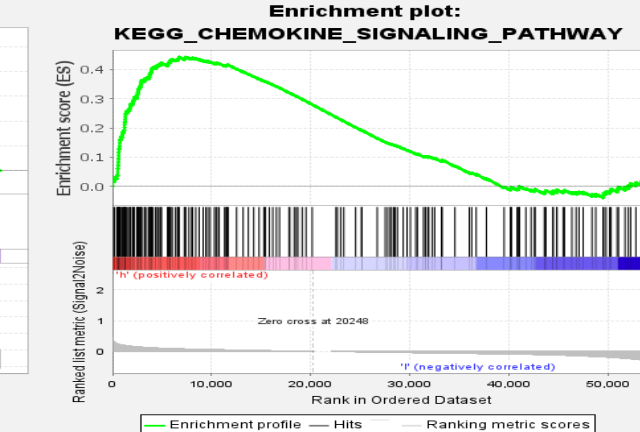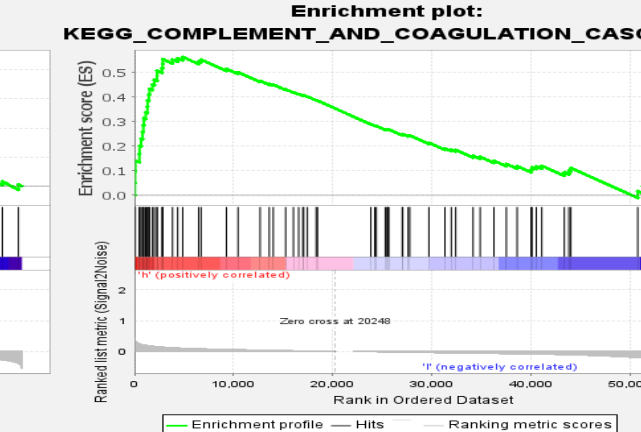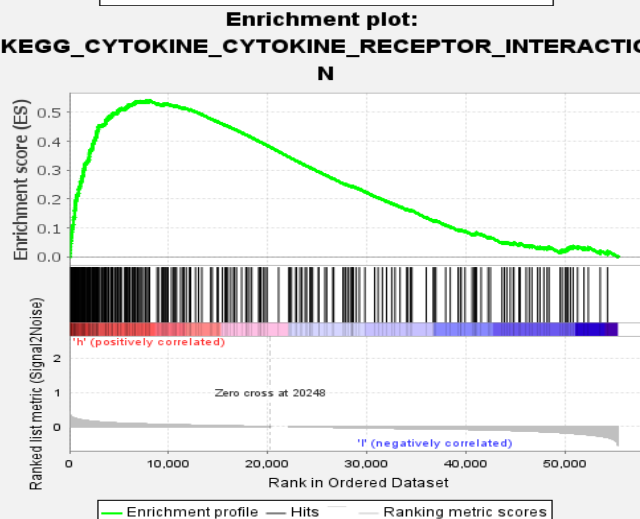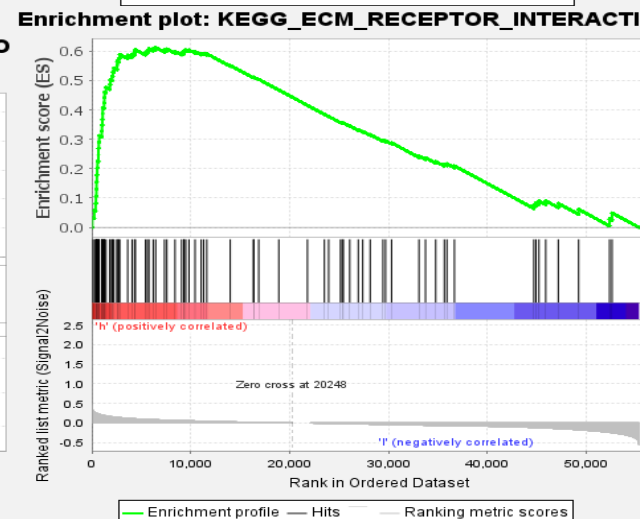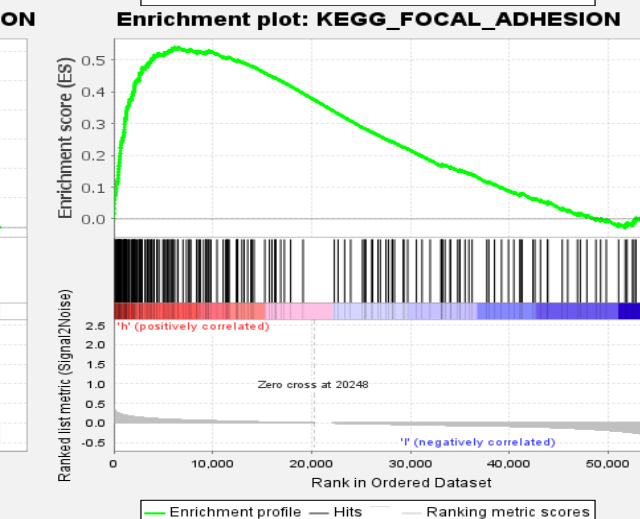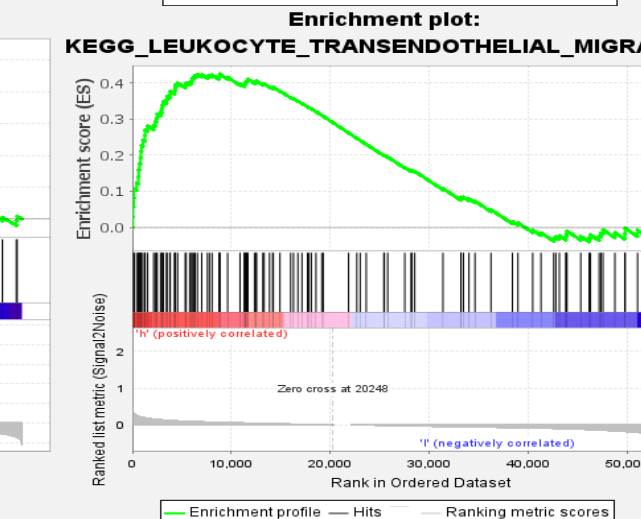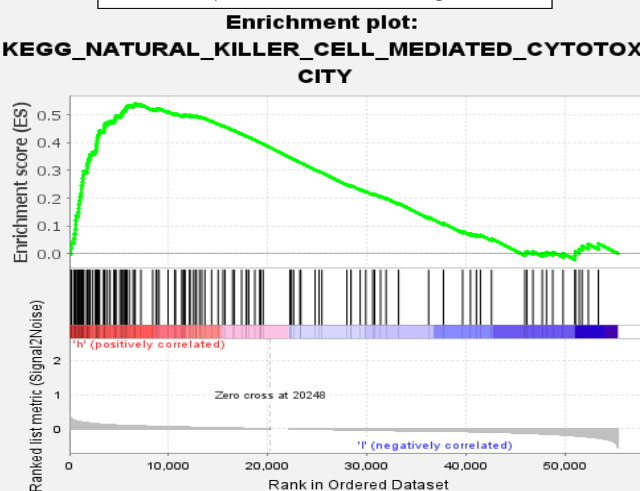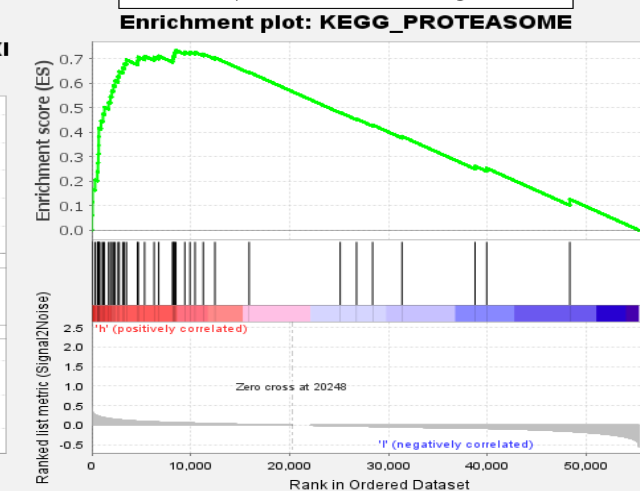

Supplement: Supplementary Figure 4 — The GSEA of the high-risk group. [file DataSheet_4.pdf]
